# The Impact of Histopathological Features on the Prognosis of Oral Squamous Cell Carcinoma: A Comprehensive Review and Meta-Analysis

**DOI:** 10.3389/fonc.2021.784924

**Published:** 2021-11-10

**Authors:** Eder da Silva Dolens, Mauricio Rocha Dourado, Alhadi Almangush, Tuula A. Salo, Clarissa Araujo Gurgel Rocha, Sabrina Daniela da Silva, Peter A. Brennan, Ricardo D. Coletta

**Affiliations:** ^1^ Graduate Program in Oral Biology, School of Dentistry, University of Campinas, Piracicaba, Brazil; ^2^ University of Western São Paulo (UNOESTE), Presidente Prudente, Brazil; ^3^ Department of Oral Diagnosis, School of Dentistry, University of Campinas, Piracicaba, Brazil; ^4^ Department of Oral and Maxillofacial Diseases, University of Helsinki, Helsinki, Finland; ^5^ Department of Pathology, University of Helsinki, Helsinki, Finland; ^6^ Cancer and Translational Medicine Research Unit, Medical Research Center Oulu, University of Oulu and Oulu University Hospital, Oulu, Finland; ^7^ Gonçalo Moniz Institute, Oswaldo Cruz Foundation, Salvador, Brazil; ^8^ Department of Propaedeutics, School of Dentistry, Federal University of Bahia, Bahia, Brazil; ^9^ Department of Otolaryngology Head and Neck Surgery, Sir Mortimer B. Davis-Jewish General Hospital, Faculty of Medicine, McGill University, Montreal, QC, Canada; ^10^ Segal Cancer Centre and Lady Davis Institute for Medical Research, Sir Mortimer B. Davis-Jewish General Hospital, Department of Experimental Medicine, Faculty of Medicine, McGill University, Montreal, QC, Canada; ^11^ Department of Oral and Maxillofacial Surgery, Queen Alexandra Hospital, Portsmouth, United Kingdom

**Keywords:** histopathological markers, prognosis, oral cancer, systematic review, meta-analysis

## Abstract

**Objective:**

Over many decades, studies on histopathological features have not only presented high-level evidence of contribution for treatment directions and prognosis of oral squamous cell carcinoma (OSCC) but also provided inconsistencies, making clinical application difficult. The 8th TNM staging system of OSCC has acknowledged the importance of some histopathological features, by incorporating depth of invasion (DOI) to T category and extranodal extension (ENE) to N category. The aim of this systematic review with meta-analysis is to determine the most clinically relevant histopathological features for risk assessment and treatment planning of OSCC and to elucidate gaps in the literature.

**Methods:**

A systematic review was conducted using PRISMA guidelines, and the eligibility criteria were based on population, exposure, comparison, outcome, and study type (PECOS). PubMed, Cochrane, Scopus, and Web of Science were searched for articles exploring the impact of histopathological features on OSCC outcomes with Cox multivariate analysis. Pooled data were subjected to an inverse variance method with random effects or fixed effect model, and the risk of bias was evaluated using quality in prognosis studies (QUIPS). Quality of evidence was assessed with the Grading of Recommendations Assessment, Development, and Evaluation (GRADE) criteria.

**Results:**

The study included 172 articles published from 1999 to 2021. Meta-analyses confirmed the prognostic potential of DOI, ENE, perineural invasion, lymphovascular invasion, and involvement of the surgical margins and brought promising results for the association of bone invasion, tumor thickness, and pattern of invasion with increased risk for poor survival. Although with a small number of studies, the results also revealed a clinical significance of tumor budding and tumor-stroma ratio on predicted survival of patients with OSCC. Most of the studies were considered with low or moderate risk of bias, and the certainty in evidence varied from very low to high.

**Conclusion:**

Our results confirm the potential prognostic usefulness of many histopathological features and highlight the promising results of others; however, further studies are advised to apply consistent designs, filling in the literature gaps to the pertinence of histopathological markers for OSCC prognosis.

**Systematic Review Registration:**

International Prospective Register of Systematic Reviews (PROSPERO), identifier CRD42020219630.

## Introduction

Oral cancer, represented as oral squamous cell carcinomas (OSCC) in more than 90% of all oral cavity malignancies, is an important cause of cancer mortality worldwide, with an estimated 177,000 deaths every year (1). It is considered an aggressive cancer, with a 5-year overall survival rate of approximately 50%, reduced to less than 30% in advanced stages (2). Tumor extension, lymph node metastasis, high rates of locoregional recurrence, and development of second primary tumors are the leading causes of death in OSCC patients (3). The survival rates vary from developed to developing countries due mainly to late diagnosis and inappropriate access to the latest advances to the therapeutic options in developing countries (4). Moreover, the lack of biomarkers to precisely characterize the disease in an accurate prognostic level, which could indicate the best treatment for each patient and posttherapeutic monitoring, contribute to this unfavorable scenario that persists for decades (4).

Nowadays, the standard care for OSCC is surgery associated with adjuvant radiotherapy with or without chemotherapy. In addition, immunotherapy and targeted therapy are showing very promising results and have been approved to patients with recurrent/metastatic disease (5). The therapeutic plan and the estimation of prognosis for OSCC patients still rely on the disease stage, but it does not appropriately reflect the biological behavior of this heterogeneous group of tumors. Even at an early stage, specifically tumors of the tongue and floor of mouth may be very aggressive, with increased tendency to invasion, metastasis, and consequently poor prognosis (6). Many different biomarkers have been proposed as prognostic indicators for OSCC, and among those are specific histopathological features of the tumors. Indeed, those microscopic features, individually or combined in a grading system, are one of the oldest potential biomarkers to be studied for OSCC. The AJCC/UICC 8th TNM staging system, revised in 2017, has introduced two major histological characteristics in the T and N staging (7). The depth of invasion (DOI), which represents the distance from the basement membrane of epithelium to the deepest area of invasive front of the tumor, was incorporated in the T category, and the extranodal extension (ENE), defined as the extension of metastatic tumor cells through the lymph nodal capsule, was included in the N category. Although both can be determined clinically or through imaging examinations, the histological evaluation is essential, particularly when those features are not prominently detected (8, 9).

Several other histopathological characteristics, including the traditional perineural invasion (PNI), lymphovascular invasion (LVI), status of the surgical margins, pattern of invasion, bone invasion, tumor thickness and inflammatory response, and the more recently described features such as tumor budding and tumor-stroma ratio (TSR), were evaluated in many studies in relation to their prognostic impact or as indicators of necessity of a multimodality treatment strategy in OSCCs. However, studies have used different metrics and cutoff parameters, and even definitions may vary for these, generating inconsistent results. The classification of PNI is a good example, since criteria may vary from tumor cells invading the perineurium (inside nervus) to tumor cells surrounding a nerve (only adjacent to the structure) (10). As the associations of some of these histopathological features with survival in OSCC remain controversial, and there are inconsistencies in the definitions and scales/cutoff, this systematic review and meta-analysis aims to provide the state of our current understanding about histopathological features as potential biomarkers for OSCC, critically analyzing the gaps in knowledge that limit these biomarkers use in the clinical practice.

## Material and Methods

This study was carried out following the Preferred Reporting Items for Systematic reviews and Meta-Analyses (PRISMA) guidelines. The protocol on its methodology was registered in the International Prospective Register of Systematic Reviews (PROSPERO), under the number CRD42020219630.

### Eligibility Criteria

The main objective was to determine the impact of histopathological features on prognosis of OSCCs. The PECOS approach was used to conceptualize the search strategy for this review as follows: P (participants)—patients with OSCC, E (exposition)—analysis of histological parameters based on hematoxylin and eosin (HE)-stained slides, C (comparison or control)—not applicable, O (outcome)—overall survival (OS), disease-specific survival (DSS) or disease-free survival (DFS) based on Cox multivariate regression analysis, S (types of studies)—observational/cohort studies. Exclusion criteria included: (1) studies based on tumors different than OSCC, (2) studies based on a sample composed of biopsies, (3) studies based on immunohistochemistry, (4) studies involving histological grading systems, (5) studies reporting only univariate survival analysis, (6) studies based only on association analysis, (7) studies that did not report the HR and/or its 95% confidence interval (CI), (8) reviews of the literature, letters and conference abstracts, (9) if full-length articles were not available, (10) articles not written in English, and (11) studies with sample reported in another study already included in this review.

### Database Search, Study Selection, and Data Collection

Initially, the search was conducted on PubMed, Cochrane, Scopus, and Web of Science and included all articles published until December 2020. However, a second literature search was performed using the same terms at the beginning of August 2021, retrieving articles published between January and July 2021. The Grey literature and the reference lists from included studies were also searched. The keywords and the search strategy are depicted in **Supplementary File S1**. The articles in duplicate were removed, and references were transferred to the Rayyan QCRI software for systematic reviews (https://rayyan.qcri.org). The title and abstract screening and the full-text screening were completed by two independent reviewers, and the disagreements were resolved with the inclusion of a third reviewer. The information extracted from each study included the first author and year of publication, country where the samples were collected, number of cases, location and TNM clinical stage of tumors, histological parameters, and HR and 95% CI based on multivariate analysis. All data were collected by one reviewer and cross-checked by another.

### Risk of Bias and Quality of Evidence

The risk of bias was evaluated using the Quality in Prognosis Studies (QUIPS) tool (11) adjusted for this review. The features under the six domains of QUIPS tool were evaluated for each selected study by the two reviewers, and disagreements were resolved after consensus discussion. To be considered a study with low risk of bias, all six domains should be rated as low or moderate, with at least four rated as low. If two or more of the domains were scored as high, the study was classified as high risk of bias, and in the remaining conditions, the studies were scored as moderate risk of bias. To assess the overall quality of evidence of each histological parameter, the Grading of Recommendations Assessment, Development, and Evaluation (GRADE) was used (12), with the aid of software GRADEpro Guideline Development Tool (https://gradepro.org/).

### Statistical Analysis

The meta-analyses were conducted using the Review Manager (RevMan, version 5.4.1, Cochrane), applying an inverse variance method with random effects or fixed effect model. If *I* ^2^ was >50%, random effects model was used, whereas fixed effect model was applied if *I* ^2^ was <50%. The studies were pooled based on the outcome reported in the studies, such as OS, DSS, and DFS. As the description of recurrence was heterogeneous and included local recurrence-free survival (LRFS), neck recurrence-free survival (NRFS), distant recurrence-free survival (DRFS), and loco-regional recurrence-free survival (LRRFS), all were combined under DFS. When possible, subgroup analyses were undertaken based on a specific cutoff of parameter.

## Results

### Study Selection

**Figure 1:**
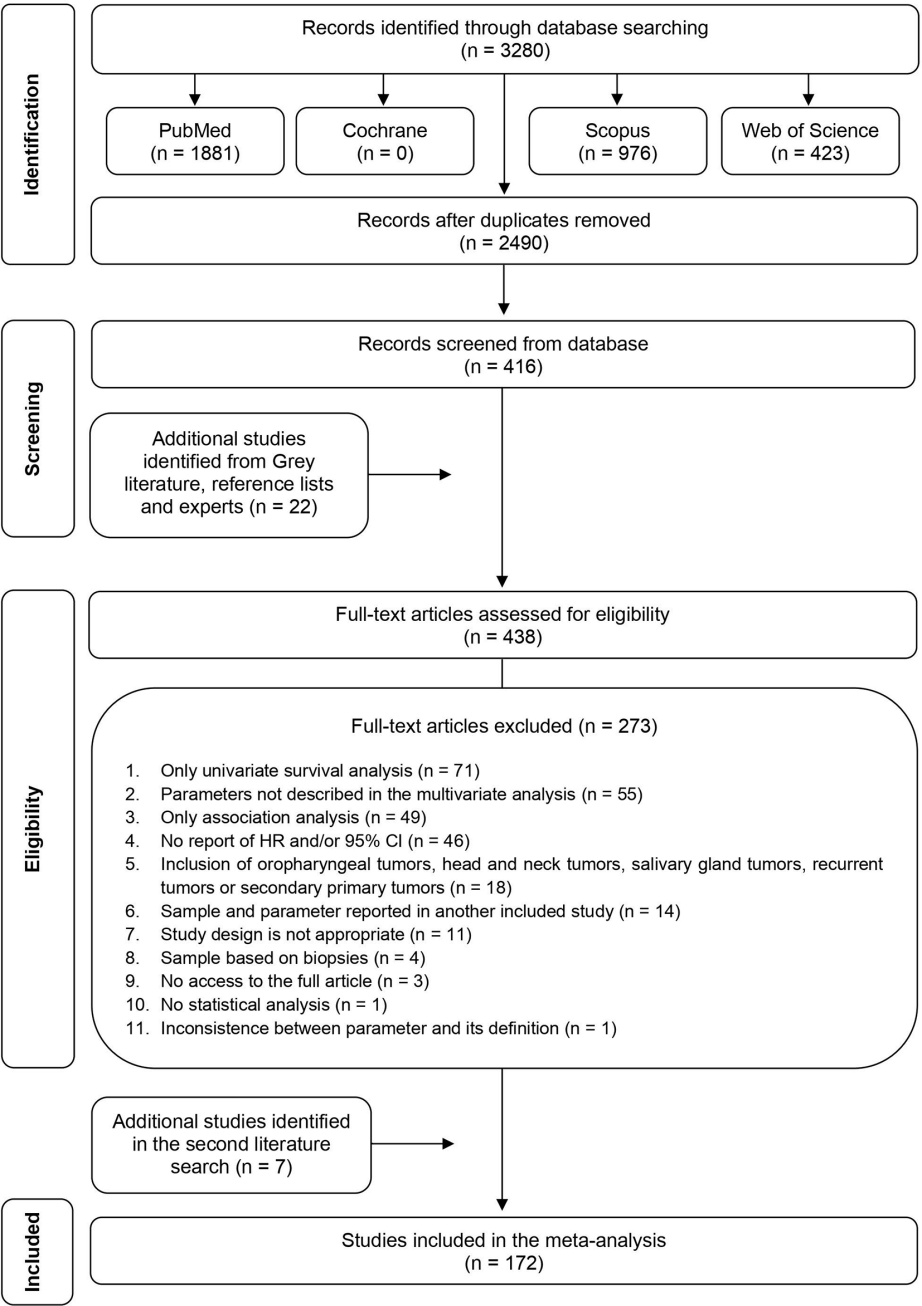
PRISMA flow diagram of literature search and selection criteria applied in this study.

The search strategy yield 3,280 articles (**Figure 1**). Following duplicate removal, 2,490 studies were included in the title and abstract screening, and of these, 2,074 studies were excluded, leaving 416 studies meeting the criteria for full-text screening. Other 22 additional studies were identified from Grey literature, reference tracking or experts, ending with 438 studies for full-text screening. After full-text review, 273 titles were excluded and full details are depicted in **Supplementary Table S1**, resulting in 165 articles remaining for data abstraction. In the second literature search, seven new articles, published in 2021, were found and included in the study. The characteristics of 172 studies included are shown in **Supplementary Table S2**.

### Study Characteristics

The included articles were published from 1999 to 2021 and were conducted in 26 different countries (**Supplementary Table S2**). The most commons countries of publication were Taiwan, with 36 studies, and the USA, with 30 studies. Only seven (4.2%) studies were performed with samples from more than one country. The studies have explored different histological characteristics, including DOI, ENE, PNI, LVI, surgical margins, tumor thickness, bone invasion, pattern of invasion, tumor budding, and TSR. Six articles analyzed the inflammatory response, but they have applied different cutoff and systems of evaluation, not allowing any further analysis.

### Synthesis of Results

#### DOI

For evaluation of the significance of DOI on OS, samples from 27 studies, accounting to 7,324 patients, were pooled, resulting in a HR of 1.94 (95% CI: 1.54–2.44, *p* < 0.00001, *I* ^2^ = 80%) (**Table 1** and **Supplementary Figure S1A**). A similar tendency was observed for DSS (HR: 1.45, 95% CI: 1.29–1.64, *p* < 0.00001, *I* ^2^ = 20%), which counted with 11 studies spanning 7,781 patients (**Table 2** and **Supplementary Figure S1B**), and for DFS (HR: 1.53, 95% CI: 1.29–1.81, *p* < 0.00001, *I* ^2^ = 76%), calculated with 6,348 patients reported in 27 studies (**Table 3** and **Supplementary Figure S1C**). For DSS, one study (13) displayed a significant weight (~64%) on the pooled HR, but its exclusion made the association of DOI and cancer death even stronger, with a HR of 1.75 (95% CI: 1.44–2.13, *p* < 0.00001, *I* ^2^ = 0%).

**Table 1:** Effect of the selected histological features in the overall survival (OS) of patients with oral squamous cell carcinoma.

	Number of studies	Number of cases	HR (95% CI)	*p*-value	Z effect	*I* ^2^	Analysis model
Depth of invasion	27	7,324	1.94 (1.54–2.44)	<0.00001	5.65	80%	Random effect
Extranodal extension	40	48,217	2.18 (1.91–2.47)	<0.00001	11.87	60%	Random effect
Perineural invasion	33	10,045	1.66 (1.51–1.82)	<0.00001	10.51	17%	Fixed effect
Lymphovascular invasion	30	30,481	1.81 (1.55–2.13)	<0.00001	7.31	52%	Random effect
Surgical margins	31	63,470	1.56 (1.41–1.73)	<0.00001	8.43	61%	Random effect
Tumor thickness	5	1,651	1.09 (1.02–1.16)	0.01	2.55	28%	Fixed effect
Bone invasion	4	1,603	1.91 (1.02–3.57)	0.04	2.03	77%	Random effect
Pattern of invasion							
Cohesive system	4	543	2.25 (1.56–3.23)	<0.0001	4.36	0%	Fixed effect
Worst-pattern of invasion	2	420	2.40 (1.19–4.84)	0.01	2.45	0%	Fixed effect
Tumor budding	5	986	2.96 (1.36–6.45)	0.006	2.73	73	Random effect
Tumor-stroma ratio	1	226	1.69 (1.02–2.81)	0.04	–	–	–

**Table 2:** Effect of the selected histological features in the disease-specific survival (DSS) of patients with oral squamous cell carcinoma.

	Number of studies	Number of cases	HR (95% CI)	*p*-value	Z effect	*I* ^2^	Analysis model
Depth of invasion	11	7,781	1.45 (1.29–1.64)	<0.00001	6.22	20%	Fixed effect
Extranodal extension	12	7,460	1.88 (1.51–2.35)	<0.00001	5.64	51%	Random effects
Perineural invasion	26	7,523	1.63 (1.46–1.83)	<0.00001	8.52	38%	Fixed effect
Lymphovascular invasion	13	4,411	1.71 (1.46–2.01)	<0.00001	6.57	0	Fixed effect
Surgical margins	19	20,680	1.71 (1.58–1.85)	<0.00001	13.20	35%	Fixed effect
Tumor thickness	3	638	1.07 (1.01–1.13)	0.02	2.28	0	Fixed effect
Bone invasion	5	2,773	2.12 (1.69–2.66)	<0.00001	6.51	41%	Fixed effect
Pattern of invasion							
Cohesive system	4	1,229	2.63 (1.56–4.46)	0.0003	3.61	32%	Fixed effect
Worst-pattern of invasion	2	122	2.42 (1.00–5.88)	0.05	1.96	0	Fixed effect
Tumor budding	5	969	1.72 (1.35–2.18)	<0.00001	4.43	0	Fixed effect
Tumor-stroma ratio	3	724	2.26 (1.65–3.11)	<0.00001	5.03	49%	Fixed effect

**Table 3:** Effect of the selected histological features in the disease-free survival (DFS) of patients with oral squamous cell carcinoma.

	Number of studies	Number of cases	HR (95% CI)	*p*-value	Z effect	*I* ^2^	Analysis model
Depth of invasion	27	6,348	1.53 (1.29–1.81)	<0.00001	4.88	76%	Random effect
Extranodal extension	31	12,835	2.26 (1.93–2.65)	<0.00001	10.08	51%	Random effect
Perineural invasion	45	15,268	1.62 (1.49–1.76)	<0.00001	11.34	23%	Fixed effect
Lymphovascular invasion	30	8,187	1.56 (1.22–1.99)	0.0003	3.59	65%	Random effect
Surgical margins	25	15,300	2.47 (1.90–3.21)	<0.00001	6.74	76%	Random effect
Tumor thickness	4	1,556	2.22 (1.43–3.45)	0.0004	3.54	49%	Fixed effect
Bone invasion	5	2,511	1.97 (1.23–3.16)	0.005	2.80	67%	Random effect
Pattern of invasion							
Cohesive system	4	505	2.20 (1.37–3.63)	0.001	3.26	46%	Fixed effect
Worst-pattern of invasion	5	892	2.82 (2.03–3.91)	<0.00001	6.22	0%	Fixed effect
Tumor budding	5	1,142	2.02 (1.50–2.71)	<0.00001	4.64	30%	Fixed effect
Tumor-stroma ratio	4	950	2.05 (1.59–2.64)	<0.00001	5.51	0	Fixed effect

The cutoff for dichotomization of the samples was variable among the published studies. However, the most common cutoff points were 4 or 5 mm, and subgroup meta-analyses were performed. For the cutoff value of 4 mm, increased risk for poor OS (HR: 3.10, 95% CI: 1.78–5.40, *p* < 0.0001, *I* ^2^ = 0%) (**Supplementary Figure S2A**), DSS (HR: 1.82, 95% CI: 1.40–2.37, *p* < 0.00001, *I* ^2^ = 53%) (**Supplementary Figure S2B**), and DFS (HR: 2.12, 95% CI: 1.51–2.98, *p* < 0.0001, *I* ^2^ = 35%) (**Supplementary Figure S2C**) were observed. Similarly, the cutoff point of 5 mm DOI was associated with high hazards for OS (HR: 2.06, 95% CI: 1.35–3.15, *p* = 0.0008, *I* ^2^ = 78%) (**Supplementary Figure S3A**), DSS (HR: 1.64, 95% CI: 1.09–2.47, *p* = 0.02, *I* ^2^ = 0%) (**Supplementary Figure S3B**), and DFS (HR: 1.74, 95% CI: 1.37–2.23, *p* < 0.00001, *I* ^2^ = 0%) (**Supplementary Figure S3C**). For those subgroup analyses, the findings of the sensitivity analyses were not substantially different from those of the main analyses.

#### ENE

ENE was evaluated in 55 articles by histopathological examination. Meta-analysis involving 48,217 patients (40 studies) showed a HR of 2.18 (95% CI: 1.91–2.47, *p* < 0.00001) for OS detected (**Table 1** and **Supplementary Figure S4A**). A similar trend was detected for both DSS and DFS, which included 7,460 (12 studies) and 12,835 (31 studies) patients respectively in the meta-analyses. For DSS (**Table 2** and **Supplementary Figure S4B**), the HR was 1.88 (95% CI: 1.51–2.35, *p* < 0.00001) and for DFS (**Table 3** and **Supplementary Figure S4C**) was 2.26 (95% CI: 1.93–2.65, *p* < 0.00001). The heterogeneity was high (*I* ^2^ > 50%), but in all meta-analyses, the number of samples among studies did not reach great variation, and the pooled HR overlapped with the confidence intervals of most studies.

#### PNI

The literature search identified 69 articles that reported the impact of PNI on OSCC prognosis. The majority of them classified PNI as the presence of the tumor cells inside the nerve or surrounding it, and only few studies have explored other specific aspects of PNI, including the size of the involved nerve, the number of foci or its localization (intratumoral or peritumoral) (14–17). For OS, 10,045 patients from 33 studies were included in the meta-analysis (**Supplementary Figure S5A**), revealing a pooled HR of 1.66 (95% CI: 1.51–1.82, *p* < 0.00001) (**Table 1**). A significant association of PNI and DSS was also detected in the meta-analysis containing 7,523 patients from 26 studies (pooled HR: 1.63, 95% CI: 1.46–1.83, *p* < 0.00001) (**Table 2** and **Supplementary Figure S5B**). For DFS, with a sample composed of 15,268 patients from 45 studies, the meta-analysis revealed a significant association between PNI and tumor recurrence (pooled HR: 1.62, 95% CI: 1.49–1.76, *p* < 0.00001) (**Table 3** and **Supplementary Figure S5C**). Low heterogeneity was found in all meta-analyses (*I* ^2^ = 17% for OS, *I* ^2^ = 38% for DSS, *I* ^2^ = 23% for DFS).

#### LVI

Forty-seven studies evaluated the clinical impact of LVI. For OS were 30 articles with 30,481 patients, DSS were 13 articles with 4,411 patients, and for DFS were 30 articles with 8,187 patients. Only three studies have brought a clear definition of LVI, with two of them considering the presence of tumor cells inside lumen of peripheral blood or lymphatic vessels (18, 19) and the third study considering both presence of tumor nests either within or adjacent to the endothelial cell lining (20). The presence of LVI negatively impacted OS (HR: 1.81, 95% CI: 1.55–2.13, *p* < 0.00001, *I* ^2^ = 52%) (**Table 1** and **Supplementary Figure S6A**), DSS (HR: 1.71, 95% CI: 1.46-2.01, *p* < 0.00001, *I* ^2^ = 0%) (**Table 2** and **Supplementary Figure S6B**), and DFS (HR: 1.56, 95% CI: 1.22–1.99, *p* = 0.0003, *I* ^2^ = 65%) (**Table 3** and **Supplementary Figure S6C**). Although both OS and DFS meta-analyses displayed high heterogeneity (*I* ^2^ > 50%), the exclusion of studies with high weight in the estimation of the pooled HR did not alter the statistical significance of association.

### Surgical Margins

There were 50 eligible studies for pooled analyses of surgical margin, counting 63,470 patients for OS (31 articles), 20,680 for DSS (19 articles), and 15,300 for DFS (25 articles). The cutoff to define a clear margin was variable, but most studies considered the distance of morphologically 5 mm of normal tissue. In fact, most studies compared clear margin (>5 mm of normal tissue) against the combination of close (1–4 mm of normal tissue) and involved (<1 mm of normal tissue) margins. A large number of studies (*n* = 20, 39.2%) did not define the cutoff value, dividing the tumors in clear margin or close/involved margin. Our pooled analyses revealed that an involved resection margin, regardless the distance of clear tissue, was significantly associated with worse OS (HR: 1.56, 95% CI: 1.41–1.73, *p* < 0.00001, *I* ^2^ = 61%) (**Table 1** and **Supplementary Figure S7A**), DSS (HR: 1.71, 95% CI: 1.58–1.85, *p* < 0.00001, *I* ^2^ = 35%) (**Table 2** and **Supplementary Figure S7B**), and DFS (HR: 2.47, 95% CI: 1.90–3.21, *p* < 0.00001, *I* ^2^ = 76%) (**Table 3** and **Supplementary Figure S7C**). Three studies (21–23) represented more than 30% of the OS sample, but their exclusions did not affect the statistically significant association between margin and OS. For DFS, which also showed high heterogeneity, none of the included studies showed a large number of cases, and the sequential exclusion of each study did not affect the results.

Subgroup analyses with studies applying the cutoff value of 5 mm to split into two groups (clear, ≥5 mm and close/involved, <5 mm) revealed an increased risk for OS (HR: 1.53, 95% CI: 1.16–2.03, *p* = 0.003, *I* ^2^ = 68%), which was based on 10 articles and 9,659 patients (**Supplementary Figure S8A**), for DSS (HR: 1.68, 95% CI: 1.19–2.35, *p* = 0.003, *I* ^2^ = 76%), based on eight articles and 6,156 patients (**Supplementary Figure S8B**), and for DFS (HR: 2.61, 95% CI: 2.10–3.24, *p* < 0.00001, *I* ^2^ = 75%), based on nine articles and 2,483 patients (**Supplementary Figure S8C**).

### Tumor Thickness

Ten studies were included, with five of them in a total of 1,651 samples reporting tumor thickness influence on OS (**Table 1**), three with 638 samples on DSS (**Table 2**), and four with a total of 1,556 samples on DFS (**Table 3**). As there was no uniform cutoff for tumor thickness among the studies, we have assumed the dichotomization of studies in low and high values. Although significant, the risk of high tumor thickness on OS (HR: 1.09, 95% CI: 1.02–1.16, *p* = 0.01, *I* ^2^ = 28%) (**Supplementary Figure S9A**) and DSS (HR: 1.07, 95% CI: 1.01–1.13, *p* = 0.02, *I* ^2^ = 0%) (**Supplementary Figure S9B**) was modest, whereas it was stronger in DFS (HR: 2.22, 95% CI: 1.43–3.45, *p* = 0.0004, *I* ^2^ = 49%) (**Supplementary Figure S9C**).

### Bone Invasion

The risk of bone invasion was reported in eight studies. As only three of these differentiated between cortical or medullary invasion (24–26), we divided the samples in presence vs. absence of bone invasion. The pooled HR indicated that patients with bone invasion were at higher risk for OS (HR: 1.91, 95% CI: 1.02–3.57, *p* = 0.04, *I* ^2^ = 77%), which was based on four studies (1,603 samples) (**Table 1** and **Supplementary Figure S10A**), DSS (HR: 2.12, 95% CI: 1.69–2.66, *p* < 0.00001, *I* ^2^ = 41%), accounting to 2,773 patients spanned in five studies (**Table 2** and **Supplementary Figure S10B**), and DFS (HR: 1.97, 95% CI: 1.23–3.16, *p* = 0.005, *I* ^2^ = 67%), with samples from five studies (2,511 patients) (**Table 3** and **Supplementary Figure S10C**). Although the heterogeneity among studies was high, the exclusion of study with the highest weight in the estimation of the pooled HR for OS did not alter the significance of association, whereas for DFS, the exclusion made the association even more significant (HR: 2.06, 95% CI: 1.01–4.18, *p* = 0.0001, *I* ^2^ = 0%).

### Pattern of Invasion

Altogether, 27 studies investigated the pattern of invasion impact on OSCC survival, but they have applied different systems of classification. One study has applied the mode of invasion described by Jakobsson et al. (27), six studies have applied the system proposed by Yamamoto et al. (28), three studies have applied the pattern of invasion of Bryne’s grading system (29), six studies have classified the pattern of invasion in cohesive and noncohesive, according to Bryne’s grading system, and 11 studies have utilized the worst-pattern of invasion (WPOI).

The Yamamoto’s system represents the mode of invasion proposed by Jakobsson et al. (27), with a small modification. In this system, grade 4 was subcategorized in two, grades 4C and 4D. In 4C, the invasive cells are found in cord-like structures, whereas in 4D, the invasive cells are located deeply as single cells or in nests with few cells (there is no indication of cell number in this classification). As the studies have applied quite different ways of grouping, the number of studies was insufficient for a meta-analysis.

The classification of pattern of invasion in cohesive and noncohesive is based on Bryne’s grading system. In the cohesive mode, the invasive cells are distributed in sheets or strands with >15 cells, and the noncohesive mode is composed of narrow strands or noncohesive small groups (<15 cells) or single cells. Although two studies have applied the traditional pattern of invasion of the Bryne’s grading system (30, 31), which is based on four grades, they have grouped the samples in the way that the tumors ended up classified as cohesive and noncohesive. Thus, eight studies were included in the meta-analysis to evaluate the impact of the cohesive/noncohesive pattern of invasion on OSCC survival. The pooled HR for OS, which was based on four studies with 543 samples, was 2.25 (95% CI: 1.56–3.23, *p* < 0.0001, *I* ^2^ = 0%) (**Table 1** and **Supplementary Figure S11A**), for DSS, which was also based on four studies but 1,229 samples, was 2.63 (95% CI: 1.56–4.46, *p* = 0.0003, *I* ^2^ = 32%) (**Table 2** and **Supplementary Figure S11B**), and for DFS was 2.20 (95% CI: 1.37–3.63, *p* = 0.001, *I* ^2^ = 46%) (**Table 3** and **Supplementary Figure S11C**), which counted with 505 samples from four studies.

After modification introduced by Brandwein-Gensler et al. (32), WPOI has five grades and the studies identified in our literature search have applied different criteria to dichotomize those grades. However, seven studies have investigated the influence of WPOI dividing the tumors in two groups, low (grouping grades 1, 2, and 3) and high (grouping grades 4 and 5), and a meta-analysis was performed. The pooled HRs for WPOI revealed a poor OS (HR: 2.40, 95% CI: 1.19–4.84, *p* = 0.01, *I* ^2^ = 0%) (**Table 1** and **Supplementary Figure S12A**), DSS (HR: 2.42, 95% CI: 1.00–5.88, *p* = 0.05, *I* ^2^ = 0%) (**Table 2**; **Supplementary Figure S12B**), and DFS (HR: 2.82, 95% CI: 2.03–3.91, *p* < 0.00001, *I* ^2^ = 0%) (**Table 3** and **Supplementary Figure S12C**).

### Tumor Budding

Thirteen studies fulfilled the inclusion criteria for tumor budding, with five studies (986 samples) for OS (**Table 1**), five studies (969 samples) for DSS (**Table 2**), and five studies (1,142 samples) for DFS (**Table 3**). The majority of the studies considered the cutoff value of five buds per field (<5 buds or 5≥ buds). The presence of high bud activity was highly associated with poor OS (HR: 2.96, 95% CI: 1.36–6.45, *p* = 0.006, *I* ^2^ = 73%) (**Supplementary Figure S13A**), DSS (HR: 1.72, 95% CI: 1.35–2.18, *p* < 0.00001, *I* ^2^ = 0%) (**Supplementary Figure S13B**) and DFS (HR: 2.02, 95% CI: 1.50–2.71, *p* < 0.00001, *I* ^2^ = 30%) (**Supplementary Figure S13C**). The heterogeneity was significantly high only for OS, and the exclusion of study of Xu et al. (33) changed notably the results (HR: 4.14, 95% CI: 2.55–6.72, *p* < 0.00001, *I* ^2^ = 0%).

### TSR

We identified four studies that reported the impact of the TSR on OSCC survival, and all applied the same cutoff. Only one study verified TSR on OS (**Table 1**), while three studies investigated the association with DSS (724 patients) (**Table 2**) and four studies with DFS (950 patients) (**Table 3**). The pooled HR revealed a clear negative influence of TSR on both DSS (HR: 2.26, 95% CI: 1.65–3.11, *p* < 0.0001, *I* ^2^ = 49%) (**Supplementary Figure S14A**) and DFS (HR: 2.05, 95% CI: 1.59–2.64, *p* < 0.0001, *I* ^2^ = 0%) (**Supplementary Figure S14B**).

### Risk of Bias Within Studies

The overall risk of bias of each study is depicted in **Supplementary Table S3**. Overall agreement on methodological quality scores between the reviewers was 87.1%, with mainly disagreement involving the prognostic factor measurement and the determination of study confounding parameters. Cases in disagreement were set up after discussion of evaluators. We ended up with 26 (15.1%) studies classified with low risk of bias, 92 (53.5%) with moderate risk, and 54 (31.4%) with high risk. The main sources of bias were associated with rating the study attrition, the measurement of prognostic factors, the outcomes, and the statistical analysis. In the study attrition, where the period of follow-up is taken into consideration, several articles have performed their conclusions with a follow-up of less than 5 years after diagnosis/treatment, compromising the quality of evidence. A clear definition of the parameter, including cutoff applied to classification, is important under the domain prognostic factor measurement, and in several studies, the parameters were not clearly defined. In many studies, for example, the authors divided the samples based on clear or involved margins, without a clear definition of the distance applied to define a clear margin. Moreover, in this domain, it was considered if the analysis was performed for more than one observer, increasing consistency in the classification. Most articles did not report it or it was performed by only one observer. Although survival outcomes are well known, lack of a clear definition was detected in 40 studies. Furthermore, in many articles analyzing relapse (DFS), there was no description of whether the cases of recurrence were histologically confirmed. Ninety studies were classified as high risk of bias in the studying confounding domain, because the studies did not include important confounding variables in the model or included recurrence as a parameter in the model, not as an endpoint for DFS. The number and choice of adjustment factors varied across studies, but if the study failed to include clinical stage (or the TNM parameters individually) and treatment in the model, it was directly classified as high risk of bias in this domain. The strategy of the model building was not appropriately described in 23 articles, and as this is a crucial question for the quality of results, hence these articles were directly downgraded in the domain and classified as high risk of bias.

### Quality of Evidence

The GRADE methodology was used to assess the quality of evidence for each prognostic parameter. If the studies at high and low risk of bias provided similar estimates of association and the weight of studies with high risk of bias contributed with a small proportion of the pooled HR, we did not rate down for risk of bias during judgment. The risk of bias was also assessed using the funnel plots (data not shown). If the visual inspection demonstrated that studies with small cohorts and without important effects appeared to be missing for the analysis, we did not rate down the level of evidence. For inconsistency assessment, *I* ^2^ values were taken into consideration, but the pooled HRs and the 95% CI of the individual studies in relation to decision thresholds were also carefully inspected. When the pooled HR overlapped with the confidence intervals of most studies, we did not downgrade the evidence for this reason. However, when different scales were used to pool across studies (or the majority of studies did not clearly define the cutoff point), we rated down the level of evidence. Although the number of patients varied among the studies, the population and the outcomes of interest were fully similar, bringing directness to the quality of evidence. Not serious evidence of indirectness was detected in the study. The quality of evidence was rated down for imprecision if the pooled sample size was small (<1,000 samples) or if the pooled confidence intervals did not exclude an HR of 1.0 by a considerable margin.

The quality of evidence varied from very low to high because we found some situations of serious risk of bias, inconsistency, and imprecision (**Supplementary Table S4**). The certainty for DOI was low in general, because we have grouped a large number of articles that displayed baseline confounding bias and applied different scales/cutoff. However, when we grouped only articles with specific cutoff (4 or 5 mm), the certainty of evidence was high for DFS, albeit the number of samples dropped. A quite similar situation was observed for surgical margins. In general, the level of evidence was low, because different scales were used to pool across studies, but using studies with a cutoff value of 5 mm, the evidence was improved. For the classical ENE and PNI, the evidence ranged from moderate to high, whereas LVI showed levels of evidence between low and high. The certainty of evidence for tumor thickness, bone invasion, cohesive pattern of invasion, WPOI, tumor budding, and TSR was mainly low or very low, because of serious risk of bias and inconsistency. For many of these parameters, the sample size was less than 1,000 and then we downgraded for imprecision.

## Discussion

An ideal biomarker should be specific, measurable, meaningful, and preferentially of easy access and low cost. Although this field is rapidly developing and has become a routine practice for some tumors, the OSCC therapeutic approach and prognosis are still based on TNM clinical staging. Unfortunately, the TNM system is nonspecific and yields inconsistent information regarding disease biology. New biomarkers that complement the TNM system, predicting a proper response to treatment and prognosis, are therefore essential. This review explored the impact of histopathological features diagnosed in HE-stained slides as prognostic markers for OSCC patients. The advanced progression in genomics has highlighted many mutations and molecular events related to cancer, allowing the identification of several cancer biomarkers. However, characteristics identified in HE-stained slides hold many of the ideal features for a biomarker. HE staining is a highly standardized and universal procedure, of low cost, that allows a uniform extraction of information.

In this systematic review, 11 histopathological features of OSCC associating with patient’s outcomes were identified from published studies during the last two decades (1999 to 2021). Many of these features have been examined in several studies, allowing robust meta-analyses, whereas others were only examined in a limited manner. Moreover, several relevant studies were published more than 20 years ago, revealing the long-time interest in determining the impact of histological features on survival of OSCC patients. The results of our current meta-analyses confirmed that traditional histopathological factors such as DOI, ENE, PNI, LVI, and involvement of the surgical margins are significantly associated with shortened OS, DSS, and DFS in patients with OSCC. Although the certainty of evidence displayed significant variation due to situations of risk of bias, inconsistency, and imprecision, these results were extracted from a large number of eligible studies with thousands of patients, reducing bias of individual studies and bringing magnitude for the conclusion. Moreover, promising associations of tumor thickness, bone invasion, pattern of invasion, tumor budding, and TSR with poor outcomes were also detected, but the size of the combined samples was relatively small, guaranteeing further research.

One of the most solid evidence of association of DOI with OSCC outcome was reported by Ebrahimi and collaborators (13), after an international collaborative study with 3,149 samples from cancer centers in Italy, Brazil, Israel, Australia, Germany, USA, Taiwan, and India. To our knowledge, only one meta-analysis has been previously published on DOI in OSCC, and this study identified high odds for lymph node metastasis at diagnosis and recurrence in tumors with high DOI, regardless of the cutoff point (34). The major difference between this previous meta-analysis and the current study is the higher number of included articles in the current study, the inclusion of HRs derived from multivariate analyses and subgroup meta-analyses for different cutoff points. Although the DOI cutoff point varied among studies, most studies have applied either 4 or 5 mm. The subgroup analyses revealed that both cutoffs were significantly associated with low rates of OS, DSS, and DFS, though the HR values were higher, with less heterogeneity and the pooled lower confidence intervals excluded an HR of 1.0 by higher margins for the cutoff of 4 mm. In a recent study, Shinn et al. (35) showed that the risk of regional recurrence in oral tongue carcinomas begins to increase progressively with any DOI.

Even with clear definitions, DOI and tumor thickness are commonly used interchangeably (36, 37). The main explanation for this fact is that oral cancers present commonly as an ulceration, and the inclusion or not of few epithelial layers into measurement adds minimal depth, which is unlikely to be clinically significant (38). In this meta-analysis, we have separated tumor thickness and DOI according to the definition of the articles, and our results showed a similar pooled HR only for DFS, suggesting superiority of DOI in discriminate tumors with worse OS and DSS. Nevertheless, the number of samples that analyzed tumor thickness was lower than those that analyzed DOI. In a retrospective study, Dirven et al. (36) found that the both T category and the TNM stage prognostic performance of the 8th TNM staging of OSCC are similar regardless of whether DOI or tumor thickness were used as the T category modifier, whereas the study by Liu et al. (37) revealed that DOI and tumor thickness were similar regarding their association with nodal metastasis, but the cutoff values were quite different (4.5 mm for DOI and 8 mm for TT). In terms of DOI and the 8th TNM staging of OSCC, Almangush et al. (39) have demonstrated a better prognostic discrimination for early oral tongue carcinomas lowering the cutoff to 2 mm for T1 and to 4 mm for T2. Altogether, the interchangeable possibility between DOI and tumor thickness and the ideal DOI cutoff to upstage the T category represent gaps that deserve further research. Interestingly, the method of measurement is not often defined within the literature.

The presence of ENE in OSCC is associated with poor survival and increased risk for regional recurrence and distant disease (40) and its presence is an important factor taken into consideration for postoperative radiotherapy or chemoradiotherapy (41). With a large number of samples among the relevant studies, the results of this meta-analysis confirmed that the presence of ENE increased significantly hazards for mortality and relapse. The overall quality of evidence was considered good, suggesting it as a true estimated effect. We have pooled only articles comparing presence vs. absence of ENE, following the recommendation of the current TNM classification. However, few studies have attempted to determine clinically relevant qualitative and quantitative characteristics of ENE, including number of metastatic nodes with ENE, size of the metastatic focus and distance from the edge of the nodal capsule to the metastatic tumor edge (42–44). Although our study has not analyzed lymph node ratio (LNR), also called lymph node burden, recent systematic reviews have demonstrated its prognostic impact for OSCC (45, 46).

As recently revised by Abdel-Halim et al. (47), different definitions of ENE are applied in the literature, which can explain the significant variations in the reported ENE incidence, but all definitions refer to the extension of tumor outside the capsule of a lymph node and into the perinodal soft tissue. Indeed, several studies included in this meta-analysis did not clearly define ENE. Traditionally, ENE is limited to the perinodal soft tissue, making the clinical diagnosis somehow difficult. In a recent meta-analysis, the sensibility and sensitivity of diagnostic accuracy of image systems, including computed tomography, magnetic resonance imaging and positron emission tomography with computed tomography, varied between 72% and 80% and 77% and 83%, respectively, in relation to histopathological examination, the gold standard tool for ENE diagnosis (48). Given the widespread clinical use of ENE, particularly after its incorporation in the TNM grading system, clear definitions, classification systems, and guides to avoid interobserver variations are currently needed.

PNI has been reported to be associated with treatment decisions and outcome in many types of cancer, including OSCC, but the findings are not completely unanimous (49, 50). This controversy may be in part due to the application of different criteria among studies, resulting in reported PNI detection varying from 5.2% to 90% of patients with OSCC (51). Although the definition proposed by Liebig and collaborators (52) is the most frequently applied, where PNI is classified as the presence of tumor cells inside nerve sheaths or surrounding at least 1/3 of nerve circumference, many studies call PNI when cells are only touching a portion of nerve (10). As expected, the interobserver agreement for the diagnostic of PNI was better applying the criteria of tumor cells invading the nervus sheaths compared with the PNI definition was set as tumor surrounding a nerve, which can involve subjectivity (53). In such contextual disagreement, a meta-analysis is important to settle controversies arising from conflicting results due to its ability to disregard bias posed by individual studies. With a moderate to high quality of evidence, the results of this meta-analysis showed that the presence of PNI was strongly associated with increased risk of mortality and recurrence, confirming two previous meta-analyses on PNI (54, 55). Although there are some overlap among the meta-analyses, the first included head and neck squamous cell carcinomas (54) and the second included only oral tongue carcinomas (55), whereas we performed our meta-analysis with studies that reported a Cox multivariate-adjusted HR in oral cavity cancers, which brings refinement for the conclusion. Therefore, studies on PNI should focus on formulating a clear definition, considering an objective and more reproducible criteria for histopathologic assessment. Moreover, further studies should confirm the promising results associated with quantitative and qualitative features of PNI, including size of the involved nerve, number of foci, and intratumoral or peritumoral localization (15–17). It is also important to confirm the importance of PNI in treatment decision making, since recent reviews have not confirmed improved survival rates with adjuvant postoperative therapy for patients with PNI (49, 56).

As an essential step in tumor metastasis, LVI has been considered a prognostic marker for poor prognosis in patients with OSCC (57). In the current meta-analysis of 30 studies for OS (30,481 samples), 30 studies for DFS (8,187 samples), and 13 for DSS (4,411 samples), we confirmed the significant prognostic role of LVI for OSCC. However, the level of evidence was low for both OS and DFS because of the large heterogeneity in the reported HRs. Another important issue is regarding the definition of LVI. Only three included studies have provided LVI definition, with Fives et al. (18) and Mascitti et al. (19) defining LVI as presence of tumor cells within the vascular space and Liu et al. (20) classifying LVI as tumor cells both within or adjacent to the vessels. Moreover, most studies have followed the tendency of considering lymphatic or vascular together under the concept of LVI, but some studies have classified the invasion separately as venous or lymphatic invasion. Some studies have suggested that the prognostic value of LVI is meaningless in OSCC (58, 59). Explanations for this negative association may be due to heterogeneity of OSCC biology or difficulties in identifying LVI in HE-stained sections. To avoid the later problem, immunohistochemistry has been advocated, but a recent study demonstrated little benefits of immunohistochemical analysis for CD31 and D2-40 on identification of LVI on histologically negative cases of tongue carcinomas (60). These knowledge gaps present areas for further research. Even with these difficulties, the results of this meta-analysis support the clinical significance of LVI as a marker for high-risk mortality and recurrence.

Achieving negative resection margins is the main goal in surgical oncology, because a clear margin is associated with reduced risk of recurrence and long period of survival in OSCC (61). In contrast, a positive margin or a margin with an inadequate distance of normal tissue (the so called close margin) has negative prognostic implications and adjuvant treatment is recommended (62). Our findings show a strong association between positive margin and risk of overall mortality, disease-specific mortality, and recurrence. Regrettably, the certainty of evidence for these outcomes is questionable based on very serious risks of bias and inconsistency among included studies due to heterogeneous results, application of different scales or lack of a clear definition of cutoff. However, in the subgroup analysis with studies that applied the cutoff value of at least 5 mm of normal tissue between the most invasive cancer cells and the outer edge of the removed tissue, the certainty of negative impact of positive margin on overall mortality (HR: 1.53), cancer-specific mortality (HR: 1.68), and recurrence (HR: 2.61) was improved. The association of involved margin with increased overall and disease-specific mortality is in line with the meta-analysis performed by Hamman et al. (63), who reported a higher OS for clear margins after data extraction from nine studies, and with the meta-analyses of Anderson et al. (64), which was based on four studies, and Bulbul et al. (65), based on eight studies, who demonstrated higher likelihood of local recurrence for a positive margin. The results of our current meta-analysis, which is the largest and the most comprehensive one to date, in combination with those previous ones underscore the impact of a positive margin on prognostic of OSCC, supporting current recommendations of adjuvant postoperative chemoradiation therapy for cases with involved margins. However, further studies should follow restrict recommendations to define the exact cutoff applied to a negative margin.

OSCC cells may invade the adjacent bones, particularly the mandible, and such invasion worsens prognosis and requires more complex therapeutic regimens (25). Bone invasion can assume two typical patterns—a cortical pattern, when it is superficial, limited to the cortex, or a medullary pattern, when tumor cells are found inside the bone, in small clusters or finger-like structures (66). To the best of our knowledge, no previous meta-analysis has explored the impact of bone invasion on OSCC prognosis. Only eight studies fulfilled the inclusion criteria, and most of them have verified the effect of bone invasion/infiltration on OSCC outcome, regardless of the pattern. Although the pooled HR for all three outcomes reached a significant level, the number of samples was limited and the heterogeneity among the studies produced a very low level of evidence. Ash et al. (23) and more recently Mücke et al. (67) and Petrovik et al. (68) have concluded that the prognosis is not worsened by bone invasion, and no postoperative treatment should be guided by this histological feature in adequately resected tumor. In line with this observation, two studies have demonstrated that presence of cortical invasion does not affect survival rates, but medullary bone invasion is an independent predictor of recurrence and poor prognosis (25, 26). Even though the findings of this meta-analysis support the impact of bone invasion on OSCC prognosis, the results are limited by the small number of studies with clear heterogeneity. Further studies with large number of samples, able to perform analysis separating cortical and medullary invasion, are needed to confirm the conflicting data found in the literature.

Tumor cells may invade neighboring host tissues in many different patterns, which have been explored regarding their prognostic impact. The most common patterns found in the literature search, that allowed a systematic combination of patterns for meta-analysis, were the cohesive and noncohesive classification with eight studies and the WPOI with seven studies. Although those patterns were initially incorporated into grading systems, both have shown promising prognostic results when applied individually. In this study, we demonstrated that both patterns are histopathological criteria of poor prognosis in OSCC, although with low certainty of evidence due mainly to the small number of pooled samples. Of note, both patterns take into consideration the degree of keratinocyte dyscohesion in the advancing invasive front of tumor (presence of tumor clusters composed of ≤15 tumor cells) (32, 69). Tumor budding, defined as the presence of isolated single cells or small tumor islands composed of less than five cells in the stroma of the invasive tumor (70), corresponds to the most noncohesive pattern of invasion. In fact, studies have shown that tumor budding is composed of cells exhibiting typical features of epithelial mesenchymal transition, with increasing invasiveness (71). Across multiple studies, increased tumor budding density is associated with histological criteria of poor OSCC outcomes (72–74). In our meta-analyses, tumor budding was an independent prognostic factor of OS, DSS, and DFS. Taking together that the number of samples to reach this conclusion was relatively low and we have pooled studies that applied different criteria to determine tumor budding density, the quality of evidence is low, requiring more studies with similar criteria to consolidate the clinical relevance of tumor budding. Of note, two previous meta-analyses have also confirmed the importance of tumor budding on clinical outcomes in OSCC (75, 76).

The many cellular and noncellular components of the tumor microenvironment (TME) can induce all the essential hallmarks of cancer, contributing to growth, progression, and treatment response of tumors (77). At a histological level, TSR evaluates the proportion of tumor cells to stroma at the invasive tumor front, and low TSR (stroma-rich) has been demonstrated to be a novel and practical prognostic predictor in many neoplasms, including OSCC (78–80). The results of current meta-analyses with four eligible studies confirmed that stroma-rich (low TSR) was significantly associated with poorer DSS and high risk for recurrence. Concerning the quality of evidence, it was low because of the limited number of samples to generate the pooled HR, and the risk of bias among the included studies because the subjectivity behind TSR cannot be measured, though the studies have used the same methods and cutoff to assess TSR. The findings of this meta-analysis are in line with the recent review of TSR in head and neck tumors (81). As published by Dourado et al. (74), the worse outcome associated to the fibrotic stroma is probably related to the interactions between tumor cells and cancer-associated fibroblasts (CAFs). These cells are able to produce large amounts of collagen and other extracellular matrix proteins that may control tumor cell proliferation, survival, migration, and invasion and also affect angiogenesis and immune function in the tumor microenvironment. Further studies are recommended to validate those promising findings and to define the mechanisms behind stroma-rich influences on OSCC cells. Moreover, it is important to define more objective methods to evaluate TSR.

Some limitations of our findings have already been discussed, but others, in a more general context, deserve further attention. Several of the included studies did not focus on the histological features as the primary parameter of study. Therefore, these studies may not be powered to detect changes in OSCC outcomes. However, by pooling the data across the studies that met the inclusion criteria, our analyses were sufficiently powered to detect significant associations of several histological parameters in all outcomes. The diversity on the classification criteria of some parameters and the different cutoff applied in the included studies could be regarded as limitations. To minimize this, subgroup analyses according to more specific features were performed when possible. Given our research question is of prognostic nature, we included only observational studies, and we expectedly did not identify any randomized controlled trial. Longitudinal studies are always of interest. The selection of studies with multivariate analysis to identify the independent risk factors is one of the strengths of our meta-analysis. However, in many of the primary studies, there was no description of the variables included in the multivariate model or it was clear that classical factors that may influence the prognosis of OSCC were not accounted in the analysis. Therefore, our results may be sensitive to confounding variables.

In this study, we present a comprehensive overview of the published literature pertaining to the prognostic impact of histopathological features on OSCC. Our results confirm the prognostic potential of DOI, ENE, PNI, LVI, and involvement of the surgical margins as prognostic markers and bring promising results for the association with bone invasion, tumor thickness, pattern of invasion, tumor budding, and TSR. We have also identified critical knowledge gaps that will help direct future research activity, and the priorities include clear definition of the parameters and determination of the best cutoff values, improvement in assessment of patient-related covariates that can influence outcome, and adequate strategies for building the statistical model. Studies with large cohorts from multicenter samples, integrating multiple clinical and histopathological markers in a model will definitely have more clinical application.

## Data Availability Statement

The original contributions presented in the study are included in the article/**Supplementary Material**. Further inquiries can be directed to the corresponding author.

## Author Contributions

All authors have made a substantial, direct, and intellectual contribution to the work and approved it for publication.

## Funding

This work was supported by grants from the Fundação de Amparo à Pesquisa do Estado de São Paulo (FAPESP) (2018/16077-6) and the Conselho Nacional de Desenvolvimento Científico e Tecnológico (CNPq) (407814/2018-3 and 303589/2019-1). MD was research fellow supported by FAPESP (2017/26764-8).

## Conflict of Interest

The authors declare that the research was conducted in the absence of any commercial or financial relationships that could be construed as a potential conflict of interest.

## Publisher’s Note

All claims expressed in this article are solely those of the authors and do not necessarily represent those of their affiliated organizations, or those of the publisher, the editors and the reviewers. Any product that may be evaluated in this article, or claim that may be made by its manufacturer, is not guaranteed or endorsed by the publisher.
