# Supplementary material for: The Impact of Histopathological Features on the Prognosis of Oral Squamous Cell Carcinoma: A Comprehensive Review and Meta-Analysis
Source: Front Oncol. 2021 Nov 10;11:784924. doi: 10.3389/fonc.2021.784924 (PMC8631280; doi:10.3389/fonc.2021.784924)
Supplement: Supplementary file 1 [file DataSheet_1.zip › Supplementary Figures.DOCX]

Supplementary Figure 1. Impact of the depth of the invasion (DOI), regardless of the cut-off value, on survival of patients with oral squamous cell carcinoma (OSCC).


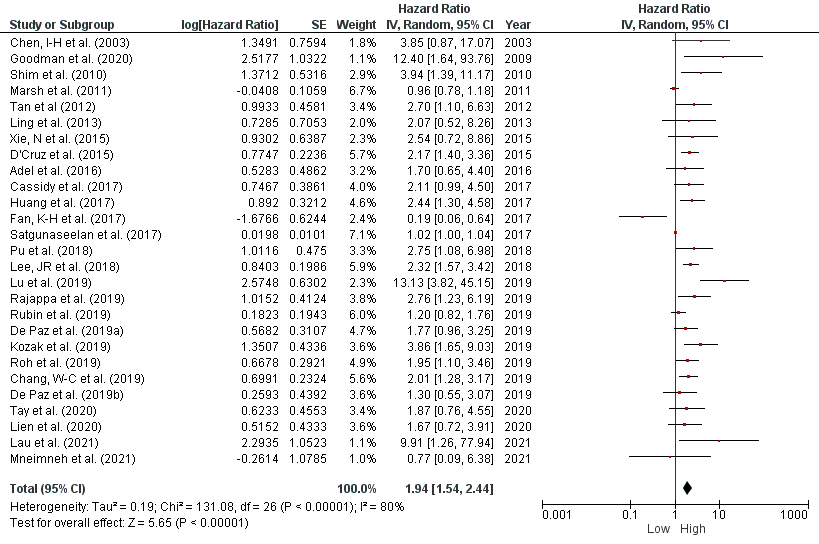


A. Forest plot of hazard ratio for overall survival (OS) comparing patients with high DOI compared with those with low DOI.


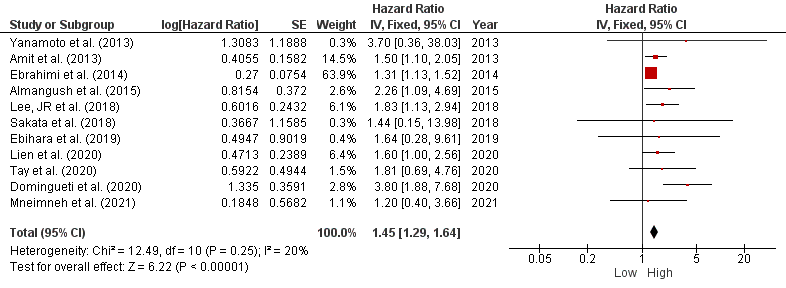


B. Forest plot of hazard ratio for disease-specific survival (DSS) comparing patients with high DOI compared with those with low DOI.


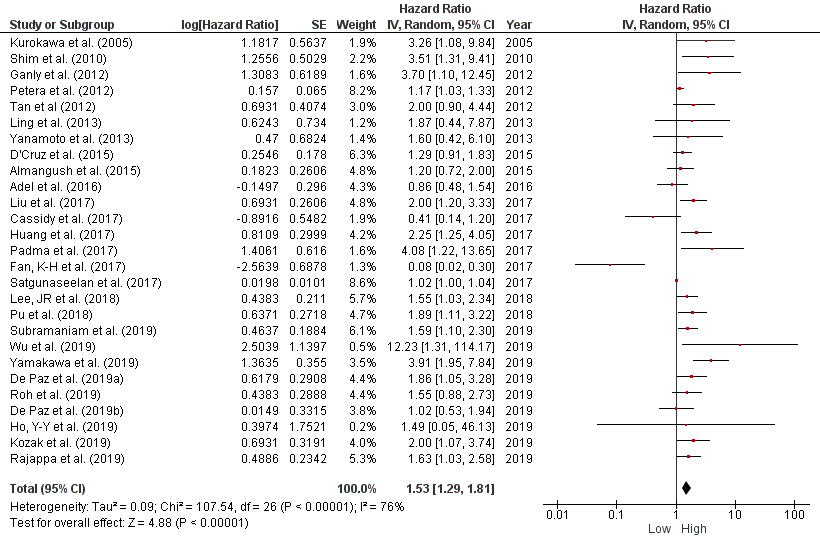


C. Forest plot of hazard ratio for disease-free survival comparing patients with high DOI compared with those with low DOI.

Supplementary Figure 2. Subgroup analysis of impact of the depth of invasion (DOI) with studies applying the cut-off value of 4 mm.


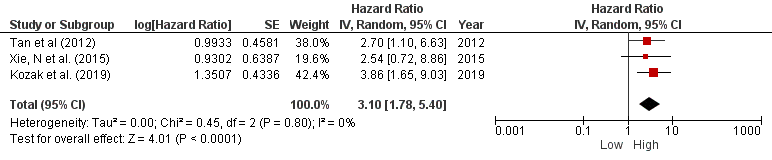


A. Subgroup analysis of impact of DOI set at 4 mm on overall survival (OS). Tumors with >4 mm were classified as high DOI compared to those with <4 mm classified as low DOI.


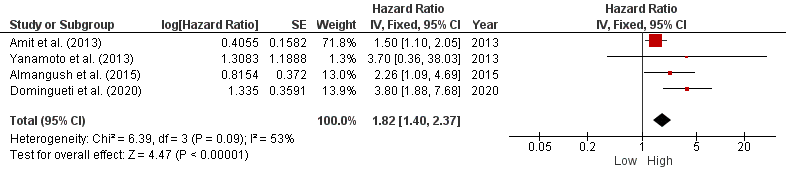


B. Subgroup analysis of impact of DOI set at 4 mm on disease-specific survival (DSS). Tumors with >4 mm were classified as high DOI compared to those with <4 mm classified as low DOI.


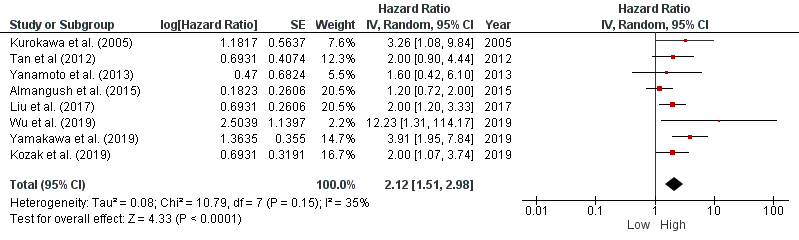


C. Subgroup analysis of impact of DOI set at 4 mm on disease-free survival (DFS). Tumors with >4 mm were classified as high DOI compared to those with <4 mm classified as low DOI.

Supplementary Figure 3. Subgroup analysis of impact of the depth of invasion (DOI) with studies applying the cut-off value of 5 mm.


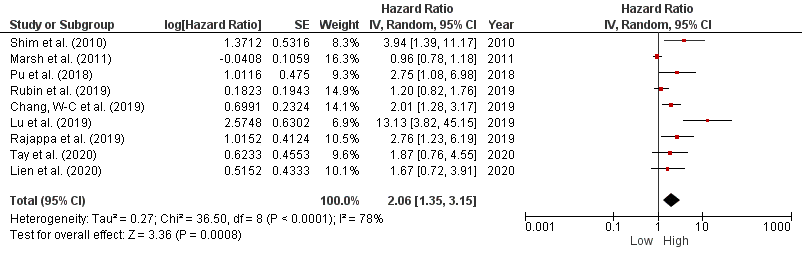


A. Subgroup analysis of impact of DOI set at 5 mm on overall survival (OS). Tumors with >5 mm were classified as high DOI compared to those with <5 mm classified as low DOI.


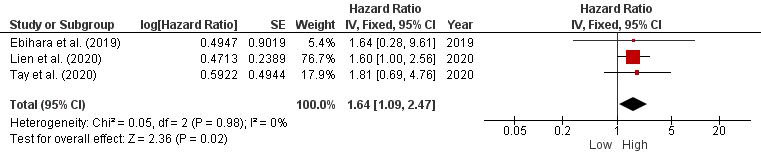


B. Subgroup analysis of impact of DOI set at 5 mm on disease-specific survival (DSS). Tumors with >5 mm were classified as high DOI compared to those with <5 mm classified as low DOI.


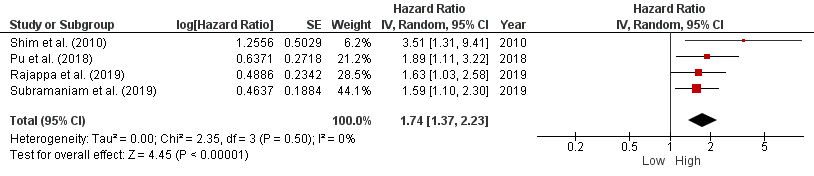


C. Subgroup analysis of impact of DOI set at 5 mm on disease-free survival (DFS). Tumors with >5 mm were classified as high DOI compared to those with <5 mm classified as low DOI.

Supplementary Figure 4. Impact of the extranodal extension (ENE) on survival of patients with oral squamous cell carcinoma (OSCC).


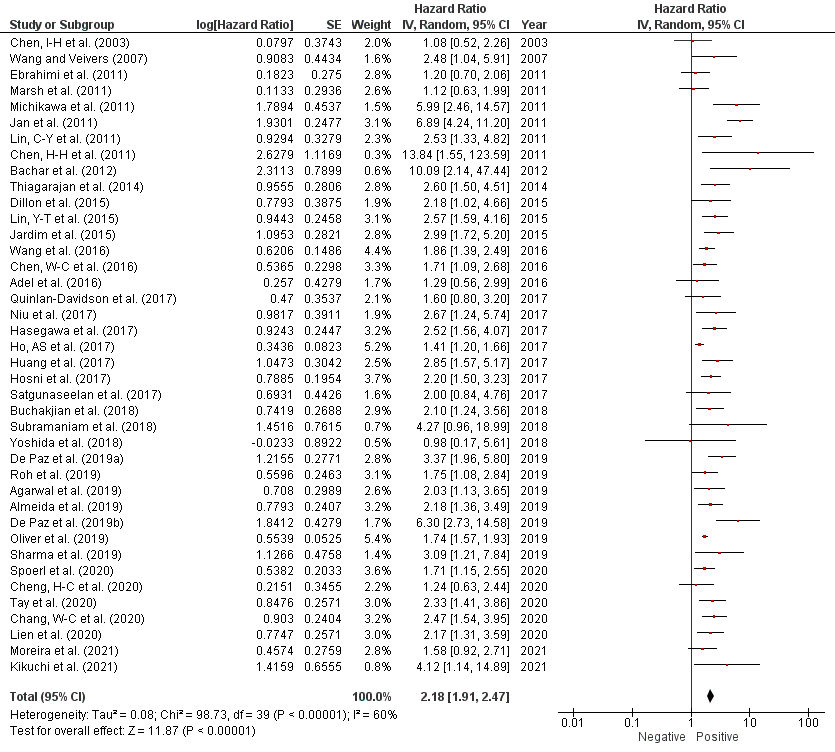


A. Forest plot of hazard ratio for overall survival (OS) comparing patients with ENE (positive) compared with those without ENE (negative).


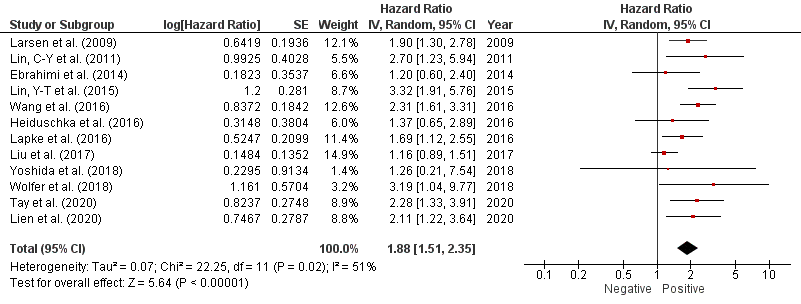


B. Forest plot of hazard ratio for disease-specific survival (DSS) comparing patients with ENE (positive) compared with those without ENE (negative).


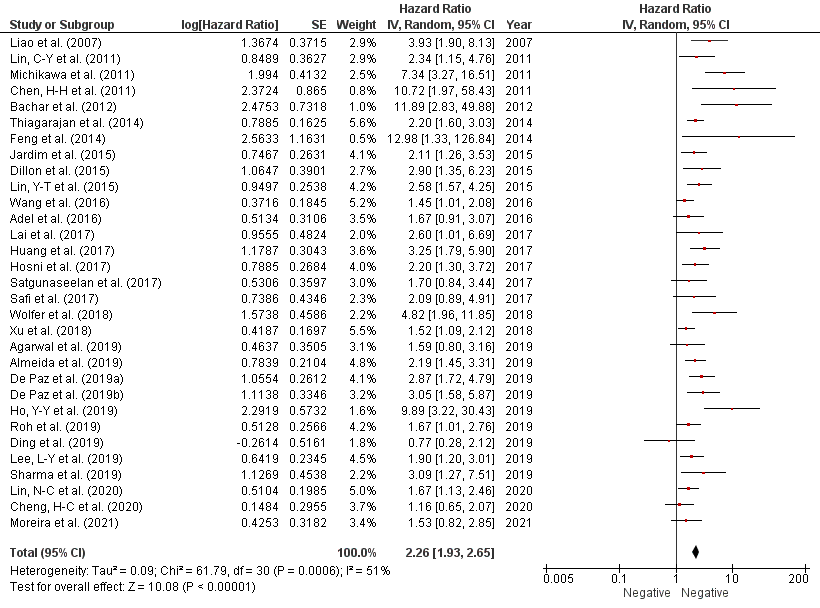


C. Forest plot of hazard ratio for disease-free survival (DFS) comparing patients with ENE (positive) compared with those without ENE (negative).

Supplementary Figure 5. Impact of the perineural invasion (PNI) on survival of patients with oral squamous cell carcinoma (OSCC).


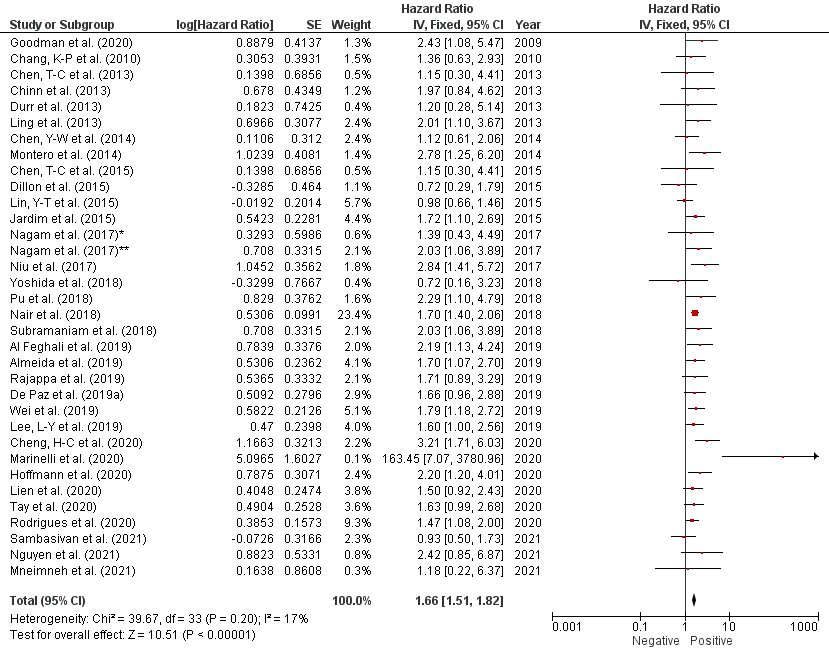


A. Forest plot of hazard ratio for overall survival (OS) comparing patients with PNI (positive) compared with those without PNI (negative).


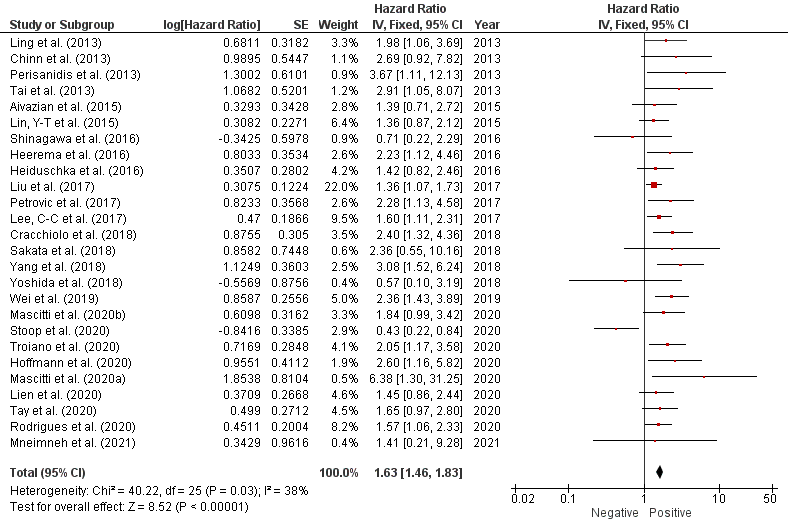


B. Forest plot of hazard ratio for disease-specific survival (DSS) comparing patients with PNI (positive) compared with those without PNI (negative).


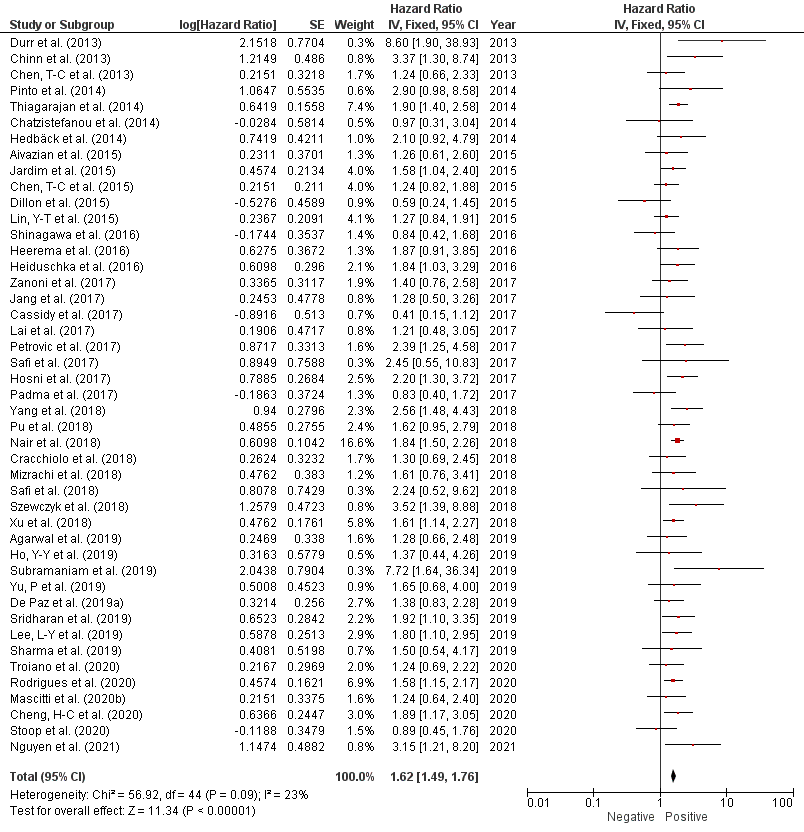


C. Forest plot of hazard ratio for disease-free survival (DFS) comparing patients with PNI (positive) compared with those without PNI (negative).

Supplementary Figure 6. Impact of the lymphovascular invasion (LVI) on survival of patients with oral squamous cell carcinoma (OSCC).


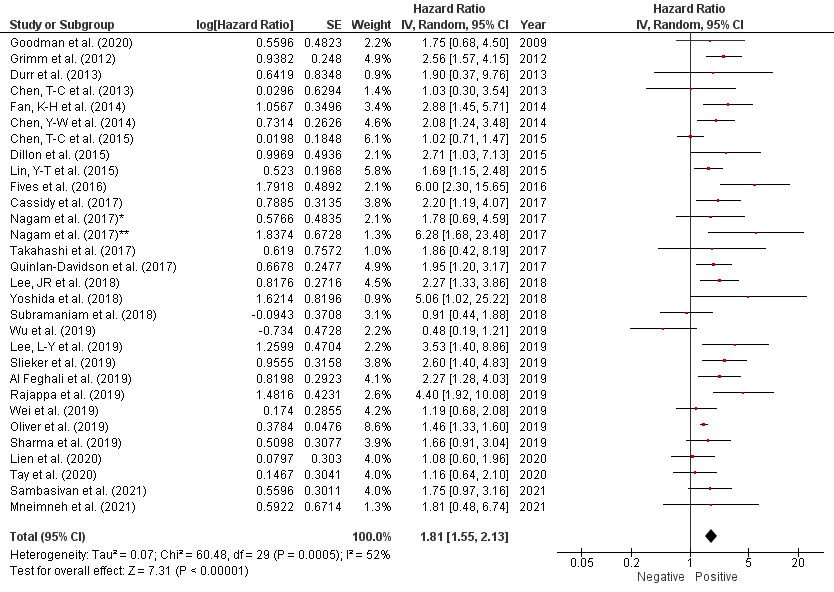


A. Forest plot of hazard ratio for overall survival (OS) comparing patients with LVI (positive) compared with those without LVI (negative).


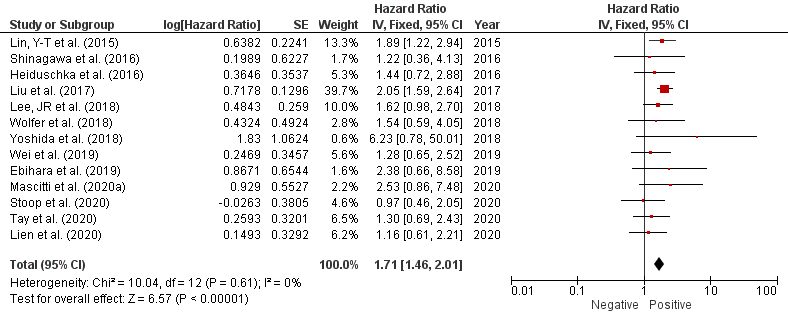


B. Forest plot of hazard ratio for disease-specific survival (DSS) comparing patients with LVI (positive) compared with those without LVI (negative).


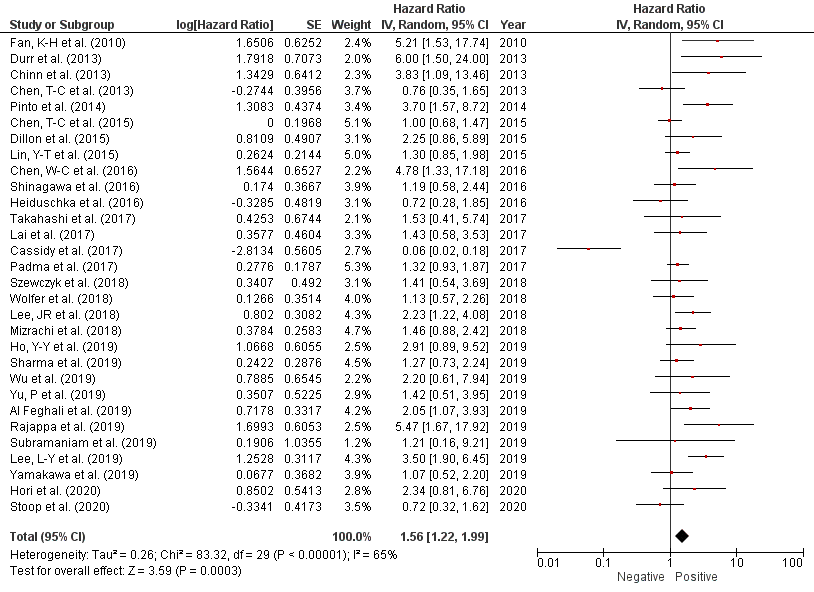


C. Forest plot of hazard ratio for disease-free survival (DFS) comparing patients with LVI (positive) compared with those without LVI (negative).

Supplementary Figure 7. Impact of the surgical margin, regardless of the cut-off value, on survival of patients with oral squamous cell carcinoma (OSCC).


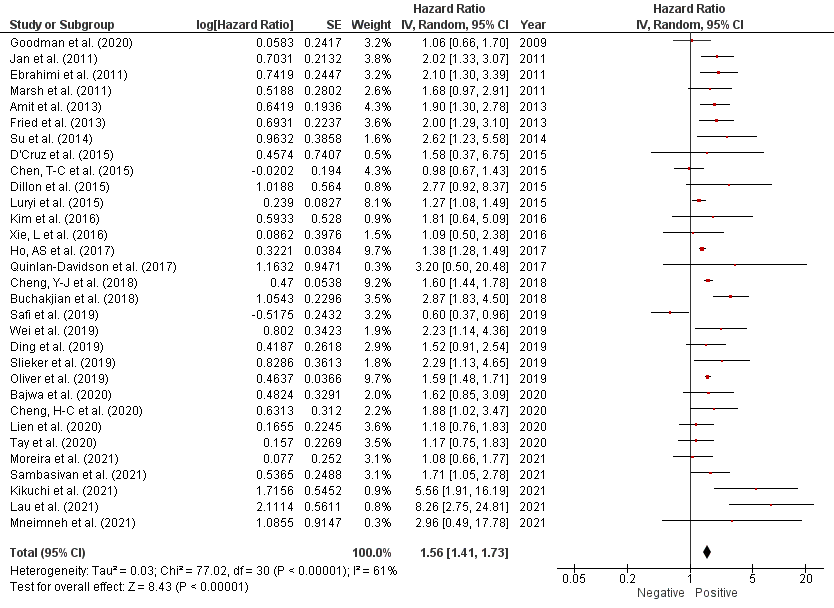


A. Forest plot of hazard ratio for overall survival (OS) comparing patients with a positive margin compared with those with a free (negative) margin.


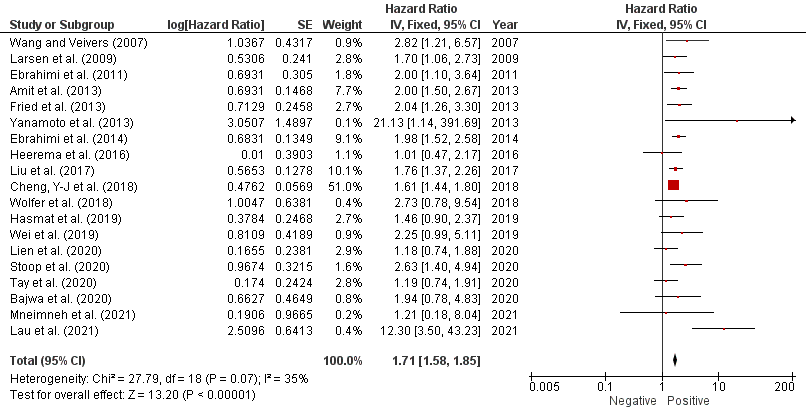


B. Forest plot of hazard ratio for disease-specific survival (DSS) comparing patients with a positive margin compared with those with a free (negative) margin.


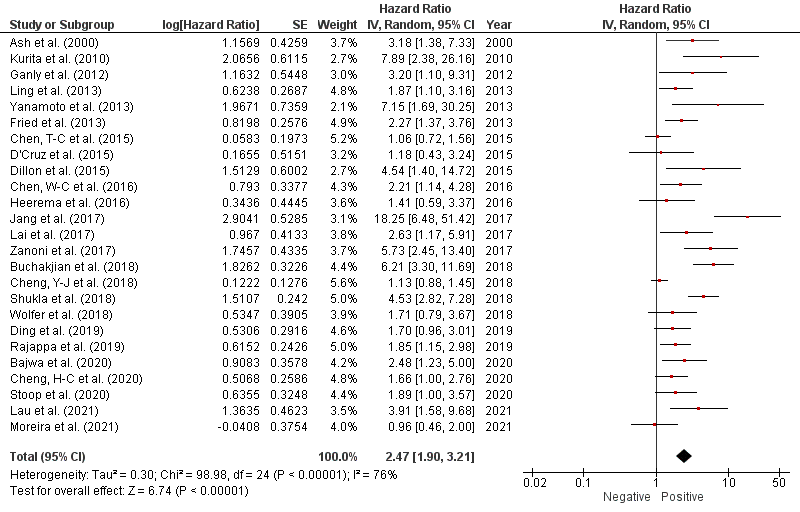


C. Forest plot of hazard ratio for disease-free survival (DFS) comparing patients with a positive margin compared with those with a free (negative) margin.

Supplementary Figure 8. Subgroup analysis of impact of the surgical margin with the cut-off value of 5 mm. Tumors with a surgical margin <5 mm were classified as positive, and tumors with a margin ≥5 mm were designed as negative.


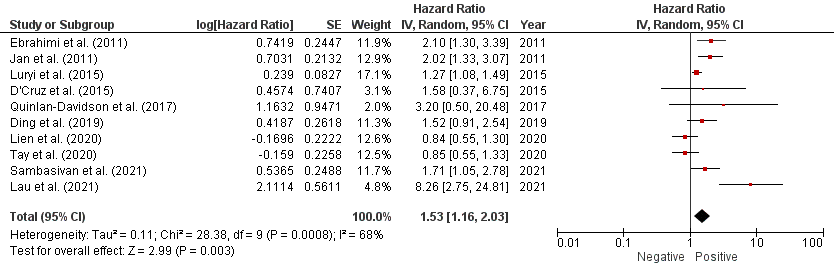


A. Subgroup analysis of impact of the surgical margin set at 5 mm on overall survival (OS).


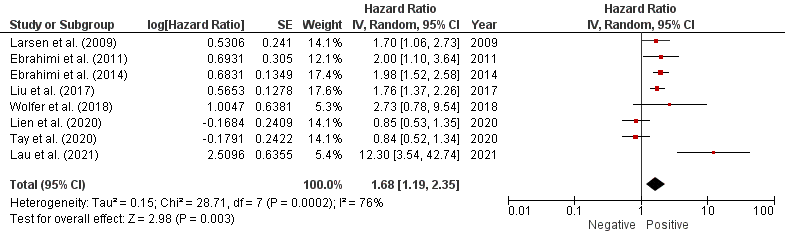


B. Subgroup analysis of impact of the surgical margin set at 5 mm on disease-specific survival (DSS).


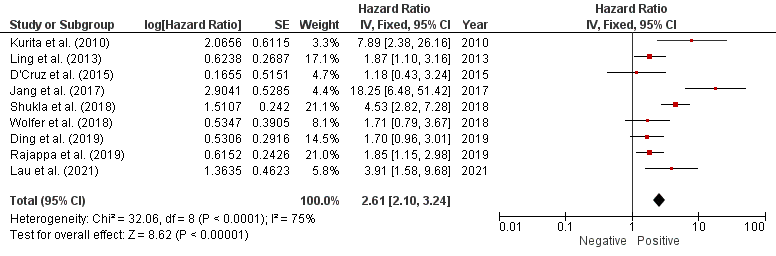


C. Subgroup analysis of impact of the surgical margin set at 5 mm on disease-free survival (DFS).

Supplementary Figure 9. Impact of the tumor thickness on survival of patients with oral squamous cell carcinoma (OSCC).


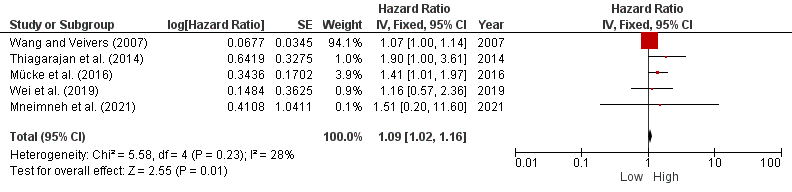


A. Forest plot of hazard ratio for overall survival (OS) comparing patients with high tumor thickness compared with those with low tumor thickness.


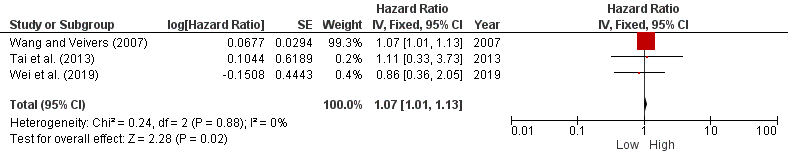


B. Forest plot of hazard ratio for disease-specific survival (DSS) comparing patients with high tumor thickness compared with those with low tumor thickness.


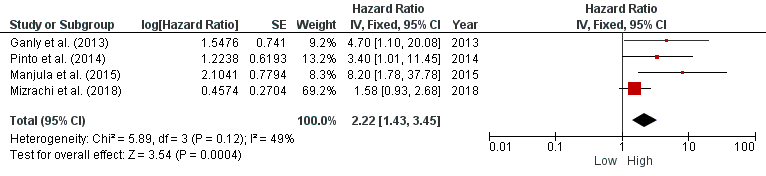


C. Forest plot of hazard ratio for disease-free survival (DFS) comparing patients with high tumor thickness compared with those with low tumor thickness.

Supplementary Figure 10. Impact of the bone invasion on survival of patients with oral squamous cell carcinoma (OSCC).


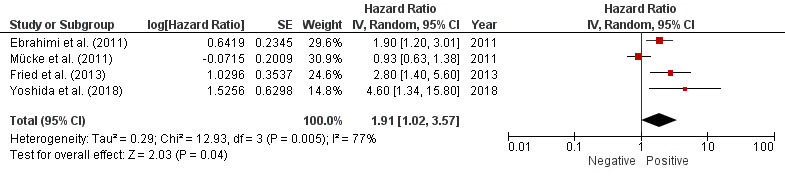


A. Forest plot of hazard ratio for overall survival (OS) comparing patients with bone invasion (positive) compared with those without bone invasion (negative).


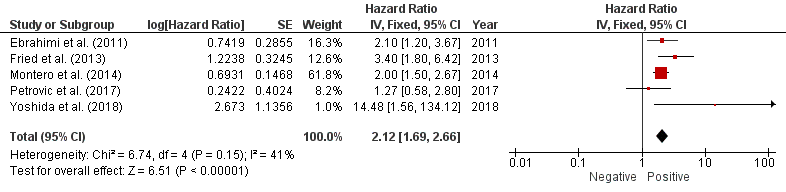


B. Forest plot of hazard ratio for disease-specific survival (DSS) comparing patients with bone invasion (positive) compared with those without bone invasion (negative).


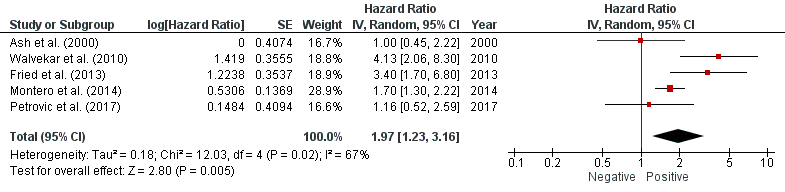


C. Forest plot of hazard ratio for disease-free survival (DFS) comparing patients with bone invasion (positive) compared with those without bone invasion (negative).

Supplementary Figure 11. Effect of cohesive/non-cohesive pattern of invasion on survival of patients with oral squamous cell carcinoma (OSCC).


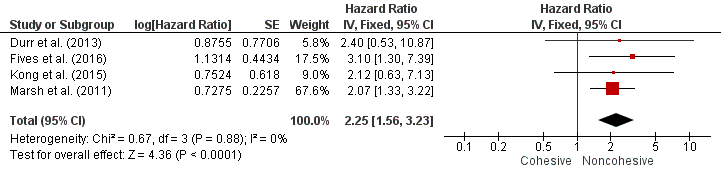


A. Forest plot of hazard ratio for overall survival (OS) comparing tumors with a non-cohesive pattern compared with those with a cohesive pattern.


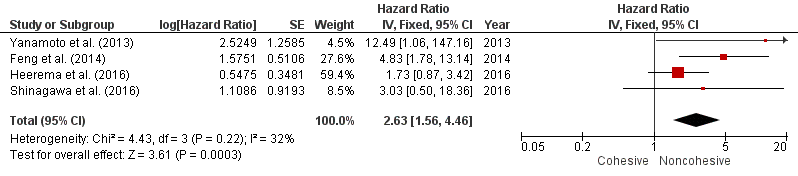


B. Forest plot of hazard ratio for disease-specific survival (DSS) comparing tumors with a non-cohesive pattern compared with those with a cohesive pattern.


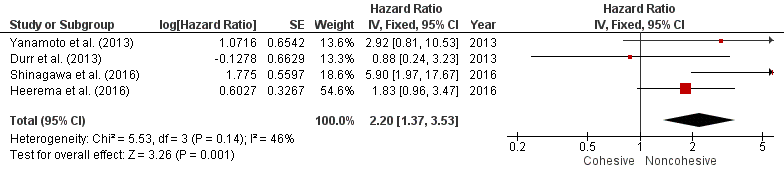


C. Forest plot of hazard ratio for disease-free survival (DFS) comparing tumors with a non-cohesive pattern compared with those with a cohesive pattern.

Supplementary Figure 12. Effect of worst-pattern of invasion (WPOI) on survival of patients with oral squamous cell carcinoma (OSCC).


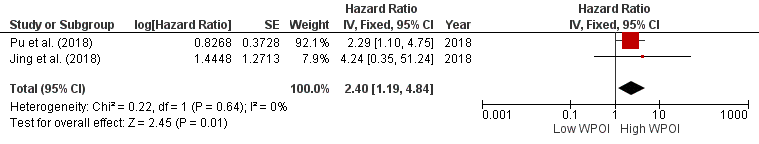


A. Forest plot of hazard ratio for overall survival (OS) comparing tumors with a high WPOI compared with those with a low WPOI.


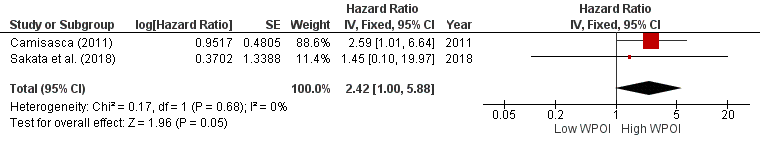


B. Forest plot of hazard ratio for disease-specific survival (DSS) comparing tumors with a high WPOI compared with those with a low WPOI.


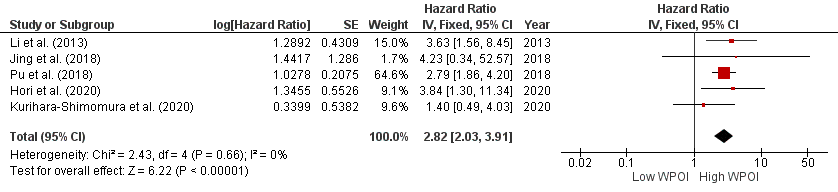


C. Forest plot of hazard ratio for disease-free survival (DFS) comparing tumors with a high WPOI compared with those with a low WPOI.

Supplementary Figure 13. Effect of the tumor budding on survival of patients with oral squamous cell carcinoma (OSCC).


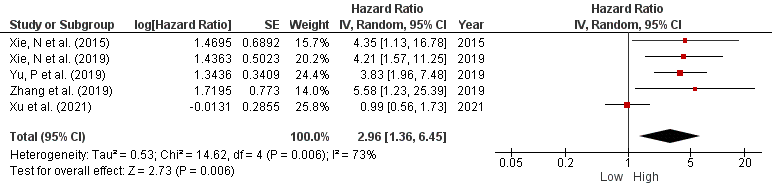


A. Forest plot of hazard ratio for overall survival (OS) comparing tumors with a high activity of tumor budding compared with those with a low activity of tumor budding.


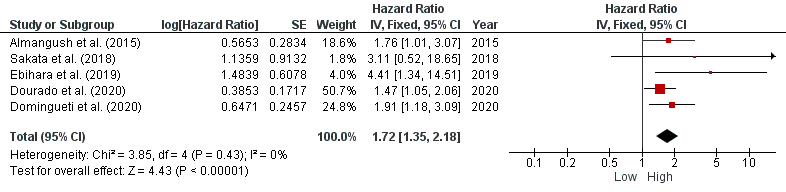


B. Forest plot of hazard ratio for disease-specific survival (DSS) comparing tumors with a high activity of tumor budding compared with those with a low activity of tumor budding.


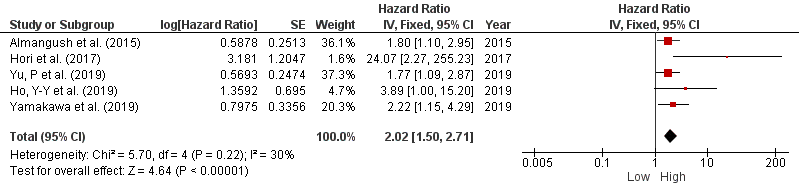


C. Forest plot of hazard ratio for disease-free survival (DFS) comparing tumors with a high activity of tumor budding compared with those with a low activity of tumor budding.

Supplementary Figure 14. Effect of the tumor-stroma ratio on survival of patients with oral squamous cell carcinoma (OSCC).


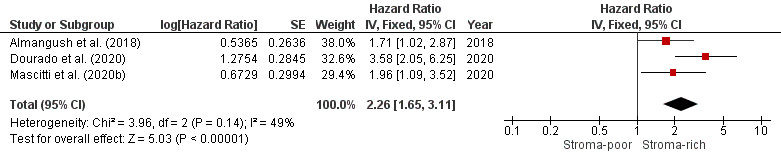


A. Forest plot of hazard ratio for disease-specific survival (DSS) comparing tumors with a stroma-rich compared with those with a stroma-poor.


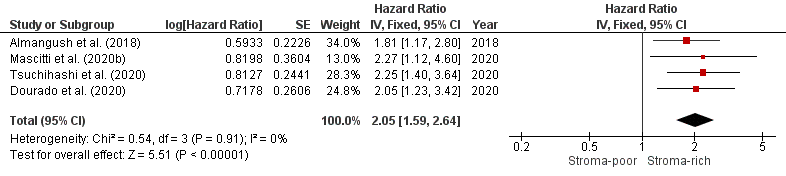


B. Forest plot of hazard ratio for disease-free survival (DFS) comparing tumors with a stroma-rich compared with those with a stroma-poor.
