# Supplementary material for: The Impact of Histopathological Features on the Prognosis of Oral Squamous Cell Carcinoma: A Comprehensive Review and Meta-Analysis
Source: Front Oncol. 2021 Nov 10;11:784924. doi: 10.3389/fonc.2021.784924 (PMC8631280; doi:10.3389/fonc.2021.784924)
Supplement: Supplementary file 1 [file DataSheet_1.zip › Supplementary File 1.DOCX]

Supplementary File 1. Search strategies used on PubMed, Cochrane, Scopus and Web of Science.

**PubMed**

((((("oral squamous cell carcinoma" OR "oral cancer" OR "mouth cancer" OR "tongue cancer" OR "oral cavity cancer" OR "mouth neoplasm" OR tongue OR "oral cavity" OR "tongue neoplasms") AND (prognosis OR prognostic OR prediction OR survival OR outcome OR "disease-free survival" OR "disease-specific survival" OR "overall survival" OR "cancer-specific survival" OR "recurrence-free survival")) AND (marker OR biomarker OR parameter OR characteristic OR feature)) AND (histology OR histological OR histopathology OR histopathological OR morphology OR morphological OR "H&E")) AND ("depth of invasion" OR invasion OR "tumor thickness" OR "tumor length" OR "tumor size" OR "extracapsular spread" OR "extranodal spread" OR extracapsular OR extranodal OR "extranodal extension" OR "extra-nodal extension" OR nodal OR margin OR "surgical margin" OR margins OR "lymphovascular invasion" OR "vascular invasion" OR "vascular infiltration" OR "vascular involvement" OR "lymphatic invasion" OR "bone invasion" OR "bone involvement" OR "bone infiltration" OR perineural OR "perineural invasion" OR "perineural infiltration" OR "neural invasion" OR "neural infiltration" OR "tumor budding" OR budding OR "pattern of invasion" OR "tumor invasion" OR "tumor infiltration" OR "tumor islands" OR keratinized OR keratinizing OR "inflammatory response" OR "immune response" OR "inflammation" OR "inflammatory")) NOT (immunohistochemistry OR immunohistochemical OR IHC)

**Search strategy on Cochrane Library**

*((“oral squamous cell carcinoma” OR “oral cancer” OR “mouth cancer” OR “tongue cancer” OR “oral cavity cancer” OR “mouth neoplasm” OR tongue OR “oral cavity” OR “tongue neoplasms”)) AND ((prognosis OR prognostic OR prediction OR survival OR outcome OR “disease-free survival” OR “disease-specific survival” OR “overall survival” OR “cancer-specific survival” OR “recurrence-free survival”)) AND ((marker OR biomarker OR parameter OR characteristic OR feature )) AND ((histology OR histological OR histopathology OR histopathological OR morphology OR morphological OR “H&E” )) AND ((“depth of invasion” OR invasion OR “tumor thickness” OR “tumor length” OR “tumor size” OR “extracapsular spread” OR “extranodal spread” OR extracapsular OR extranodal OR “extranodal extension” OR “extra-nodal extension” OR nodal OR margin OR “surgical margin” OR margins OR “lymphovascular invasion” OR “vascular invasion” OR “vascular infiltration” OR “vascular involvement” OR “lymphatic invasion” OR “bone invasion” OR “bone involvement” OR “bone infiltration” OR perineural OR “perineural invasion” OR “perineural infiltration” OR “neural invasion” OR “neural infiltration” OR “tumor budding” OR budding OR “pattern of invasion” OR “tumor invasion” OR “tumor infiltration” OR “tumor islands” OR keratinized OR keratinizing OR “inflammatory response” OR “immune response” OR “inflammation” OR “inflammatory”)) AND NOT ((immunohistochemistry OR immunohistochemical OR IHC)) in Title Abstract Keyword

**Scopus**

TITLE-ABS-KEY((("oral squamous cell carcinoma" OR "oral cancer" OR "mouth cancer" OR "tongue cancer" OR "oral cavity cancer" OR "mouth neoplasm" OR tongue OR "oral cavity" OR "tongue neoplasms") AND(prognosis OR prognostic OR prediction OR survival OR outcome OR "disease-free survival" OR "disease-specific survival" OR "overall survival" OR "cancer-specific survival" OR "recurrence-free survival") AND(marker OR biomarker OR parameter OR characteristic OR feature) AND(histology OR histological OR histopathology OR histopathological OR morphology OR morphological OR "H&E" ) AND("depth of invasion" OR invasion OR "tumor thickness" OR "tumor length" OR "tumor size" OR "extracapsular spread" OR "extranodal spread" OR extracapsular OR extranodal OR "extranodal extension" OR "extra-nodal extension" OR nodal OR margin OR "surgical margin" OR margins OR "lymphovascular invasion" OR "vascular invasion" OR "vascular infiltration" OR "vascular involvement" OR "lymphatic invasion" OR "bone invasion" OR "bone involvement" OR "bone infiltration" OR perineural OR "perineural invasion" OR "perineural infiltration" OR "neural invasion" OR "neural infiltration" OR "tumor budding" OR budding OR "pattern of invasion" OR "tumor invasion" OR "tumor infiltration" OR "tumor islands" OR keratinized OR keratinizing OR "inflammatory response" OR "immune response" OR "inflammation" OR "inflammatory")) AND NOT(immunohistochemistry OR immunohistochemical OR IHC))

**Web of Science**

(((((ALL=((“oral squamous cell carcinoma” OR “oral cancer” OR “mouth cancer” OR “tongue cancer” OR “oral cavity cancer” OR “mouth neoplasm” OR tongue OR “oral cavity” OR “tongue neoplasms”))) AND ALL=((prognosis OR prognostic OR prediction OR survival OR outcome OR “disease-free survival” OR “disease-specific survival” OR “overall survival” OR “cancer-specific survival” OR “recurrence-free survival”))) AND ALL=((marker OR biomarker OR parameter OR characteristic OR feature))) AND ALL=((histology OR histological OR histopathology OR histopathological OR morphology OR morphological OR “H&E”))) AND ALL=((“depth of invasion” OR invasion OR “tumor thickness” OR “tumor length” OR “tumor size” OR “extracapsular spread” OR “extranodal spread” OR extracapsular OR extranodal OR “extranodal extension” OR “extra-nodal extension” OR nodal OR margin OR “surgical margin” OR margins OR “lymphovascular invasion” OR “vascular invasion” OR “vascular infiltration” OR “vascular involvement” OR “lymphatic invasion” OR “bone invasion” OR “bone involvement” OR “bone infiltration” OR perineural OR “perineural invasion” OR “perineural infiltration” OR “neural invasion” OR “neural infiltration” OR “tumor budding” OR budding OR “pattern of invasion” OR “tumor invasion” OR “tumor infiltration” OR “tumor islands” OR keratinized OR keratinizing OR “inflammatory response” OR “immune response” OR “inflammation” OR “inflammatory”))) NOT ALL=((immunohistochemistry OR immunohistochemical OR IHC))
