# Supplementary material for: The Impact of Histopathological Features on the Prognosis of Oral Squamous Cell Carcinoma: A Comprehensive Review and Meta-Analysis
Source: Front Oncol. 2021 Nov 10;11:784924. doi: 10.3389/fonc.2021.784924 (PMC8631280; doi:10.3389/fonc.2021.784924)
Supplement: Supplementary file 1 [file DataSheet_1.zip › Supplementary Table 1.DOCX]

Supplementary Table 1. Characteristics of the 273 excluded studies, which had the full-text assessed for eligibility

| Year | Study | Country | Exclusion reason |
| --- | --- | --- | --- |
| 2020 | Carta et al. | Italy | no report of HR and 95% CI |
| 2020 | Rahman et al. | UK | no report of HR and 95% CI |
| 2020 | Bjerkli et al. | Norway | no report of HR and 95% CI |
| 2020 | Hasegawa et al. | Japan | sample and parameter reported in another included study |
| 2020 | Lu et al. | China | parameters not described in the multivariate analysis |
| 2020 | Nur et al. | Ireland | only association analysis |
| 2020 | Almangush et al. | Finland and Brazil | no report of HR and 95% CI |
| 2020 | Mamic et al. | Croatia | parameters not described in the multivariate analysis |
| 2020 | Jani et al. | India | parameters not described in the multivariate analysis |
| 2020 | Shan et al. | China | study design is not appropriate |
| 2020 | Wunschel et al. | Germany | no report of 95% CI |
| 2020 | Zhao et al. | China | parameters not described in the multivariate analysis |
| 2020 | Chuang et al. | Taiwan | only univariate survival analysis |
| 2020 | Kato et al. | Japan | parameters not described in the multivariate analysis |
| 2020 | Larson et al. | USA | no report of 95% CI |
| 2020 | Adeoye et al. | Hong Kong | inclusion of oropharyngeal tumors and only univariate survival analysis |
| 2020 | Tazeen et al. | India | parameters not described in the multivariate analysis |
| 2020 | Singh et al. | India | no report of HR and 95% CI |
| 2020 | Mermod et al. | Switzerland and Australia | study design is not appropriate |
| 2020 | Sowmya et al. | India | only univariate survival analysis |
| 2020 | Salama et al. | USA | parameters not described in the multivariate analysis |
| 2020 | Tervo et al. | Finland | parameters not described in the multivariate analysis |
| 2020 | Alkhadar et al. | UK | only univariate survival analysis |
| 2020 | Matsuzaki et al. | Japan | parameters not described in the multivariate analysis |
| 2020 | Gupta et al. | India | only association analysis |
| 2020 | Sudo et al. | Japan | only association analysis |
| 2020 | Yue et al. | USA | only association analysis |
| 2020 | Siriwardena et al. | Sri Lanka | only association analysis |
| 2020 | Slieker et al. | Netherlands | sample and parameter reported in another included study |
| 2019 | Chatterjee et al. | India | no report of HR and 95% CI |
| 2019 | Morand et al. | Switzerland | only univariate survival analysis |
| 2019 | Cariati et al. | Spain | only univariate survival analysis |
| 2019 | Cariati et al. | Spain | only univariate survival analysis |
| 2019 | Lee et al. | Taiwan | only univariate survival analysis |
| 2019 | Weckx et al. | Germany | inclusion of recurrent cases |
| 2019 | Manjula et al. | India | only association analysis |
| 2019 | Nayanar et al. | India | only association analysis |
| 2019 | Ahmad et al. | USA | inclusion of recurrent cases and second primary tumors |
| 2019 | Subramaniam et al. | India | only univariate survival analysis |
| 2019 | Hasegawa et al. | Japan | only univariate survival analysis |
| 2019 | Nassiri et al. | USA | only univariate survival analysis |
| 2019 | Elseragy et al. | Finland and Brazil | sample and parameter reported in another included study |
| 2019 | Jing et al. | China | sample and parameter reported in another included study |
| 2019 | Książek et al. | Poland | no report of 95% CI |
| 2019 | Shaban et al. | Pakistan | inclusion of oropharyngeal tumors |
| 2019 | Migueláñez-Medrán et al. | Spain | only association analysis |
| 2019 | Miyazawa et al. | Japan | sample based in biopsies |
| 2019 | Okuyama et al. | Japan | only association analysis |
| 2019 | Kim et al. | USA | only association analysis |
| 2019 | Boeve et al. | Netherlands | only univariate survival analysis |
| 2019 | Piazza et al. | Italy | inclusion of oropharyngeal tumors and only univariate survival analysis |
| 2019 | Ghafari et al. | Iran | only association analysis |
| 2019 | Jiang et al. | China | inclusion of oropharyngeal tumors |
| 2018 | Rajappa et al. | India | no report of HR and 95% CI |
| 2018 | Dik et al. | Netherlands | only association analysis |
| 2018 | Subramaniam et al. | India | only univariate survival analysis |
| 2018 | Jardim et al. | Brazil | sample and parameter reported in another included study |
| 2018 | Ahmed et al. | Pakistan | only association analysis |
| 2018 | Siriwardena et al. | Sri Lanka | only association analysis |
| 2018 | Reddy et al. | India | only association analysis |
| 2018 | Shinn et al. | USA | only univariate survival analysis |
| 2018 | Subramaniam et al. | India | only univariate survival analysis |
| 2018 | Abbas et al. | Pakistan | no report of HR and 95% CI |
| 2018 | Weimar et al. | Canada | parameters not described in the multivariate analysis |
| 2018 | De Silva et al. | Sri Lanka | only association analysis |
| 2018 | Khwaja et al. | India | only association analysis |
| 2018 | Cariati et al. | Spain | only univariate survival analysis |
| 2018 | Michikawa et al. | Japan | parameters not described in the multivariate analysis |
| 2018 | Mukoyama et al. | Japan | parameters not described in the multivariate analysis |
| 2018 | Murthy et al. | India | only association analysis |
| 2018 | Shimizu et al. | Japan | study design is not appropriate |
| 2018 | Boxberg et al. | Germany | parameters not described in the multivariate analysis |
| 2018 | Yellapurkar et al. | India | only association analysis |
| 2018 | Troeltzsch et al. | Germany | parameters not described in the multivariate analysis |
| 2018 | Sinha et al. | Canada | only association analysis |
| 2018 | Yamada et al. | Japan | only univariate survival analysis |
| 2018 | Sharma et al. | India | only association analysis |
| 2017 | Lu et al. | USA | parameters not described in the multivariate analysis |
| 2017 | Routray et al. | India | parameters not described in the multivariate analysis |
| 2017 | Peixoto et al. | Brazil | study design is not appropriate |
| 2017 | Jain et al. | India | parameters not described in the multivariate analysis |
| 2017 | Thiagarajan et al. | India | no report of HR and 95% CI |
| 2017 | Boxberg et al. | Germany | no report of HR and 95% CI |
| 2017 | Hosni et al. | Canada | sample and parameter reported in another included study |
| 2017 | Mermod et al. | Switzerland | only association analysis |
| 2017 | Yonezawa et al. | Japan | parameters not described in the multivariate analysis |
| 2017 | Pollom et al. | USA | only association analysis |
| 2017 | Arora et al. | India | only univariate survival analysis |
| 2017 | Kao et al. | Taiwan | parameters not described in the multivariate analysis |
| 2017 | Hakeem et al. | India | only univariate survival analysis |
| 2017 | Sagheb et al. | Germany | only univariate survival analysis |
| 2017 | Zhang et al. | China | parameters not described in the multivariate analysis |
| 2017 | Katz et al. | Israel | only univariate survival analysis |
| 2017 | Huang et al. | China | only univariate survival analysis |
| 2017 | Suton et al. | Croatia | only univariate survival analysis |
| 2017 | Mishra et al. | India | no report of HR and 95% CI |
| 2017 | Dirven et al. | Australia | only univariate survival analysis |
| 2017 | Abdul Wahab et al. | India | only univariate survival analysis |
| 2017 | Imai et al. | Japan | only univariate survival analysis |
| 2017 | Pedersen et al. | Denmark | study design is not appropriate |
| 2017 | Seki et al. | Japan | sample based in biopsies |
| 2017 | Xu et al. | China | inclusion of oropharyngeal tumors |
| 2017 | Spiotto et al. | USA | parameters not described in the multivariate analysis |
| 2017 | Safi et al. | Germany | only univariate survival analysis |
| 2016 | Bittar et al. | Brazil | only association analysis |
| 2016 | Garzino-Demo et al. | Italy | inclusion of oropharyngeal tumors and only univariate survival analysis |
| 2016 | Wu et al. | China | inclusion of oropharyngeal tumors and no report of HR and 95% CI |
| 2016 | Low et al. | Australia | only association analysis |
| 2016 | Nadaf et al. | India | only association analysis |
| 2016 | Xie et al. | China | parameters not described in the multivariate analysis |
| 2016 | Alamgir et al. | Pakistan | no statistical analysis |
| 2016 | Beggan et al. | Ireland | only univariate survival analysis |
| 2016 | Bagordakis et al. | Brazil | parameters not described in the multivariate analysis |
| 2016 | Noble et al. | USA | parameters not described in the multivariate analysis |
| 2016 | Nair et al. | India | no report of HR and 95% CI |
| 2016 | Nakashima et al. | Japan | parameters not described in the multivariate analysis |
| 2016 | Nandita et al. | India | only association analysis |
| 2016 | Seki et al. | Japan | sample based in biopsies |
| 2016 | Alaeddini and Etemad-Moghadam | Iran | only association analysis |
| 2016 | Majumdar et al. | India | sample based in biopsies and only univariate survival analysis |
| 2016 | Laske et al. | Switzerland | only univariate survival analysis |
| 2015 | Sawazaki-Calone et al. | Brazil | parameters not described in the multivariate analysis |
| 2015 | Lopez-Cedrún and de Llano | Spain | inclusion of oropharyngeal tumors |
| 2015 | Osaka et al. | Japan | parameters not described in the multivariate analysis |
| 2015 | Mani et al. | India | only univariate survival analysis |
| 2015 | Kolokythas et al. | USA | only univariate survival analysis |
| 2015 | Angadi et al. | India | only association analysis |
| 2015 | Liao et al. | China | parameters not described in the multivariate analysis |
| 2015 | Liu et al. | China | inclusion of salivary gland tumors |
| 2015 | Wang et al. | China | parameters not described in the multivariate analysis |
| 2015 | Chen et al. | Taiwan | only univariate survival analysis |
| 2015 | Petera et al. | Czech Republic | no report of HR and 95% CI |
| 2015 | Li et al. | China | study design is not appropriate |
| 2015 | Xie et al. | China | sample and parameter reported in another included study |
| 2015 | Attramadal. et al. | Norway | no report of HR and 95% CI |
| 2015 | Jensen et al. | Denmark | study design is not appropriate |
| 2015 | Varsha et al. | India | only association analysis |
| 2015 | Tarsitano et al. | Italy | only association analysis |
| 2015 | Affonso et al. | Brazil | no report of HR and 95% CI |
| 2015 | Tarsitano et al. | Italy | only univariate survival analysis |
| 2015 | Matsui et al. | Japan | only univariate survival analysis |
| 2015 | Imayama et al. | Japan | no report of HR and 95% CI |
| 2015 | Matsushita et al. | Japan | only univariate survival analysis |
| 2014 | Sandu et al. | Switzerland | only univariate survival analysis |
| 2014 | Song et al. | China | parameters not described in the multivariate analysis |
| 2014 | Patel et al. | USA | inclusion of oropharyngeal tumors and only univariate survival analysis |
| 2014 | Yeh et al. | Taiwan | only association analysis |
| 2014 | Tinhofer et al. | Germany | inclusion of head and neck tumors |
| 2014 | Dik et al. | Netherlands | only univariate survival analysis |
| 2014 | Siow et al. | Malaya | parameters not described in the multivariate analysis |
| 2014 | Almangush et al. | Finland | sample and parameter reported in another included study |
| 2014 | Balasubramanian et al. | Australia | only univariate survival analysis |
| 2014 | Yu, S-Y et al. | Taiwan | parameters not described in the multivariate analysis |
| 2014 | Yamamoto et al. | Japan | only univariate survival analysis |
| 2014 | Yu et al. | Taiwan | sample and parameter reported in another included study |
| 2014 | Rodrigues et al. | Brazil | parameters not described in the multivariate analysis |
| 2013 | Yang et al. | Taiwan | only univariate survival analysis |
| 2013 | Hilly et al. | Israel | only univariate survival analysis |
| 2013 | Yuasa-Nakagawa et al. | Japan | only univariate survival analysis |
| 2013 | Libório et al. | Brazil | only association analysis |
| 2013 | Monevska et al. | Macedonia | only univariate survival analysis |
| 2013 | Chinn et al. | USA | parameters not described in the multivariate analysis |
| 2013 | Sittitrai et al. | India | only univariate survival analysis |
| 2013 | Nikitakis et al. | USA | only association analysis |
| 2013 | Sopka et al. | USA | parameters not described in the multivariate analysis |
| 2013 | Goldstein et al. | Canada | parameters not described in the multivariate analysis |
| 2013 | Moura et al. | Portugal | parameters not described in the multivariate analysis |
| 2013 | Chen et al. | Taiwan | sample and parameter reported in another included study |
| 2013 | Nagata et al. | Japan | sample and parameter reported in another included study |
| 2012 | Huang et al. | Taiwan | sample and parameter reported in another included study |
| 2012 | Dissanayaka et al. | Sri Lanka | no report of HR and 95% CI |
| 2012 | Chaturvedi et al. | India | no report of HR and 95% CI |
| 2012 | Tai et al. | Taiwan | sample and parameter reported in another included study |
| 2012 | Melchers et al. | Netherlands | only univariate survival analysis |
| 2012 | Yanamoto et al. | Japan | only univariate survival analysis |
| 2012 | Matos et al. | Brazil | only association analysis |
| 2011 | Ch’ng et al. | Australia | only univariate survival analysis |
| 2011 | Bachar et al. | Canada and Israel | parameters not described in the multivariate analysis |
| 2011 | Yang et al. | Taiwan | only association analysis |
| 2011 | Tsang et al. | Hong Kong | no report of HR and 95% CI |
| 2011 | Lin et al. | Taiwan | parameters not described in the multivariate analysis |
| 2011 | Wang et al. | China | sample and parameter reported in another included study |
| 2011 | Myers et al. | USA | only univariate survival analysis |
| 2011 | Yoshizawa et al. | Japan | parameters not described in the multivariate analysis |
| 2011 | Lundqvist et al. | Sweden | only association analysis |
| 2011 | Theocharis et al. | Greece | inconsistence between parameter and its definition |
| 2010 | Chatzistamou et al. | France | only association analysis |
| 2010 | Chang et al. | Taiwan | no report of HR and 95% CI |
| 2010 | Vered et al. | Israel | parameters not described in the multivariate analysis |
| 2010 | Soudry et al. | Israel | parameters not described in the multivariate analysis |
| 2009 | Muñoz-Guerra et al. | Spain | parameters not described in the multivariate analysis |
| 2009 | Estilo et al. | USA | no report of HR and 95% CI |
| 2009 | Tadbir et al. | Iran | only association analysis |
| 2009 | González-García et al. | Spain | only univariate survival analysis |
| 2009 | Roh et al. | Republic of Korea | parameters not described in the multivariate analysis |
| 2009 | Shaw et al. | UK | only univariate survival analysis |
| 2009 | Bier-Laning et al. | USA | only univariate survival analysis |
| 2009 | Camisasca et al. | Brazil | sample and parameter reported in another included study |
| 2009 | Jung et al. | South Korea | only univariate survival analysis |
| 2008 | Chen et al. | Taiwan | only association analysis |
| 2008 | Arduino et al. | Italy | parameters not described in the multivariate analysis |
| 2008 | Siriwardena et al. | Sri Lanka | only association analysis |
| 2008 | González-García et al. | Spain | only univariate survival analysis |
| 2008 | Vashist et al. | Germany | parameters not described in the multivariate analysis |
| 2008 | Patel et al. | Australia | parameters not described in the multivariate analysis |
| 2008 | An et al. | South Korea | no report of HR and 95% CI |
| 2008 | Søland et al. | Norway | parameters not described in the multivariate analysis |
| 2008 | Yang et al. | Taiwan | parameters not described in the multivariate analysis |
| 2007 | Keski-Säntti et al. | Finland | only univariate survival analysis |
| 2006 | Kane et al. | India | only association analysis |
| 2006 | Garzino-Demo et al. | Italy | only univariate survival analysis |
| 2006 | Kurokawa et al. | Japan | no report of 95% CI |
| 2006 | Clark et al. | Canada | parameters not described in the multivariate analysis |
| 2005 | Brandwein-Gensler et al. | USA | only association analysis in the cohort with tongue tumors |
| 2005 | Sasaki et al. | UK | only association analysis |
| 2004 | Wenzel et al. | Germany | inclusion of oropharyngeal tumors |
| 2004 | Kurokawa et al. | Japan | only univariate survival analysis |
| 2004 | Kurita et al. | Japan | no report of HR and 95% CI |
| 2004 | Shaw et al. | England | no report of HR and 95% CI |
| 2004 | Lim et al. | South Korea | only univariate survival analysis |
| 2004 | Sparano et al. | USA | study design is not appropriate |
| 2003 | O-charoenrat et al. | UK | no report of 95% CI |
| 2003 | Sutton et al. | UK | no report of 95% CI |
| 2003 | Sawair et al. | North Ireland | no report of 95% CI |
| 2003 | Woolgar et al. | England | no report of HR and 95% CI |
| 2003 | Sessions et al. | USA | inclusion of oropharyngeal tumors |
| 2003 | Okada et al. | Japan | Study design is not appropriate |
| 2003 | O'Brien et al. | Australia | inclusion of oropharyngeal tumors |
| 2003 | O'Brien et al. | Australia | only univariate survival analysis |
| 2003 | Sheahan et al. | Ireland | only univariate survival analysis |
| 2002 | Bundgaard et al. | Denmark | only univariate survival analysis |
| 2002 | Gonzalez-Moles et al. | Spain | no report of HR and 95% CI |
| 2002 | Yuen et al. | China | only association analysis |
| 2002 | Kurokawa et al. | Japan | no report of 95% CI |
| 2000 | Klotch et al. | USA | only association analysis |
| 2000 | Al-Rajhi et al. | Saudi Arabi | no report of HR and 95% CI |
| 2000 | Kowalski et al. | Brazil | no report of HR and 95% CI |
| 2000 | Yuen et al. | China | no report of HR and 95% CI |
| 2000 | Ma'aita et al. | Jordan | parameters not described in the multivariate analysis |
| 1999 | Nakayama et al. | Japan | parameters not described in the multivariate analysis |
| 1999 | Chen et al. | Taiwan | parameters not described in the multivariate analysis |
| 1999 | Altemani et al. | Brazil | only univariate survival analysis |
| 1999 | Woolgar et al. | UK | only univariate survival analysis |
| 1999 | Woolgar | England | only association analysis |
| 1999 | Howaldt et al. | Germany | study design is not appropriate |
| 1999 | Ünal. et al. | Turkey | no report of HR and 95% CI |
| 1999 | Spiro et al. | USA | only univariate survival analysis |
| 1998 | Asakage et al. | Japan | no report of HR and 95% CI |
| 1998 | Rubio Bueno et al. | Spain | inclusion of oropharyngeal tumors and no report of HR and 95% CI |
| 1998 | Hoșal. et al. | Turkey | no report of HR and 95% CI |
| 1998 | Kurokawa et al. | Japan | no report of 95% CI |
| 1998 | Matsuura et al. | Japan | only univariate survival analysis |
| 1997 | Fukano et al. | Japan | only univariate survival analysis |
| 1997 | Parsons et al. | USA | no report of HR and 95% CI |
| 1996 | Bundgaard et al. | Denmark | sample based in biopsies |
| 1996 | Teixeira et al. | Brazil | no report of HR and 95% CI |
| 1995 | Woolgar et al. | UK | inclusion of oropharyngeal tumors and only univariate survival analysis |
| 1995 | Guiney | Australia | no access to the full article |
| 1995 | Martínez-Gimeno et al. | Spain | only association analysis |
| 1995 | Woolgar and Scott | England | only association analysis |
| 1994 | Odell et al. | England | only univariate survival analysis |
| 1994 | Morton et al. | New Zealand | only association analysis |
| 1994 | Williams et al. | USA | parameters not described in the multivariate analysis |
| 1994 | Sarioğlu et al. | Turkey | only association analysis |
| 1992 | Gomez et al. | USA | no access to the full article |
| 1991 | Brennan et al. | USA | only univariate survival analysis |
| 1990 | Maddox and Urist | England | no access to the full article |
| 1989 | Brown et al. | USA | no report of HR and 95% CI |
| 1989 | Nathanson et al. | Sweden | only univariate survival analysis |
| 1987 | Urist et al. | USA | no report of HR and 95% CI |
| 1986 | Spiro et al. | USA | only univariate survival analysis |
| 1981 | Rollo et al. | USA | only univariate survival analysis |
| 1976 | Shah et al. | USA | only association analysis |

Abbas SA, Saeed J, Tariq MU, Baksh AR, Hashmi S. Clinicopathological prognostic factors of oral squamous cell carcinoma: An experience of a tertiary care hospital. J Pak Med Assoc. 2018 Jul;68(7):1115-1119. PMID: 30317316.

Abdul Wahab PU, Madhulaxmi M, Senthilnathan P, Muthusekhar MR, Pradeep D, Abhinav RP. Comparisons of clinicopathologic characteristics among early and late stage of oral squamous cell carcinoma. J. Pharm. Sci & Res. 2017; 9(11):2147-2150.

Adeoye J, Thomson P, Choi SW. Prognostic significance of multi-positive invasive histopathology in oral cancer. J Oral Pathol Med. 2020 Nov;49(10):1004-1010. doi: 10.1111/jop.13086. Epub 2020 Aug 20. PMID: 32740985.

Affonso VR, Montoro JR, Freitas LC, Saggioro FP, Souza Ld, Mamede RC. Peritumoral infiltrate in the prognosis of epidermoid carcinoma of the oral cavity. Braz J Otorhinolaryngol. 2015 Jul-Aug;81(4):416-21. doi: 10.1016/j.bjorl.2014.09.010. Epub 2015 Jun 10. PMID: 26141206.

Ahmad JG, Namin AW, Jorgensen JB, Zitsch RP 3rd, Layfield LJ. Mandibular Invasion by Oral Squamous Cell Carcinoma: Clinicopathologic Features of 74 Cases. Otolaryngol Head Neck Surg. 2019 Jun;160(6):1034-1041. doi: 10.1177/0194599818821859. Epub 2019 Jan 1. PMID: 30598057.

Ahmed SQ, Junaid M, Awan S, Kazi M, Khan HU, Halim S. Frequency of Cervical Nodal Metastasis in Early-Stage Squamous Cell Carcinoma of the Tongue. Int Arch Otorhinolaryngol. 2018 Apr;22(2):136-140. doi: 10.1055/s-0037-1603626. Epub 2017 Jun 6. PMID: 29619101; PMCID: PMC5882373.

Al-Rajhi N, Khafaga Y, El-Husseiny J, Saleem M, Mourad W, Al-Otieschan A, Al-Amro A. Early stage carcinoma of oral tongue: prognostic factors for local control and survival. Oral Oncol. 2000 Nov;36(6):508-14. doi: 10.1016/s1368-8375(00)00042-7. PMID: 11036243.

Alaeddini M, Etemad-Moghadam S. Correlation between invasion mode and the histologic risk assessment model in oral squamous cell carcinoma. Oral Maxillofac Surg. 2016 Dec;20(4):353-358. doi: 10.1007/s10006-016-0572-3. Epub 2016 Aug 9. PMID: 27502395.

Alamgir MM, Jamal Q, Mirza T. Conventional clinical and prognostic variables in 150 oral squamous cell carcinoma cases from the indigenous population of Karachi. Pak J Med Sci. 2016 May-Jun;32(3):672-6. doi: 10.12669/pjms.323.9905. PMID: 27375712; PMCID: PMC4928421.

Alkhadar H, Macluskey M, White S, Ellis I. Perineural invasion in oral squamous cell carcinoma: Incidence, prognostic impact and molecular insight. J Oral Pathol Med. 2020 Nov;49(10):994-1003. doi: 10.1111/jop.13069. Epub 2020 Jun 25. PMID: 32533593.

Almangush A, Bello IO, Keski-Säntti H, Mäkinen LK, Kauppila JH, Pukkila M, Hagström J, Laranne J, Tommola S, Nieminen O, Soini Y, Kosma VM, Koivunen P, Grénman R, Leivo I, Salo T. Depth of invasion, tumor budding, and worst pattern of invasion: prognostic indicators in early-stage oral tongue cancer. Head Neck. 2014 Jun;36(6):811-8. doi: 10.1002/hed.23380. Epub 2013 Sep 2. PMID: 23696499; PMCID: PMC4229066.

Almangush A, Mäkitie AA, Hagström J, Haglund C, Kowalski LP, Nieminen P, Coletta RD, Salo T, Leivo I. Cell-in-cell phenomenon associates with aggressive characteristics and cancer-related mortality in early oral tongue cancer. BMC Cancer. 2020 Sep 3;20(1):843. doi: 10.1186/s12885-020-07342-x. PMID: 32883229; PMCID: PMC7469910.

Altemani AM, Guimarães P, Metze K, Queiroz LS. Quantitative analysis of modes of invasion and lymph node metastases in oral squamous cell carcinoma. Neoplasma. 1999;46(5):323-8. PMID: 10665851.

An SY, Jung EJ, Lee M, Kwon TK, Sung MW, Jeon YK, Kim KH. Factors related to regional recurrence in early stage squamous cell carcinoma of the oral tongue. Clin Exp Otorhinolaryngol. 2008 Sep;1(3):166-70. doi: 10.3342/ceo.2008.1.3.166. Epub 2008 Sep 30. PMID: 19434251; PMCID: PMC2671749.

Angadi PV, Patil PV, Hallikeri K, Mallapur MD, Hallikerimath S, Kale AD. Tumor budding is an independent prognostic factor for prediction of lymph node metastasis in oral squamous cell carcinoma. Int J Surg Pathol. 2015 Apr;23(2):102-10. doi: 10.1177/1066896914565022. Epub 2015 Jan 5. PMID: 25559273.

Arduino PG, Carrozzo M, Chiecchio A, Broccoletti R, Tirone F, Borra E, Bertolusso G, Gandolfo S. Clinical and histopathologic independent prognostic factors in oral squamous cell carcinoma: a retrospective study of 334 cases. J Oral Maxillofac Surg. 2008 Aug;66(8):1570-9. doi: 10.1016/j.joms.2007.12.024. PMID: 18634942.

Arora A, Husain N, Bansal A, Neyaz A, Jaiswal R, Jain K, Chaturvedi A, Anand N, Malhotra K, Shukla S. Development of a New Outcome Prediction Model in Early-stage Squamous Cell Carcinoma of the Oral Cavity Based on Histopathologic Parameters With Multivariate Analysis: The Aditi-Nuzhat Lymph-node Prediction Score (ANLPS) System. Am J Surg Pathol. 2017 Jul;41(7):950-960. doi: 10.1097/PAS.0000000000000843. PMID: 28346327.

Asakage T, Yokose T, Mukai K, Tsugane S, Tsubono Y, Asai M, Ebihara S. Tumor thickness predicts cervical metastasis in patients with stage I/II carcinoma of the tongue. Cancer. 1998 Apr 15;82(8):1443-8. doi: 10.1002/(sici)1097-0142(19980415)82:8<1443::aid-cncr2>3.0.co;2-a. PMID: 9554518.

Attramadal CG, Kumar S, Boysen ME, Dhakal HP, Nesland JM, Bryne M. Tumor Budding, EMT and Cancer Stem Cells in T1-2/N0 Oral Squamous Cell Carcinomas. Anticancer Res. 2015 Nov;35(11):6111-20. PMID: 26504037.

Bachar G, Hod R, Goldstein DP, Irish JC, Gullane PJ, Brown D, Gilbert RW, Hadar T, Feinmesser R, Shpitzer T. Outcome of oral tongue squamous cell carcinoma in patients with and without known risk factors. Oral Oncol. 2011 Jan;47(1):45-50. doi: 10.1016/j.oraloncology.2010.11.003. Epub 2010 Dec 16. PMID: 21167767.

Bagordakis E, Sawazaki-Calone I, Macedo CC, Carnielli CM, de Oliveira CE, Rodrigues PC, Rangel AL, Dos Santos JN, Risteli J, Graner E, Salo T, Paes Leme AF, Coletta RD. Secretome profiling of oral squamous cell carcinoma-associated fibroblasts reveals organization and disassembly of extracellular matrix and collagen metabolic process signatures. Tumour Biol. 2016 Jul;37(7):9045-57. doi: 10.1007/s13277-015-4629-y. Epub 2016 Jan 13. PMID: 26762409.

Balasubramanian D, Ebrahimi A, Gupta R, Gao K, Elliott M, Palme CE, Clark JR. Tumour thickness as a predictor of nodal metastases in oral cancer: comparison between tongue and floor of mouth subsites. Oral Oncol. 2014 Dec;50(12):1165-8. doi: 10.1016/j.oraloncology.2014.09.012. Epub 2014 Oct 11. PMID: 25307875.

Beggan C, Fives C, O'Leary G, Sheahan P, Heffron CC, Feeley L. Pattern of invasion and lymphovascular invasion in squamous cell carcinoma of the floor of the mouth: an interobserver variability study. Histopathology. 2016 Dec;69(6):914-920. doi: 10.1111/his.13014. Epub 2016 Aug 30. PMID: 27271979.

Bier-Laning CM, Durazo-Arvizu R, Muzaffar K, Petruzzelli GJ. Primary tumor thickness as a risk factor for contralateral cervical metastases in T1/T2 oral tongue squamous cell carcinoma. Laryngoscope. 2009 May;119(5):883-8. doi: 10.1002/lary.20141. PMID: 19180635.

Bittar RF, Ferraro HP, Ribas MH, Lehn CN. Predictive factors of occult neck metastasis in patients with oral squamous cell carcinoma. Braz J Otorhinolaryngol. 2016 Sep-Oct;82(5):543-7. doi: 10.1016/j.bjorl.2015.09.005. Epub 2015 Dec 17. PMID: 26749457.

Bjerkli IH, Hadler-Olsen E, Nginamau ES, Laurvik H, Søland TM, Costea DE, Uhlin-Hansen L, Steigen SE. A combined histo-score based on tumor differentiation and lymphocytic infiltrate is a robust prognostic marker for mobile tongue cancer. Virchows Arch. 2020 Dec;477(6):865-872. doi: 10.1007/s00428-020-02875-9. Epub 2020 Jun 30. PMID: 32607687; PMCID: PMC7683438.

Boeve K, Melchers LJ, Schuuring E, Roodenburg JL, Halmos GB, van Dijk BA, van der Vegt B, Witjes MJ. Addition of tumour infiltration depth and extranodal extension improves the prognostic value of the pathological TNM classification for early-stage oral squamous cell carcinoma. Histopathology. 2019 Sep;75(3):329-337. doi: 10.1111/his.13886. Epub 2019 Jul 29. PMID: 31021008; PMCID: PMC6851684.

Boxberg M, Götz C, Haidari S, Dorfner C, Jesinghaus M, Drecoll E, Boskov M, Wolff KD, Weichert W, Haller B, Kolk A. Immunohistochemical expression of CD44 in oral squamous cell carcinoma in relation to histomorphological parameters and clinicopathological factors. Histopathology. 2018 Oct;73(4):559-572. doi: 10.1111/his.13496. Epub 2018 Jul 23. PMID: 29468726.

Boxberg M, Jesinghaus M, Dorfner C, Mogler C, Drecoll E, Warth A, Steiger K, Bollwein C, Meyer P, Wolff KD, Kolk A, Weichert W. Tumour budding activity and cell nest size determine patient outcome in oral squamous cell carcinoma: proposal for an adjusted grading system. Histopathology. 2017 Jun;70(7):1125-1137. doi: 10.1111/his.13173. Epub 2017 Mar 28. PMID: 28122134.

Brandwein-Gensler M, Teixeira MS, Lewis CM, Lee B, Rolnitzky L, Hille JJ, Genden E, Urken ML, Wang BY. Oral squamous cell carcinoma: histologic risk assessment, but not margin status, is strongly predictive of local disease-free and overall survival. Am J Surg Pathol. 2005 Feb;29(2):167-78. doi: 10.1097/01.pas.0000149687.90710.21. PMID: 15644773.

Brennan CT, Sessions DG, Spitznagel EL Jr, Harvey JE. Surgical pathology of cancer of the oral cavity and oropharynx. Laryngoscope. 1991 Nov;101(11):1175-97. doi: 10.1288/00005537-199111000-00006. PMID: 1943419.

Brown B, Barnes L, Mazariegos J, Taylor F, Johnson J, Wagner RL. Prognostic factors in mobile tongue and floor of mouth carcinoma. Cancer. 1989 Sep 15;64(6):1195-202. doi: 10.1002/1097-0142(19890915)64:6<1195::aid-cncr2820640606>3.0.co;2-7. PMID: 2766218.

Bundgaard T, Bentzen SM, Wildt J, Sørensen FB, Søgaard H, Nielsen JE. Histopathologic, stereologic, epidemiologic, and clinical parameters in the prognostic evaluation of squamous cell carcinoma of the oral cavity. Head Neck. 1996 Mar-Apr;18(2):142-52. doi: 10.1002/(SICI)1097-0347(199603/04)18:2<142::AID-HED6>3.0.CO;2-1. PMID: 8647680.

Bundgaard T, Rossen K, Henriksen SD, Charabi S, Søgaard H, Grau C. Histopathologic parameters in the evaluation of T1 squamous cell carcinomas of the oral cavity. Head Neck. 2002 Jul;24(7):656-60. doi: 10.1002/hed.10120. PMID: 12112539.

Camisasca DR, Honorato J, Bernardo V, da Silva LE, da Fonseca EC, de Faria PA, Dias FL, Lourenço Sde Q. Expression of Bcl-2 family proteins and associated clinicopathologic factors predict survival outcome in patients with oral squamous cell carcinoma. Oral Oncol. 2009 Mar;45(3):225-33. doi: 10.1016/j.oraloncology.2008.05.021. Epub 2008 Aug 19. PMID: 18715811.

Cariati P, Cabello Serrano A, Fernandez Solis J, Martinez Lara I. Distribution of cervical metastasis in tongue cancer: Are occult metastases predictable? A retrospective study of 117 oral tongue carcinomas. J Craniomaxillofac Surg. 2018 Jan;46(1):155-161. doi: 10.1016/j.jcms.2017.10.009. Epub 2017 Oct 13. PMID: 29174473.

Cariati P, Cabello Serrano A, Roman Ramos M, Sanchez Lopez D, Fernandez Solis J, Martinez Lara I. Behavior of squamous cell carcinoma of the floor of the mouth. Is supraomohyoid neck dissection sufficiently safe to manage clinically N0 patients? Acta Otorrinolaringol Esp (Engl Ed). 2019 Mar-Apr;70(2):68-73. English, Spanish. doi: 10.1016/j.otorri.2018.02.007. PMID: 29759299.

Cariati P, Marin Fernandez AB, Hernandez Vila C, Fernandez Solis J, Garcia Medina B, Martinez Lara I. Neck dissection versus a watch and wait strategy in T1N0 tongue cancers. B-ENT. 2019;15:179-184.

Carta F, Quartu D, Mariani C, Tatti M, Marrosu V, Gioia E, Gerosa C, Zanda JSA, Chuchueva N, Figus A, Puxeddu R. Compartmental Surgery With Microvascular Free Flap Reconstruction in Patients With T1-T4 Squamous Cell Carcinoma of the Tongue: Analysis of Risk Factors, and Prognostic Value of the 8th Edition AJCC TNM Staging System. Front Oncol. 2020 Jul 14;10:984. doi: 10.3389/fonc.2020.00984. PMID: 32760667; PMCID: PMC7372302.

Ch'ng S, Corbett-Burns S, Stanton N, Gao K, Shannon K, Clifford A, Gupta R, Clark JR. Close margin alone does not warrant postoperative adjuvant radiotherapy in oral squamous cell carcinoma. Cancer. 2013 Jul 1;119(13):2427-37. doi: 10.1002/cncr.28081. Epub 2013 Apr 10. PMID: 23576156.

Chang YC, Nieh S, Chen SF, Jao SW, Lin YL, Fu E. Invasive pattern grading score designed as an independent prognostic indicator in oral squamous cell carcinoma. Histopathology. 2010 Aug;57(2):295-303. doi: 10.1111/j.1365-2559.2010.03616.x. Epub 2010 Jul 26. PMID: 20659175.

Chatterjee D, Bansal V, Malik V, Bhagat R, Punia RS, Handa U, Gupta A, Dass A. Tumor Budding and Worse Pattern of Invasion Can Predict Nodal Metastasis in Oral Cancers and Associated With Poor Survival in Early-Stage Tumors. Ear Nose Throat J. 2019 Aug;98(7):E112-E119. doi: 10.1177/0145561319848669. Epub 2019 May 9. PMID: 31072197.

Chaturvedi P, Vaishampayan SS, Nair S, Nair D, Agarwal JP, Kane SV, Pawar P, Datta S. Oral squamous cell carcinoma arising in background of oral submucous fibrosis: a clinicopathologically distinct disease. Head Neck. 2013 Oct;35(10):1404-9. doi: 10.1002/hed.23143. Epub 2012 Sep 13. PMID: 22972608.

Chatzistamou I, Rodriguez J, Jouffroy T, Girod A, Point D, Sklavounou A, Kittas C, Sastre-Garau X, Klijanienko J. Prognostic significance of tumor shape and stromal chronic inflammatory infiltration in squamous cell carcinomas of the oral tongue. J Oral Pathol Med. 2010 Oct;39(9):667-71. doi: 10.1111/j.1600-0714.2010.00911.x. Epub 2010 Jul 2. PMID: 20618607.

Chen JT, Chen CH, Ku KL, Hsiao M, Chiang CP, Hsu TL, Chen MH, Wong CH. Glycoprotein B7-H3 overexpression and aberrant glycosylation in oral cancer and immune response. Proc Natl Acad Sci U S A. 2015 Oct 20;112(42):13057-62. doi: 10.1073/pnas.1516991112. Epub 2015 Oct 5. PMID: 26438868; PMCID: PMC4620862.

Chen YK, Huang HC, Lin LM, Lin CC. Primary oral squamous cell carcinoma: an analysis of 703 cases in southern Taiwan. Oral Oncol. 1999 Mar;35(2):173-9. doi: 10.1016/s1368-8375(98)00101-8. PMID: 10435152.

Chen YW, Kao SY, Wang HJ, Yang MH. Histone modification patterns correlate with patient outcome in oral squamous cell carcinoma. Cancer. 2013 Dec 15;119(24):4259-67. doi: 10.1002/cncr.28356. Epub 2013 Sep 24. PMID: 24301303.

Chen YW, Yu EH, Wu TH, Lo WL, Li WY, Kao SY. Histopathological factors affecting nodal metastasis in tongue cancer: analysis of 94 patients in Taiwan. Int J Oral Maxillofac Surg. 2008 Oct;37(10):912-6. doi: 10.1016/j.ijom.2008.07.014. Epub 2008 Sep 11. PMID: 18789650.

Chinn SB, Spector ME, Bellile EL, Rozek LS, Lin T, Teknos TN, Prince ME, Bradford CR, Urba SG, Carey TE, Eisbruch A, Wolf GT, Worden FP, Chepeha DB. Efficacy of induction selection chemotherapy vs primary surgery for patients with advanced oral cavity carcinoma. JAMA Otolaryngol Head Neck Surg. 2014 Feb;140(2):134-42. doi: 10.1001/jamaoto.2013.5892. PMID: 24370563; PMCID: PMC4103099.

Chuang ST, Chen CC, Yang SF, Chan LP, Kao YH, Huang MY, Tang JY, Huang CM, Huang CJ. Tumor histologic grade as a risk factor for neck recurrence in patients with T1-2N0 early tongue cancer. Oral Oncol. 2020 Jul;106:104706. doi: 10.1016/j.oraloncology.2020.104706. Epub 2020 Apr 21. PMID: 32330684.

Clark JR, Naranjo N, Franklin JH, de Almeida J, Gullane PJ. Established prognostic variables in N0 oral carcinoma. Otolaryngol Head Neck Surg. 2006 Nov;135(5):748-53. doi: 10.1016/j.otohns.2006.05.751. PMID: 17071306.

De Silva RK, Siriwardena BSMS, Samaranayaka A, Abeyasinghe WAMUL, Tilakaratne WM. A model to predict nodal metastasis in patients with oral squamous cell carcinoma. PLoS One. 2018 Aug 9;13(8):e0201755. doi: 10.1371/journal.pone.0201755. PMID: 30091996; PMCID: PMC6084951.

Dik EA, Ipenburg NA, Kessler PA, van Es RJJ, Willems SM. The value of histological grading of biopsy and resection specimens in early stage oral squamous cell carcinomas. J Craniomaxillofac Surg. 2018 Jun;46(6):1001-1006. doi: 10.1016/j.jcms.2018.03.019. Epub 2018 Apr 5. PMID: 29709328.

Dik EA, Willems SM, Ipenburg NA, Adriaansens SO, Rosenberg AJ, van Es RJ. Resection of early oral squamous cell carcinoma with positive or close margins: relevance of adjuvant treatment in relation to local recurrence: margins of 3 mm as safe as 5 mm. Oral Oncol. 2014 Jun;50(6):611-5. doi: 10.1016/j.oraloncology.2014.02.014. Epub 2014 Mar 14. PMID: 24630900.

Dirven R, Ebrahimi A, Moeckelmann N, Palme CE, Gupta R, Clark J. Tumor thickness versus depth of invasion - Analysis of the 8th edition American Joint Committee on Cancer Staging for oral cancer. Oral Oncol. 2017 Nov;74:30-33. doi: 10.1016/j.oraloncology.2017.09.007. Epub 2017 Sep 19. PMID: 29103748.

Dissanayaka WL, Pitiyage G, Kumarasiri PV, Liyanage RL, Dias KD, Tilakaratne WM. Clinical and histopathologic parameters in survival of oral squamous cell carcinoma. Oral Surg Oral Med Oral Pathol Oral Radiol. 2012 Apr;113(4):518-25. doi: 10.1016/j.oooo.2011.11.001. PMID: 22668430.

Elseragy A, Salo T, Coletta RD, Kowalski LP, Haglund C, Nieminen P, Mäkitie AA, Leivo I, Almangush A. A Proposal to Revise the Histopathologic Grading System of Early Oral Tongue Cancer Incorporating Tumor Budding. Am J Surg Pathol. 2019 May;43(5):703-709. doi: 10.1097/PAS.0000000000001241. PMID: 30829728.

Estilo CL, O-charoenrat P, Talbot S, Socci ND, Carlson DL, Ghossein R, Williams T, Yonekawa Y, Ramanathan Y, Boyle JO, Kraus DH, Patel S, Shaha AR, Wong RJ, Huryn JM, Shah JP, Singh B. Oral tongue cancer gene expression profiling: Identification of novel potential prognosticators by oligonucleotide microarray analysis. BMC Cancer. 2009 Jan 12;9:11. doi: 10.1186/1471-2407-9-11. PMID: 19138406; PMCID: PMC2649155.

Fukano H, Matsuura H, Hasegawa Y, Nakamura S. Depth of invasion as a predictive factor for cervical lymph node metastasis in tongue carcinoma. Head Neck. 1997 May;19(3):205-10. doi: 10.1002/(sici)1097-0347(199705)19:3<205::aid-hed7>3.0.co;2-6. PMID: 9142520.

Garzino-Demo P, Dell'Acqua A, Dalmasso P, Fasolis M, La Terra Maggiore GM, Ramieri G, Berrone S, Rampino M, Schena M. Clinicopathological parameters and outcome of 245 patients operated for oral squamous cell carcinoma. J Craniomaxillofac Surg. 2006 Sep;34(6):344-50. doi: 10.1016/j.jcms.2006.04.004. Epub 2006 Jul 21. PMID: 16859913.

Garzino-Demo P, Zavattero E, Franco P, Fasolis M, Tanteri G, Mettus A, Tosco P, Chiusa L, Airoldi M, Ostellino O, Schena M, Rampino M, Ricardi U, Evangelista A, Merletti F, Berrone S, Ramieri G. Parameters and outcomes in 525 patients operated on for oral squamous cell carcinoma. J Craniomaxillofac Surg. 2016 Sep;44(9):1414-21. doi: 10.1016/j.jcms.2016.06.007. Epub 2016 Jun 15. PMID: 27485718.

Ghafari R, Jalayer Naderi N, Emami Razavi A. A retrospective institutional study of histopathologic pattern of Oral Squamous Cell Carcinoma (OSCC) in Tehran, Iran during 2006-2015. J Res Med Sci. 2019 Jun 25;24:53. doi: 10.4103/jrms.JRMS_882_18. PMID: 31333732; PMCID: PMC6611183.

Goldstein DP, Bachar GY, Lea J, Shrime MG, Patel RS, Gullane PJ, Brown DH, Gilbert RW, Kim J, Waldron J, Perez-Ordonez B, Davis AM, Cheng L, Xu W, Irish JC. Outcomes of squamous cell cancer of the oral tongue managed at the Princess Margaret Hospital. Head Neck. 2013 May;35(5):632-41. doi: 10.1002/hed.23001. Epub 2012 Apr 27. PMID: 22544679.

Gomez R, el-Naggar AK, Byers RM, Garnsey L, Luna MA, Batsakis JG. Squamous carcinoma of oral tongue: prognostic significance of flow-cytometric DNA content. Mod Pathol. 1992 Mar;5(2):141-5. PMID: 1574491.

González-García R, Naval-Gías L, Rodríguez-Campo FJ, Sastre-Pérez J, Muñoz-Guerra MF, Díaz-González FJ. Metástasis cervical contralateral en el carcinoma epidermoide de la cavidad oral. Estudio clínico analítico retrospectivo en 315 pacientes primariamente tratados con cirugía. Rev Esp Cir Oral y Maxilofac. 2008 May-Jun; 30(3):157-171.

González-García R, Naval-Gías L, Román-Romero L, Sastre-Pérez J, Rodríguez-Campo FJ. Local recurrences and second primary tumors from squamous cell carcinoma of the oral cavity: a retrospective analytic study of 500 patients. Head Neck. 2009 Sep;31(9):1168-80. doi: 10.1002/hed.21088. PMID: 19408289.

Gonzalez-Moles MA, Esteban F, Rodriguez-Archilla A, Ruiz-Avila I, Gonzalez-Moles S. Importance of tumour thickness measurement in prognosis of tongue cancer. Oral Oncol. 2002 Jun;38(4):394-7. doi: 10.1016/s1368-8375(01)00081-1. PMID: 12076706.

Guiney A. Tumour parameters determining regional neck node metastasis in squamous cell carcinoma of the oral tongue. Australian Journal of Otolaryngology. 1995;2(2):142-146.

Gupta S, Kamboj M, Narwal A. Knowing the unknown in oral squamous cell carcinoma: An observational study. J Cancer Res Ther. 2020 Apr-Jun;16(3):494-499. doi: 10.4103/jcrt.JCRT_898_18. PMID: 32719256.

Hakeem AH, Pradhan SA, Kannan R, Tubachi J. Clinical outcome of surgical treatment of T1-2 N0 squamous cell carcinoma of oral tongue with observation for the neck: Analysis of 176 cases. Ann Maxillofac Surg. 2016 Jul-Dec;6(2):235-240. doi: 10.4103/2231-0746.200331. PMID: 28299264; PMCID: PMC5343634.

Hasegawa O, Satomi T, Kono M, Watanabe M, Ikehata N, Chikazu D. Correlation between the malignancy and prognosis of oral squamous cell carcinoma in the maximum standardized uptake value. Odontology. 2019 Apr;107(2):237-243. doi: 10.1007/s10266-018-0379-9. Epub 2018 Jul 23. PMID: 30039234.

Hasegawa T, Iga T, Takeda D, Amano R, Saito I, Kakei Y, Kusumoto J, Kimoto A, Sakakibara A, Akashi M. Neutrophil-lymphocyte ratio associated with poor prognosis in oral cancer: a retrospective study. BMC Cancer. 2020 Jun 17;20(1):568. doi: 10.1186/s12885-020-07063-1. PMID: 32552873; PMCID: PMC7302163.

Hilly O, Shkedy Y, Hod R, Soudry E, Mizrachi A, Hamzany Y, Bachar G, Shpitzer T. Carcinoma of the oral tongue in patients younger than 30 years: comparison with patients older than 60 years. Oral Oncol. 2013 Oct;49(10):987-90. doi: 10.1016/j.oraloncology.2013.07.005. Epub 2013 Aug 6. PMID: 23927849.

Hoşal AS, Unal OF, Ayhan A. Possible prognostic value of histopathologic parameters in patients with carcinoma of the oral tongue. Eur Arch Otorhinolaryngol. 1998;255(4):216-9. doi: 10.1007/s004050050046. PMID: 9592681.

Hosni A, Huang SH, Xu W, Su J, Bayley A, Bratman SV, Cho J, Giuliani M, Kim J, Ringash J, Waldron J, Spreafico A, De Almeidad J, O'Sullivan B, Goldstein D, Hope A. Distant Metastases Following Postoperative Intensity-Modulated Radiotherapy for Oral Cavity Squamous Cell Carcinoma. JAMA Otolaryngol Head Neck Surg. 2017 Apr 1;143(4):368-375. doi: 10.1001/jamaoto.2016.3668. PMID: 28033442.

Howaldt HP, Kainz M, Euler B, Vorast H. Proposal for modification of the TNM staging classification for cancer of the oral cavity. DOSAK. J Craniomaxillofac Surg. 1999 Oct;27(5):275-88. doi: 10.1054/jcms.1999.0070. PMID: 10717829.

Huang C, Zhuang SM, Li JJ, Chen SW, Zhang XW, Song M. Can we identify the patients with clinically T1-2N0 oral tongue squamous cell carcinoma benefiting from neck dissection? Int J Clin Exp Med 2017;10(2):4023-4034.

Huang SF, Wei FC, Liao CT, Wang HM, Lin CY, Lo S, Huang JJ, Chen IH, Kang CJ, Chien HT, Chen HH. Risk stratification in oral cavity squamous cell carcinoma by preoperative CRP and SCC antigen levels. Ann Surg Oncol. 2012 Nov;19(12):3856-64. doi: 10.1245/s10434-012-2392-5. Epub 2012 May 11. PMID: 22576068.

Imai T, Satoh I, Matsumoto K, Asada Y, Yamazaki T, Morita S, Saijo S, Okubo JI, Wakamori S, Saijo S, Matsuura K. Retrospective observational study of occult cervical lymph-node metastasis in T1N0 tongue cancer. Jpn J Clin Oncol. 2017 Feb 26;47(2):130-136. doi: 10.1093/jjco/hyw172. PMID: 28175327.

Imayama N, Yamada S, Yanamoto S, Naruse T, Matsushita Y, Takahashi H, Seki S, Fujita S, Ikeda T, Umeda M. FOXC2 expression is associated with tumor proliferation and invasion potential in oral tongue squamous cell carcinoma. Pathol Oncol Res. 2015 Jul;21(3):783-91. doi: 10.1007/s12253-014-9891-6. Epub 2015 Jan 9. PMID: 25573594.

Jain D, Tikku G, Bhadana P, Dravid C, Grover RK. A semi-quantitative World Health Organization grading scheme evaluating worst tumor differentiation predicts disease-free survival in oral squamous carcinoma patients. Ann Diagn Pathol. 2017 Aug;29:1-6. doi: 10.1016/j.anndiagpath.2017.04.003. Epub 2017 Apr 8. PMID: 28807334.

Jani K, Balasubramanian D, Jayasankaran S, Murthy S, Vidyadaran S, Thankappan K, Iyer S. Patterns of growth of lingual carcinoma on magnetic resonance imaging and correlations with clinicopathologic outcomes. Oral Surg Oral Med Oral Pathol Oral Radiol. 2020 Dec;130(6):731-740. doi: 10.1016/j.oooo.2020.06.012. Epub 2020 Jun 20. PMID: 32693950.

Jardim JF, Gondak R, Galvis MM, Pinto CAL, Kowalski LP. A decreased peritumoral CD1a+ cell number predicts a worse prognosis in oral squamous cell carcinoma. Histopathology. 2018 May;72(6):905-913. doi: 10.1111/his.13415. Epub 2018 Feb 6. PMID: 29023924.

Jensen DH, Dabelsteen E, Specht L, Fiehn AM, Therkildsen MH, Jønson L, Vikesaa J, Nielsen FC, von Buchwald C. Molecular profiling of tumour budding implicates TGFβ-mediated epithelial-mesenchymal transition as a therapeutic target in oral squamous cell carcinoma. J Pathol. 2015 Aug;236(4):505-16. doi: 10.1002/path.4550. Epub 2015 Jun 1. PMID: 25925492.

Jiang Q, Tang A, Long S, Qi Q, Song C, Xin Y, Zhang C, Cao Z, Zhang J. Development and validation of a nomogram to predict the risk of occult cervical lymph node metastases in cN0 squamous cell carcinoma of the tongue. Br J Oral Maxillofac Surg. 2019 Dec;57(10):1092-1097. doi: 10.1016/j.bjoms.2019.09.024. Epub 2019 Oct 31. PMID: 31677799.

Jing Y, Jin Y, Wang Y, Chen S, Zhang X, Song Y, Wang Z, Pu Y, Ni Y, Hu Q. SPARC promotes the proliferation and metastasis of oral squamous cell carcinoma by PI3K/AKT/PDGFB/PDGFRβ axis. J Cell Physiol. 2019 Jan 31. doi: 10.1002/jcp.28205. Epub ahead of print. PMID: 30706473.

Jung J, Cho NH, Kim J, Choi EC, Lee SY, Byeon HK, Park YM, Yang WS, Kim SH. Significant invasion depth of early oral tongue cancer originated from the lateral border to predict regional metastases and prognosis. Int J Oral Maxillofac Surg. 2009 Jun;38(6):653-60. doi: 10.1016/j.ijom.2009.01.004. Epub 2009 Feb 23. PMID: 19231137.

Kane SV, Gupta M, Kakade AC, D' Cruz A. Depth of invasion is the most significant histological predictor of subclinical cervical lymph node metastasis in early squamous carcinomas of the oral cavity. Eur J Surg Oncol. 2006 Sep;32(7):795-803. doi: 10.1016/j.ejso.2006.05.004. Epub 2006 Jun 13. PMID: 16777368.

Kao HK, Abdelrahman M, Huang Y, Tsai CH, Barrera MJ, Tsang NM, Couves AJ, Cheng MH, Chang KP. Multiple concomitant oral cavity cancers: Incidence, management, and outcomes. J Surg Oncol. 2017 Jun;115(7):835-841. doi: 10.1002/jso.24600. Epub 2017 Mar 20. PMID: 28320044.

Kato K, Miyazawa H, Kobayashi H, Noguchi N, Lambert D, Kawashiri S. Caveolin-1 Expression at Metastatic Lymph Nodes Predicts Unfavorable Outcome in Patients with Oral Squamous Cell Carcinoma. Pathol Oncol Res. 2020 Oct;26(4):2105-2113. doi: 10.1007/s12253-019-00791-1. Epub 2020 Jan 6. PMID: 31907776.

Katz O, Nachalon Y, Hilly O, Shpitzer T, Bachar G, Limon D, Popovtzer A. Radiotherapy in early-stage tongue squamous cell carcinoma with minor adverse features. Head Neck. 2017 Jan;39(1):147-150. doi: 10.1002/hed.24555. Epub 2016 Aug 10. PMID: 27507221.

Keski-Säntti H, Atula T, Tikka J, Hollmén J, Mäkitie AA, Leivo I. Predictive value of histopathologic parameters in early squamous cell carcinoma of oral tongue. Oral Oncol. 2007 Nov;43(10):1007-13. doi: 10.1016/j.oraloncology.2006.11.015. Epub 2007 Feb 15. PMID: 17306608.

Khwaja T, Tayaar AS, Acharya S, Bhushan J, Muddapur MV. Pattern of invasion as a factor in determining lymph node metastasis in oral squamous cell carcinoma. J Cancer Res Ther. 2018 Jan-Mar;14(2):382-387. doi: 10.4103/0973-1482.187281. PMID: 29516924.

Kim RY, Helman JI, Braun TM, Ward BB. Increased Presence of Perineural Invasion in the Tongue and Floor of the Mouth: Could It Represent a More Aggressive Oral Squamous Cell Carcinoma, or Do Larger Aggressive Tumors Cause Perineural Invasion? J Oral Maxillofac Surg. 2019 Apr;77(4):852-858. doi: 10.1016/j.joms.2018.07.023. Epub 2018 Jul 27. PMID: 30142323.

Klotch DW, Muro-Cacho C, Gal TJ. Factors affecting survival for floor-of-mouth carcinoma. Otolaryngol Head Neck Surg. 2000 Apr;122(4):495-8. doi: 10.1067/mhn.2000.102185. PMID: 10740167.

Kolokythas A, Park S, Schlieve T, Pytynia K, Cox D. Squamous cell carcinoma of the oral tongue: histopathological parameters associated with outcome. Int J Oral Maxillofac Surg. 2015 Sep;44(9):1069-74. doi: 10.1016/j.ijom.2015.01.027. Epub 2015 Jun 6. PMID: 26055524.

Kowalski LP, Bagietto R, Lara JR, Santos RL, Silva JF Jr, Magrin J. Prognostic significance of the distribution of neck node metastasis from oral carcinoma. Head Neck. 2000 May;22(3):207-14. doi: 10.1002/(sici)1097-0347(200005)22:3<207::aid-hed1>3.0.co;2-9. PMID: 10748442.

Książek M, Lewandowski B, Brodowski R, Pakla P, Kawalec-Książek M, Fudali L, Ryś J. The prognostic significance of tumour infiltrating lymphocytes in oral squamous cell carcinoma. Pol J Pathol. 2019;70(4):277-285. doi: 10.5114/pjp.2019.93130. PMID: 32146797.

Kurita H, Koike T, Narikawa JN, Sakai H, Nakatsuka A, Uehara S, Kobayashi H, Kurashina K. Clinical predictors for contralateral neck lymph node metastasis from unilateral squamous cell carcinoma in the oral cavity. Oral Oncol. 2004 Oct;40(9):898-903. doi: 10.1016/j.oraloncology.2004.04.004. PMID: 15380167.

Kurokawa H, Yamashita Y, Murata T, Yoshikawa T, Tokudome S, Miura K, Kajiyama M. Histological grading of malignancy correlates with regional lymph node metastasis and survival of patients with oral squamous cell carcinoma. Fukuoka Igaku Zasshi. 1998 Aug;89(8):225-31. PMID: 9778914.

Kurokawa H, Yamashita Y, Takeda S, Zhang M, Fukuyama H, Takahashi T. Risk factors for late cervical lymph node metastases in patients with stage I or II carcinoma of the tongue. Head Neck. 2002 Aug;24(8):731-6. doi: 10.1002/hed.10130. PMID: 12203797.

Kurokawa H, Zhang M, Matsumoto S, Yamashita Y, Tanaka T, Takamori K, Igawa K, Yoshida M, Fukuyama H, Takahashi T, Sakoda S. Reduced syndecan-1 expression is correlated with the histological grade of malignancy at the deep invasive front in oral squamous cell carcinoma. J Oral Pathol Med. 2006 May;35(5):301-6. doi: 10.1111/j.1600-0714.2006.00412.x. PMID: 16630294.

Kurokawa H, Zhang M, Yamashita Y, Matsumoto S, Takano H, Funaki K, Tomoyose T, Shibuya T, Fukuyama H, Takahashi. Risk factors for postoperative local recurrence of tongue carcinoma. Asian Journal of Oral and Maxillofacial surgery. 2004 Jun;16(2):91-96. Doi: 10.1016/S0915-6992(04)80015-3.

Larson AR, Kemmer J, Formeister E, El-Sayed I, Ha P, George J, Ryan W, Chan E, Heaton C. Beyond Depth of Invasion: Adverse Pathologic Tumor Features in Early Oral Tongue Squamous Cell Carcinoma. Laryngoscope. 2020 Jul;130(7):1715-1720. doi: 10.1002/lary.28241. Epub 2019 Aug 14. PMID: 31411752.

Laske RD, Scholz I, Ikenberg K, Meerwein C, Vital DG, Studer G, Rössle M, Huber GF. Perineural Invasion in Squamous Cell Carcinoma of the Oral Cavity: Histology, Tumor Stage, and Outcome. Laryngoscope Investig Otolaryngol. 2016 Jan 14;1(1):13-18. doi: 10.1002/lio2.4. PMID: 28894798; PMCID: PMC5580858.

Lee TL, Chiu PH, Li WY, Yang MH, Wei PY, Chu PY, Wang YF, Tai SK. Nerve-tumour interaction enhances the aggressiveness of oral squamous cell carcinoma. Clin Otolaryngol. 2019 Nov;44(6):1087-1095. doi: 10.1111/coa.13452. Epub 2019 Nov 4. PMID: 31574203.

Li Z, Wang Y, Zhu Y, Yuan C, Wang D, Zhang W, Qi B, Qiu J, Song X, Ye J, Wu H, Jiang H, Liu L, Zhang Y, Song LN, Yang J, Cheng J. The Hippo transducer TAZ promotes epithelial to mesenchymal transition and cancer stem cell maintenance in oral cancer. Mol Oncol. 2015 Jun;9(6):1091-105. doi: 10.1016/j.molonc.2015.01.007. Epub 2015 Feb 9. PMID: 25704916; PMCID: PMC5528756.

Liao L, Wang J, Ouyang S, Zhang P, Wang J, Zhang M. Expression and clinical significance of microRNA-1246 in human oral squamous cell carcinoma. Med Sci Monit. 2015 Mar 16;21:776-81. doi: 10.12659/MSM.892508. PMID: 25791131; PMCID: PMC4371709.

Libório TN, Ferreira EN, Aquino Xavier FC, Carraro DM, Kowalski LP, Soares FA, Nunes FD. TGIF1 splicing variant 8 is overexpressed in oral squamous cell carcinoma and is related to pathologic and clinical behavior. Oral Surg Oral Med Oral Pathol Oral Radiol. 2013 Nov;116(5):614-25. doi: 10.1016/j.oooo.2013.07.014. PMID: 24119525.

Lim SC, Zhang S, Ishii G, Endoh Y, Kodama K, Miyamoto S, Hayashi R, Ebihara S, Cho JS, Ochiai A. Predictive markers for late cervical metastasis in stage I and II invasive squamous cell carcinoma of the oral tongue. Clin Cancer Res. 2004 Jan 1;10(1 Pt 1):166-72. doi: 10.1158/1078-0432.ccr-0533-3. PMID: 14734465.

Lin WH, Chen IH, Wei FC, Huang JJ, Kang CJ, Hsieh LL, Wang HM, Huang SF. Clinical significance of preoperative squamous cell carcinoma antigen in oral-cavity squamous cell carcinoma. Laryngoscope. 2011 May;121(5):971-7. doi: 10.1002/lary.21721. PMID: 21520110.

Liu Z, Fang Z, Dai T, Zhang C, Sun J, He Y. Higher positive lymph node ratio indicates poorer distant metastasis-free survival in adenoid cystic carcinoma patients with nodal involvement. J Craniomaxillofac Surg. 2015 Jul;43(6):751-7. doi: 10.1016/j.jcms.2015.03.040. Epub 2015 Apr 11. PMID: 25958766.

López-Cedrún JL, Andrés de Llano J. A 22 years survival and prognostic factors analysis in a homogeneous series of 64 patients with advanced cancer of the tongue and the floor of the mouth. J Craniomaxillofac Surg. 2015 Apr;43(3):376-81. doi: 10.1016/j.jcms.2015.01.007. Epub 2015 Jan 24. PMID: 25703504.

Low TH, Gao K, Gupta R, Clifford A, Elliott M, Ch'ng S, Milross C, Clark JR. Factors predicting poor outcomes in T1N0 oral squamous cell carcinoma: indicators for treatment intensification. ANZ J Surg. 2016 May;86(5):366-71. doi: 10.1111/ans.13504. Epub 2016 Mar 17. PMID: 26991038.

Lu C, Lewis JS Jr, Dupont WD, Plummer WD Jr, Janowczyk A, Madabhushi A. An oral cavity squamous cell carcinoma quantitative histomorphometric-based image classifier of nuclear morphology can risk stratify patients for disease-specific survival. Mod Pathol. 2017 Dec;30(12):1655-1665. doi: 10.1038/modpathol.2017.98. Epub 2017 Aug 4. PMID: 28776575; PMCID: PMC6128166.

Lu Z, Yan W, Liang J, Yu M, Liu J, Hao J, Wan Q, Liu J, Luo C, Chen Y. Nomogram Based on Systemic Immune-Inflammation Index to Predict Survival of Tongue Cancer Patients Who Underwent Cervical Dissection. Front Oncol. 2020 Mar 11;10:341. doi: 10.3389/fonc.2020.00341. PMID: 32219070; PMCID: PMC7078378.

Lundqvist L, Stenlund H, Laurell G, Nylander K. The importance of stromal inflammation in squamous cell carcinoma of the tongue. J Oral Pathol Med. 2012 May;41(5):379-83. doi: 10.1111/j.1600-0714.2011.01107.x. Epub 2011 Nov 16. PMID: 22084865.

Ma'aita JK. Oral cancer in Jordan: a retrospective study of 118 patients. Croat Med J. 2000 Mar;41(1):64-9. PMID: 10810170.

Maddox WA, Urist MM. Histopathological prognostic factors of certain primary oral cavity cancers. Oncology (Williston Park). 1990 Dec;4(12):39-42; discussion 42, 45-6. PMID: 2149038.

Majumdar B, Anil S, Sarode SC, Sarode GS, Rao RS, Patil S. Tumor associated tissue eosinophilia as a potential predictor in the invasion patterns of oral squamous cell carcinoma. Journal of International Oral Health. 2016; 8(11):1026-1030. Doi: 10.2047/jioh-08-11-07.

Mamic M, Lucijanic M, Manojlovic L, Muller D, Suton P, Luksic I. Prognostic significance of extranodal extension in oral cavity squamous cell carcinoma with occult neck metastases. Int J Oral Maxillofac Surg. 2021 Mar;50(3):309-315. doi: 10.1016/j.ijom.2020.07.006. Epub 2020 Jul 23. PMID: 32713777.

Mani C, Lakshminarayana G, Kurian A, Annapurneshwari. Predictors of recurrence in early stage oral tongue squamous cell carcinoma. J Orofac Sci 2015;7:86-9. Doi: 10.4103/0975-8844.169753

Manjula M, Angadi PV, Priya NK, Hallikerimath S, Kale AD. Assessment of morphological parameters associated with neural invasion in oral squamous cell carcinoma. J Oral Maxillofac Pathol. 2019 Jan-Apr;23(1):157. doi: 10.4103/jomfp.JOMFP_178_18. PMID: 31110438; PMCID: PMC6503792.

Martínez-Gimeno C, Rodríguez EM, Vila CN, Varela CL. Squamous cell carcinoma of the oral cavity: a clinicopathologic scoring system for evaluating risk of cervical lymph node metastasis. Laryngoscope. 1995 Jul;105(7 Pt 1):728-33. doi: 10.1288/00005537-199507000-00011. PMID: 7603278.

Matos FR, Lima Ed, Queiroz LM, da Silveira EJ. Analysis of inflammatory infiltrate, perineural invasion, and risk score can indicate concurrent metastasis in squamous cell carcinoma of the tongue. J Oral Maxillofac Surg. 2012 Jul;70(7):1703-10. doi: 10.1016/j.joms.2011.08.023. Epub 2011 Dec 10. PMID: 22154400.

Matsui T, Shigeta T, Umeda M, Komori T. Vascular endothelial growth factor C (VEGF-C) expression predicts metastasis in tongue cancer. Oral Surg Oral Med Oral Pathol Oral Radiol. 2015 Oct;120(4):436-42. doi: 10.1016/j.oooo.2015.06.002. Epub 2015 Jun 15. PMID: 26216169.

Matsushita Y, Yanamoto S, Takahashi H, Yamada S, Naruse T, Sakamoto Y, Ikeda H, Shiraishi T, Fujita S, Ikeda T, Asahina I, Umeda M. A clinicopathological study of perineural invasion and vascular invasion in oral tongue squamous cell carcinoma. Int J Oral Maxillofac Surg. 2015 May;44(5):543-8. doi: 10.1016/j.ijom.2015.01.018. Epub 2015 Feb 17. PMID: 25697063.

Matsuura K, Hirokawa Y, Fujita M, Akagi Y, Ito K. Treatment results of stage I and II oral tongue cancer with interstitial brachytherapy: maximum tumor thickness is prognostic of nodal metastasis. Int J Radiat Oncol Biol Phys. 1998 Feb 1;40(3):535-9. doi: 10.1016/s0360-3016(97)00811-0. PMID: 9486601.

Matsuzaki Y, Watabe Y, Enatsu K, Shigematsu S, Shibahara T. Actinin-4 Expression Predicts Poor Disease-free Survival and Correlates with Delayed Lymph Node Metastasis in Patients with Completely Resected Oral Squamous Cell Carcinoma. Bull Tokyo Dent Coll. 2020 Sep 4;61(3):179-186. doi: 10.2209/tdcpublication.2019-0045. Epub 2020 Aug 14. PMID: 32801264.

Melchers LJ, Schuuring E, van Dijk BA, de Bock GH, Witjes MJ, van der Laan BF, van der Wal JE, Roodenburg JL. Tumour infiltration depth ≥4 mm is an indication for an elective neck dissection in pT1cN0 oral squamous cell carcinoma. Oral Oncol. 2012 Apr;48(4):337-42. doi: 10.1016/j.oraloncology.2011.11.007. Epub 2011 Nov 29. PMID: 22130455.

Mermod M, Bongiovanni M, Petrova TV, Dubikovskaya EA, Simon C, Tolstonog G, Monnier Y. Correlation between podoplanin expression and extracapsular spread in squamous cell carcinoma of the oral cavity using subjective immunoreactivity scores and semiquantitative image analysis. Head Neck. 2017 Jan;39(1):98-108. doi: 10.1002/hed.24537. Epub 2016 Jul 20. PMID: 27437903.

Mermod M, Jourdan EF, Gupta R, Bongiovanni M, Tolstonog G, Simon C, Clark J, Monnier Y. Development and validation of a multivariable prediction model for the identification of occult lymph node metastasis in oral squamous cell carcinoma. Head Neck. 2020 Aug;42(8):1811-1820. doi: 10.1002/hed.26105. Epub 2020 Feb 14. PMID: 32057148.

Michikawa C, Izumo T, Sumino J, Morita T, Ohyama Y, Michi Y, Uzawa N. Small size of metastatic lymph nodes with extracapsular spread greatly impacts treatment outcomes in oral squamous cell carcinoma patients. Int J Oral Maxillofac Surg. 2018 Jul;47(7):830-835. doi: 10.1016/j.ijom.2017.12.007. Epub 2018 Jan 17. PMID: 29373201.

Migueláñez-Medrán BC, Pozo-Kreilinger JJ, Cebrián-Carretero JL, Martínez-García MA, López-Sánchez AF. Oral squamous cell carcinoma of tongue: Histological risk assessment. A pilot study. Med Oral Patol Oral Cir Bucal. 2019 Sep 1;24(5):e603-e609. doi: 10.4317/medoral.23011. PMID: 31422411; PMCID: PMC6764715.

Mishra A, Datta S, Malik A, Garg A, Nair D, Nair S, Mair M, Bal M, Agarwal J, Chaturvedi P. Role of microscopic spread beyond gross disease as an adverse prognostic factor in oral squamous cell carcinoma. Eur J Surg Oncol. 2017 Aug;43(8):1503-1508. doi: 10.1016/j.ejso.2017.04.013. Epub 2017 May 6. PMID: 28528911.

Miyazawa H, Kato K, Kobayashi Y, Hirai M, Kimura I, Kitahara H, Noguchi N, Nakamura H, Kawashiri S. Clinicopathological Significance of the ET Axis in Human Oral Squamous Cell Carcinoma. Pathol Oncol Res. 2019 Jul;25(3):1083-1089. doi: 10.1007/s12253-018-0514-5. Epub 2018 Oct 31. PMID: 30382525; PMCID: PMC6614151.

Monevska DP, Janevska V, Naumovski S, Popovski V, Benedetti A, Bozovich S, Ismani A. Multiple pathohisthological parameters influencing prognosis and survival of oral cancer patients. Pril (Makedon Akad Nauk Umet Odd Med Nauki). 2013;34(2):169-74. PMID: 24280891.

Morand GB, Ikenberg K, Vital DG, Cardona I, Moch H, Stoeckli SJ, Huber GF. Preoperative assessment of CD44-mediated depth of invasion as predictor of occult metastases in early oral squamous cell carcinoma. Head Neck. 2019 Apr;41(4):950-958. doi: 10.1002/hed.25532. Epub 2018 Dec 18. PMID: 30561155.

Morton RP, Ferguson CM, Lambie NK, Whitlock RM. Tumor thickness in early tongue cancer. Arch Otolaryngol Head Neck Surg. 1994 Jul;120(7):717-20. doi: 10.1001/archotol.1994.01880310023005. PMID: 8018323.

Moura IM, Delgado ML, Silva PM, Lopes CA, do Amaral JB, Monteiro LS, Bousbaa H. High CDC20 expression is associated with poor prognosis in oral squamous cell carcinoma. J Oral Pathol Med. 2014 Mar;43(3):225-31. doi: 10.1111/jop.12115. Epub 2013 Sep 16. PMID: 24044615.

Mukoyama N, Suzuki H, Hanai N, Sone M, Hasegawa Y. Pathological tumor volume predicts survival outcomes in oral squamous cell carcinoma. Oncol Lett. 2018 Aug;16(2):2471-2477. doi: 10.3892/ol.2018.8951. Epub 2018 Jun 12. PMID: 30013639; PMCID: PMC6036551.

Muñoz-Guerra MF, Fernández-Contreras ME, Moreno AL, Martín ID, Herráez B, Gamallo C. Polymorphisms in the hypoxia inducible factor 1-alpha and the impact on the prognosis of early stages of oral cancer. Ann Surg Oncol. 2009 Aug;16(8):2351-8. doi: 10.1245/s10434-009-0503-8. Epub 2009 May 16. PMID: 19449077.

Murthy SP, Thankappan K, Jayasankaran SC, Milind K, Prasad C, Balasubramanian D, Iyer S. "Deep Extrinsic Muscle Involvement" Is a Fallacy in the American Joint Committee on Cancer's Seventh Edition of Tumor Staging of Oral Cavity Cancers. J Oral Maxillofac Surg. 2018 Jan;76(1):206-212. doi: 10.1016/j.joms.2017.06.003. Epub 2017 Jun 10. PMID: 28683301.

Myers LL, Sumer BD, Truelson JM, Nedzi L, Perkins S, Hughes RS, Ahn C. Impact of treatment sequence of multimodal therapy for advanced oral cavity cancer with mandible invasion. Otolaryngol Head Neck Surg. 2011 Dec;145(6):961-6. doi: 10.1177/0194599811417550. Epub 2011 Aug 2. PMID: 21810773.

Nadaf A, Bavle RM, Soumya M, D'mello S, Kuriakose MA, Govindan S. Analysis of the invasive edge in primary and secondary oral squamous cell carcinoma: An independent prognostic marker: A retrospective study. J Oral Maxillofac Pathol. 2016 May-Aug;20(2):239-45. doi: 10.4103/0973-029X.185931. PMID: 27601816; PMCID: PMC4989554.

Nagata M, Noman AA, Suzuki K, Kurita H, Ohnishi M, Ohyama T, Kitamura N, Kobayashi T, Uematsu K, Takahashi K, Kodama N, Kawase T, Hoshina H, Ikeda N, Shingaki S, Takagi R. ITGA3 and ITGB4 expression biomarkers estimate the risks of locoregional and hematogenous dissemination of oral squamous cell carcinoma. BMC Cancer. 2013 Sep 5;13:410. doi: 10.1186/1471-2407-13-410. PMID: 24006899; PMCID: PMC3844399.

Nair S, Singh B, Pawar PV, Datta S, Nair D, Kane S, Chaturvedi P. Squamous cell carcinoma of tongue and buccal mucosa: clinico-pathologically different entities. Eur Arch Otorhinolaryngol. 2016 Nov;273(11):3921-3928. doi: 10.1007/s00405-016-4051-0. Epub 2016 Apr 20. PMID: 27098612.

Nakashima H, Matsuoka Y, Yoshida R, Nagata M, Hirosue A, Kawahara K, Sakata J, Arita H, Hiraki A, Nakayama H. Pre-treatment neutrophil to lymphocyte ratio predicts the chemoradiotherapy outcome and survival in patients with oral squamous cell carcinoma: a retrospective study. BMC Cancer. 2016 Jan 26;16:41. doi: 10.1186/s12885-016-2079-6. PMID: 26812901; PMCID: PMC4728793.

Nakayama A, Ogawa A, Fukuta Y, Kudo K. Relation between lymphatic vessel diameter and clinicopathologic parameters in squamous cell carcinomas of the oral region. Cancer. 1999 Jul 15;86(2):200-6. doi: 10.1002/(sici)1097-0142(19990715)86:2<200::aid-cncr3>3.0.co;2-#. PMID: 10421255.

Nandita KP, Boaz K, Srikant N, Lewis AJ, Manaktala N. Tumour budding: A promising parameter in oral squamous cell carcinoma. Research Journal of Pharmaceutical, Biological and Chemical Sciences. 2016 Sep 1;7(5):2059-2063.

Nassiri AM, Campbell BR, Mannion K, Sinard RJ, Netterville JL, Rohde SL. Survival Outcomes in T4aN0M0 Mandibular Gingival Squamous Cell Carcinoma Treated with Surgery Alone. Otolaryngol Head Neck Surg. 2019 May;160(5):870-875. doi: 10.1177/0194599818821892. Epub 2019 Jan 1. PMID: 30598048.

Nathanson A, Agren K, Biörklund A, Lind MG, Andréason L, Anniko M, Freijd A, Lejdeborn L, Kinman S, Kumlien A, et al. Evaluation of some prognostic factors in small squamous cell carcinoma of the mobile tongue: a multicenter study in Sweden. Head Neck. 1989 Sep-Oct;11(5):387-92. doi: 10.1002/hed.2880110502. PMID: 2681065.

Nayanar SK, Tripathy JP, Duraisamy K, Babu S. Prognostic efficiency of clinicopathologic scoring to predict cervical lymph node metastasis in oral squamous cell carcinoma. J Oral Maxillofac Pathol. 2019 Jan-Apr;23(1):36-42. doi: 10.4103/jomfp.JOMFP_132_17. PMID: 31110414; PMCID: PMC6503798.

Nikitakis NG, Sarlani E, Kolokythas A, Scheper MA, Kamperos G, Ord RA, Sklavounou-Andrikopoulou A. Frequency of pain and correlation with clinical and histologic parameters in T1 squamous cell carcinoma of the tongue: a retrospective pilot study. J Oral Facial Pain Headache. 2014 Winter;28(1):46-51. doi: 10.11607/jop.969. PMID: 24482787.

Noble AR, Greskovich JF, Han J, Reddy CA, Nwizu TI, Khan MF, Scharpf J, Adelstein DJ, Burkey BB, Koyfman SA. Risk Factors Associated with Disease Recurrence in Patients with Stage III/IV Squamous Cell Carcinoma of the Oral Cavity Treated with Surgery and Postoperative Radiotherapy. Anticancer Res. 2016 Feb;36(2):785-92. PMID: 26851040.

Nur MM, Al Saadi M, O'Regan EM, Van Harten M, Toner M. Small and Thin Oral Squamous Cell Carcinomas may Exhibit Adverse Pathologic Prognostic Features. Head Neck Pathol. 2021 Jun;15(2):461-468. doi: 10.1007/s12105-020-01218-z. Epub 2020 Sep 12. PMID: 32918712; PMCID: PMC8134593.

O'Brien CJ, Adams JR, McNeil EB, Taylor P, Laniewski P, Clifford A, Parker GD. Influence of bone invasion and extent of mandibular resection on local control of cancers of the oral cavity and oropharynx. Int J Oral Maxillofac Surg. 2003 Oct;32(5):492-7. PMID: 14759107.

O'Brien CJ, Lauer CS, Fredricks S, Clifford AR, McNeil EB, Bagia JS, Koulmandas C. Tumor thickness influences prognosis of T1 and T2 oral cavity cancer--but what thickness? Head Neck. 2003 Nov;25(11):937-45. doi: 10.1002/hed.10324. PMID: 14603454.

O-charoenrat P, Pillai G, Patel S, Fisher C, Archer D, Eccles S, Rhys-Evans P. Tumour thickness predicts cervical nodal metastases and survival in early oral tongue cancer. Oral Oncol. 2003 Jun;39(4):386-90. doi: 10.1016/s1368-8375(02)00142-2. PMID: 12676259.

Odell EW, Jani P, Sherriff M, Ahluwalia SM, Hibbert J, Levison DA, Morgan PR. The prognostic value of individual histologic grading parameters in small lingual squamous cell carcinomas. The importance of the pattern of invasion. Cancer. 1994 Aug 1;74(3):789-94. doi: 10.1002/1097-0142(19940801)74:3<789::aid-cncr2820740302>3.0.co;2-a. PMID: 8039106.

Okada Y, Mataga I, Katagiri M, Ishii K. An analysis of cervical lymph nodes metastasis in oral squamous cell carcinoma. Relationship between grade of histopathological malignancy and lymph nodes metastasis. Int J Oral Maxillofac Surg. 2003 Jun;32(3):284-8. doi: 10.1054/ijom.2002.0303. PMID: 12767876.

Okuyama K, Fukushima H, Naruse T, Yanamoto S, Tsuchihashi H, Umeda M. CD44 Variant 6 Expression and Tumor Budding in the Medullary Invasion Front of Mandibular Gingival Squamous Cell Carcinoma Are Predictive Factors for Cervical Lymph Node Metastasis. Pathol Oncol Res. 2019 Apr;25(2):603-609. doi: 10.1007/s12253-018-0529-y. Epub 2018 Nov 1. PMID: 30387013.

Osaka R, Yamamoto N, Nomura T, Takano N, Shibahara T, Matsuzaka K. Evaluation of infiltrative growth pattern in squamous cell carcinoma of the tongue: comparison with Yamamoto-Kohama classification. Journal of Oral and Maxillofacial Surgery, Medicine, and Pathology. 2015; 27:250-254. Doi: 10.1016/j.ajoms.2014.10.004.

Parsons JT, Mendenhall WM, Stringer SP, Cassisi NJ, Million RR. An analysis of factors influencing the outcome of postoperative irradiation for squamous cell carcinoma of the oral cavity. Int J Radiat Oncol Biol Phys. 1997 Aug 1;39(1):137-48. doi: 10.1016/s0360-3016(97)00152-1. PMID: 9300748.

Patel RS, Dirven R, Clark JR, Swinson BD, Gao K, O'Brien CJ. The prognostic impact of extent of bone invasion and extent of bone resection in oral carcinoma. Laryngoscope. 2008 May;118(5):780-5. doi: 10.1097/MLG.0b013e31816422bb. PMID: 18300706.

Patel SH, Munson ND, Grant DG, Buskirk SJ, Hinni ML, Perry WC, Foote RL, McNeil RB, Halyard MY. Relapse patterns after transoral laser microsurgery and postoperative irradiation for squamous cell carcinomas of the tonsil and tongue base. Ann Otol Rhinol Laryngol. 2014 Jan;123(1):32-9. doi: 10.1177/0003489414521383. PMID: 24574421.

Pedersen NJ, Jensen DH, Lelkaitis G, Kiss K, Charabi B, Specht L, von Buchwald C. Construction of a pathological risk model of occult lymph node metastases for prognostication by semi-automated image analysis of tumor budding in early-stage oral squamous cell carcinoma. Oncotarget. 2017 Mar 14;8(11):18227-18237. doi: 10.18632/oncotarget.15314. PMID: 28212555; PMCID: PMC5392322.

Peixoto TS, Gomes MC, Gomes DQC, Lima KC, Granville-Garcia AF, Costa EMMB. Analysis of survival rates and prognostic factors among patients with oral squamous cell carcinoma. J Public Health. 2017;25:433-441. Doi: 10.1007/s10389-017-0794-3

Petera J, Sirák I, Laco J, Kašaová L, Tuček L, Doležalová H. High-dose-rate brachytherapy in early oral cancer with close or positive margins. Brachytherapy. 2015 Jan-Feb;14(1):77-83. doi: 10.1016/j.brachy.2014.08.050. Epub 2014 Sep 26. PMID: 25264037.

Piazza C, Grammatica A, Montalto N, Paderno A, Del Bon F, Nicolai P. Compartmental surgery for oral tongue and floor of the mouth cancer: Oncologic outcomes. Head Neck. 2019 Jan;41(1):110-115. doi: 10.1002/hed.25480. Epub 2018 Dec 11. PMID: 30536781.

Pollom EL, Chin AL, Lee NY, Tsai CJ. Patterns of Care in Adjuvant Therapy for Resected Oral Cavity Squamous Cell Cancer in Elderly Patients. Int J Radiat Oncol Biol Phys. 2017 Jul 15;98(4):758-766. doi: 10.1016/j.ijrobp.2017.01.224. Epub 2017 Feb 2. PMID: 28366574; PMCID: PMC6240349.

Rahman N, MacNeill M, Wallace W, Conn B. Reframing Histological Risk Assessment of Oral Squamous Cell Carcinoma in the Era of UICC 8th Edition TNM Staging. Head Neck Pathol. 2021 Mar;15(1):202-211. doi: 10.1007/s12105-020-01201-8. Epub 2020 Jul 13. PMID: 32661668; PMCID: PMC8010015.

Rajappa SK, Ram D, Bhakuni YS, Jain A, Kumar R, Dewan AK. Survival benefits of adjuvant radiation in the management of early tongue cancer with depth of invasion as the indication. Head Neck. 2018 Oct;40(10):2263-2270. doi: 10.1002/hed.25329. Epub 2018 Jun 26. PMID: 29947144.

Reddy V, Wadhwan V, Reddy M, Venkatesh A. Controversies on tumor thickness versus nodal metastasis in oral squamous cell carcinomas revealed: A histopathologist's perspective. Indian J Med Paediatr Oncol 2018;39:18-22.

Rodrigues PC, Miguel MC, Bagordakis E, Fonseca FP, de Aquino SN, Santos-Silva AR, Lopes MA, Graner E, Salo T, Kowalski LP, Coletta RD. Clinicopathological prognostic factors of oral tongue squamous cell carcinoma: a retrospective study of 202 cases. Int J Oral Maxillofac Surg. 2014 Jul;43(7):795-801. doi: 10.1016/j.ijom.2014.01.014. Epub 2014 Feb 28. PMID: 24583139.

Roh JL, Cho KJ, Kwon GY, Ryu CH, Chang HW, Choi SH, Nam SY, Kim SY. The prognostic value of hypoxia markers in T2-staged oral tongue cancer. Oral Oncol. 2009 Jan;45(1):63-8. doi: 10.1016/j.oraloncology.2008.03.017. Epub 2008 Jul 11. PMID: 18620902.

Rollo J, Rozenbom CV, Thawley S, Korba A, Ogura J, Perez CA, Powers WE, Bauer WC. Squamous carcinoma of the base of the tongue: a clinicopathologic study of 81 cases. Cancer. 1981 Jan 15;47(2):333-42. doi: 10.1002/1097-0142(19810115)47:2<333::aid-cncr2820470221>3.0.co;2-9. PMID: 7459821.

Routray S, Kheur S, Chougule HM, Mohanty N, Dash R. Establishing Fascin over-expression as a strategic regulator of neoplastic aggression and lymph node metastasis in oral squamous cell carcinoma tumor microenvironment. Ann Diagn Pathol. 2017 Oct;30:36-41. doi: 10.1016/j.anndiagpath.2017.05.013. Epub 2017 May 26. PMID: 28965626.

Rubio Bueno P, Naval Gias L, García Delgado R, Domingo Cebollada J, Díaz González FJ. Tumor DNA content as a prognostic indicator in squamous cell carcinoma of the oral cavity and tongue base. Head Neck. 1998 May;20(3):232-9. doi: 10.1002/(sici)1097-0347(199805)20:3<232::aid-hed8>3.0.co;2-1. PMID: 9570629.

Safi AF, Kauke M, Grandoch A, Nickenig HJ, Drebber U, Zöller J, Kreppel M. Clinicopathological parameters affecting nodal yields in patients with oral squamous cell carcinoma receiving selective neck dissection. J Craniomaxillofac Surg. 2017 Dec;45(12):2092-2096. doi: 10.1016/j.jcms.2017.08.020. Epub 2017 Sep 10. PMID: 29033003.

Sagheb K, Blatt S, Kraft IS, Zimmer S, Rahimi-Nedjat RK, Al-Nawas B, Walter C. Outcome and cervical metastatic spread of squamous cell cancer of the buccal mucosa, a retrospective analysis of the past 25 years. J Oral Pathol Med. 2017 Jul;46(6):460-464. doi: 10.1111/jop.12537. Epub 2017 Jun 6. PMID: 27935122.

Salama AM, Valero C, Katabi N, Khimraj A, Yuan A, Zanoni DK, Ganly I, Patel SG, Ghossein R, Xu B. Depth of invasion versus tumour thickness in early oral tongue squamous cell carcinoma: which measurement is the most practical and predictive of outcome? Histopathology. 2021 Sep;79(3):325-337. doi: 10.1111/his.14291. Epub 2020 Dec 14. PMID: 33112422; PMCID: PMC8079561.

Sandu K, Nisa L, Monnier P, Simon C, Andrejevic-Blant S, Bron L. Clinicobiological progression and prognosis of oral squamous cell carcinoma in relation to the tumor invasive front: impact on prognosis. Acta Otolaryngol. 2014 Apr;134(4):416-24. doi: 10.3109/00016489.2013.849818. PMID: 24628337.

Sarioğlu T, Yilmaz T, Sungur A, Gürsel B. The effect of lymphocytic infiltration on clinical survival in cancer of the tongue. Eur Arch Otorhinolaryngol. 1994;251(6):366-9. doi: 10.1007/BF00171547. PMID: 7848649.

Sasaki T, Moles DR, Imai Y, Speight PM. Clinico-pathological features of squamous cell carcinoma of the oral cavity in patients <40 years of age. J Oral Pathol Med. 2005 Mar;34(3):129-33. doi: 10.1111/j.1600-0714.2004.00291.x. PMID: 15689225.

Sawair FA, Irwin CR, Gordon DJ, Leonard AG, Stephenson M, Napier SS. Invasive front grading: reliability and usefulness in the management of oral squamous cell carcinoma. J Oral Pathol Med. 2003 Jan;32(1):1-9. doi: 10.1034/j.1600-0714.2003.00060.x. PMID: 12558952.

Sawazaki-Calone I, Rangel A, Bueno AG, Morais CF, Nagai HM, Kunz RP, Souza RL, Rutkauskis L, Salo T, Almangush A, Coletta RD. The prognostic value of histopathological grading systems in oral squamous cell carcinomas. Oral Dis. 2015 Sep;21(6):755-61. doi: 10.1111/odi.12343. Epub 2015 Apr 24. PMID: 25825335.

Seki M, Sano T, Yokoo S, Oyama T. Histologic assessment of tumor budding in preoperative biopsies to predict nodal metastasis in squamous cell carcinoma of the tongue and floor of the mouth. Head Neck. 2016 Apr;38 Suppl 1:E1582-90. doi: 10.1002/hed.24282. Epub 2015 Nov 23. PMID: 26595238.

Seki M, Sano T, Yokoo S, Oyama T. Tumour budding evaluated in biopsy specimens is a useful predictor of prognosis in patients with cN0 early stage oral squamous cell carcinoma. Histopathology. 2017 May;70(6):869-879. doi: 10.1111/his.13144. Epub 2017 Feb 16. PMID: 27926795.

Sessions DG, Lenox J, Spector GJ, Chao C, Chaudry OA. Analysis of treatment results for base of tongue cancer. Laryngoscope. 2003 Jul;113(7):1252-61. doi: 10.1097/00005537-200307000-00026. PMID: 12838028.

Shaban M, Khurram SA, Fraz MM, Alsubaie N, Masood I, Mushtaq S, Hassan M, Loya A, Rajpoot NM. A Novel Digital Score for Abundance of Tumour Infiltrating Lymphocytes Predicts Disease Free Survival in Oral Squamous Cell Carcinoma. Sci Rep. 2019 Sep 16;9(1):13341. doi: 10.1038/s41598-019-49710-z. PMID: 31527658; PMCID: PMC6746698.

Shah JP, Cendon RA, Farr HW, Strong EW. Carcinoma of the oral cavity. factors affecting treatment failure at the primary site and neck. Am J Surg. 1976 Oct;132(4):504-7. doi: 10.1016/0002-9610(76)90328-7. PMID: 1015542.

Shan J, Jiang R, Chen X, Zhong Y, Zhang W, Xie L, Cheng J, Jiang H. Machine Learning Predicts Lymph Node Metastasis in Early-Stage Oral Tongue Squamous Cell Carcinoma. J Oral Maxillofac Surg. 2020 Dec;78(12):2208-2218. doi: 10.1016/j.joms.2020.06.015. Epub 2020 Jun 13. PMID: 32649894.

Sharma A, Boaz K, Natarajan S. Understanding patterns of invasion: a novel approach to assessment of podoplanin expression in the prediction of lymph node metastasis in oral squamous cell carcinoma. Histopathology. 2018 Mar;72(4):672-678. doi: 10.1111/his.13416. Epub 2017 Dec 14. PMID: 29023936.

Shaw RJ, Brown JS, Woolgar JA, Lowe D, Rogers SN, Vaughan ED. The influence of the pattern of mandibular invasion on recurrence and survival in oral squamous cell carcinoma. Head Neck. 2004 Oct;26(10):861-9. doi: 10.1002/hed.20036. PMID: 15390204.

Shaw RJ, McGlashan G, Woolgar JA, Lowe D, Brown JS, Vaughan ED, Rogers SN. Prognostic importance of site in squamous cell carcinoma of the buccal mucosa. Br J Oral Maxillofac Surg. 2009 Jul;47(5):356-9. doi: 10.1016/j.bjoms.2008.09.017. Epub 2008 Dec 3. PMID: 19054598.

Sheahan P, O'Keane C, Sheahan JN, O'Dwyer TP. Effect of tumour thickness and other factors on the risk of regional disease and treatment of the N0 neck in early oral squamous carcinoma. Clin Otolaryngol Allied Sci. 2003 Oct;28(5):461-71. doi: 10.1046/j.1365-2273.2003.00748.x. PMID: 12969352.

Shimizu S, Miyazaki A, Sonoda T, Koike K, Ogi K, Kobayashi JI, Kaneko T, Igarashi T, Ueda M, Dehari H, Miyakawa A, Hasegawa T, Hiratsuka H. Tumor budding is an independent prognostic marker in early stage oral squamous cell carcinoma: With special reference to the mode of invasion and worst pattern of invasion. PLoS One. 2018 Apr 19;13(4):e0195451. doi: 10.1371/journal.pone.0195451. PMID: 29672550; PMCID: PMC5909609.

Shinn JR, Wood CB, Colazo JM, Harrell FE Jr, Rohde SL, Mannion K. Cumulative incidence of neck recurrence with increasing depth of invasion. Oral Oncol. 2018 Dec;87:36-42. doi: 10.1016/j.oraloncology.2018.10.015. Epub 2018 Oct 20. PMID: 30527241.

Singh A, Mishra A, Singhvi H, Sharin F, Bal M, Laskar SG, Prabhash K, Chaturvedi P. Optimum surgical margins in squamous cell carcinoma of the oral tongue: Is the current definition adequate? Oral Oncol. 2020 Dec;111:104938. doi: 10.1016/j.oraloncology.2020.104938. Epub 2020 Jul 30. PMID: 32739791.

Sinha N, Rigby MH, McNeil ML, Taylor SM, Trites JR, Hart RD, Bullock MJ. The histologic risk model is a useful and inexpensive tool to assess risk of recurrence and death in stage I or II squamous cell carcinoma of tongue and floor of mouth. Mod Pathol. 2018 May;31(5):772-779. doi: 10.1038/modpathol.2017.183. Epub 2018 Feb 2. PMID: 29393297.

Siow MY, Ng LP, Vincent-Chong VK, Jamaludin M, Abraham MT, Abdul Rahman ZA, Kallarakkal TG, Yang YH, Cheong SC, Zain RB. Dysregulation of miR-31 and miR-375 expression is associated with clinical outcomes in oral carcinoma. Oral Dis. 2014 May;20(4):345-51. doi: 10.1111/odi.12118. Epub 2013 May 7. PMID: 23651447.

Siriwardena BS, Kudo Y, Ogawa I, Udagama MN, Tilakaratne WM, Takata T. VEGF-C is associated with lymphatic status and invasion in oral cancer. J Clin Pathol. 2008 Jan;61(1):103-8. doi: 10.1136/jcp.2007.047662. Epub 2007 Mar 9. PMID: 17351086.

Siriwardena BSMS, Karunathilaka HDNU, Kumarasiri PVR, Tilakaratne WM. Impact of Histological and Molecular Parameters on Prognosis of Oral Squamous Cell Carcinoma: Analysis of 290 Cases. Biomed Res Int. 2020 Oct 14;2020:2059240. doi: 10.1155/2020/2059240. PMID: 33123565; PMCID: PMC7584939.

Siriwardena BSMS, Rambukewela IK, Pitakotuwage TN, Udagama MNGPK, Kumarasiri PVR, Tilakaratne WM. A Predictive Model to Determine the Pattern of Nodal Metastasis in Oral Squamous Cell Carcinoma. Biomed Res Int. 2018 May 13;2018:8925818. doi: 10.1155/2018/8925818. PMID: 29862295; PMCID: PMC5971289.

Sittitrai P, Srivanitchapoom C, Mahanupab P, Pattarasakulchai T, Tananuvat R, Unejanum W. Impact of Clinical and Histo-Pathological Prognostic Factors on T1-2N0-1 Oral Tongue Carcinoma. Indian J Otolaryngol Head Neck Surg. 2013 Jan;65(1):66-70. doi: 10.1007/s12070-012-0605-7. Epub 2012 Dec 2. PMID: 24381924; PMCID: PMC3585553.

Slieker FJB, de Bree R, Van Cann EM. Oral squamous cell carcinoma involving the maxillae: Factors affecting local recurrence and the value of salvage treatment for overall survival. Head Neck. 2020 Aug;42(8):1821-1828. doi: 10.1002/hed.26108. Epub 2020 Feb 26. PMID: 32101351; PMCID: PMC7496535.

Søland TM, Brusevold IJ, Koppang HS, Schenck K, Bryne M. Nerve growth factor receptor (p75 NTR) and pattern of invasion predict poor prognosis in oral squamous cell carcinoma. Histopathology. 2008 Jul;53(1):62-72. doi: 10.1111/j.1365-2559.2008.03063.x. Epub 2008 Jun 6. PMID: 18540978.

Song X, Xia R, Li J, Long Z, Ren H, Chen W, Mao L. Common and complex Notch1 mutations in Chinese oral squamous cell carcinoma. Clin Cancer Res. 2014 Feb 1;20(3):701-10. doi: 10.1158/1078-0432.CCR-13-1050. Epub 2013 Nov 25. PMID: 24277457; PMCID: PMC3946562.

Sopka DM, Li T, Lango MN, Mehra R, Liu JC, Burtness B, Flieder DB, Ridge JA, Galloway TJ. Dysplasia at the margin? Investigating the case for subsequent therapy in 'low-risk' squamous cell carcinoma of the oral tongue. Oral Oncol. 2013 Nov;49(11):1083-7. doi: 10.1016/j.oraloncology.2013.08.001. Epub 2013 Sep 17. PMID: 24054332; PMCID: PMC4037753.

Soudry E, Preis M, Hod R, Hamzany Y, Hadar T, Bahar G, Strenov Y, Shpitzer T. Squamous cell carcinoma of the oral tongue in patients younger than 30 years: clinicopathologic features and outcome. Clin Otolaryngol. 2010 Aug;35(4):307-12. doi: 10.1111/j.1749-4486.2010.02164.x. PMID: 20738340.

Sowmya SV, Rao RS, Prasad K. Development of clinico-histopathological predictive model for the assessment of metastatic risk of oral squamous cell carcinoma. J Carcinog. 2020 May 18;19:2. doi: 10.4103/jcar.JCar_16_19. Erratum in: J Carcinog. 2020 Oct 08;19:10. PMID: 32684850; PMCID: PMC7363157.

Sparano A, Weinstein G, Chalian A, Yodul M, Weber R. Multivariate predictors of occult neck metastasis in early oral tongue cancer. Otolaryngol Head Neck Surg. 2004 Oct;131(4):472-6. doi: 10.1016/j.otohns.2004.04.008. PMID: 15467620.

Spiotto MT, Jefferson GD, Wenig B, Markiewicz MR, Weichselbaum RR, Koshy M. Survival outcomes for postoperative chemoradiation in intermediate-risk oral tongue cancers. Head Neck. 2017 Dec;39(12):2537-2548. doi: 10.1002/hed.24932. Epub 2017 Sep 27. PMID: 28960621.

Spiro RH, Guillamondegui O Jr, Paulino AF, Huvos AG. Pattern of invasion and margin assessment in patients with oral tongue cancer. Head Neck. 1999 Aug;21(5):408-13. doi: 10.1002/(sici)1097-0347(199908)21:5<408::aid-hed5>3.0.co;2-e. PMID: 10402520.

Spiro RH, Huvos AG, Wong GY, Spiro JD, Gnecco CA, Strong EW. Predictive value of tumor thickness in squamous carcinoma confined to the tongue and floor of the mouth. Am J Surg. 1986 Oct;152(4):345-50. doi: 10.1016/0002-9610(86)90302-8. PMID: 3766861.

Subramaniam N, Balasubramanian D, Low TH, Murthy S, Anand A, Prasad C, Vijayan SN, Thankappan K, Iyer S. Role of adverse pathological features in surgically treated early oral cavity carcinomas with adequate margins and the development of a scoring system to predict local control. Head Neck. 2018 Nov;40(11):2329-2333. doi: 10.1002/hed.25163. Epub 2018 Nov 1. PMID: 30381858.

Subramaniam N, Balasubramanian D, Low TH, Murthy S, Clark JR, Thankappan K, Iyer S. Factors Affecting Survival in Surgically Salvaged Locoregional Recurrences of Squamous Cell Carcinoma of the Tongue. J Oral Maxillofac Surg. 2018 May;76(5):1133.e1-1133.e6. doi: 10.1016/j.joms.2017.12.029. Epub 2018 Jan 9. PMID: 29406254.

Subramaniam N, Balasubramanian D, Murthy S, Rathod P, Vidhyadharan S, Thankappan K, Iyer S. Impact of postoperative radiotherapy on survival and loco-regional control in node-negative oral cavity tumours classified as T3 using the AJCC Cancer Staging Manual eighth edition. Int J Oral Maxillofac Surg. 2019 Feb;48(2):152-156. doi: 10.1016/j.ijom.2018.07.009. Epub 2018 Sep 20. PMID: 30243830.

Sudo S, Kajiya H, Okano S, Sasaki M, Katsumata Y, Ohno J, Ikebe T, Hiraki A, Okabe K. Cisplatin-induced programmed cell death ligand-2 expression is associated with metastasis ability in oral squamous cell carcinoma. Cancer Sci. 2020 Apr;111(4):1113-1123. doi: 10.1111/cas.14336. Epub 2020 Feb 29. PMID: 32012401; PMCID: PMC7156784.

Suton P, Salaric I, Granic M, Mueller D, Luksic I. Prognostic significance of extracapsular spread of lymph node metastasis from oral squamous cell carcinoma in the clinically negative neck. Int J Oral Maxillofac Surg. 2017 Jun;46(6):669-675. doi: 10.1016/j.ijom.2017.02.1277. Epub 2017 Mar 25. PMID: 28347602.

Sutton DN, Brown JS, Rogers SN, Vaughan ED, Woolgar JA. The prognostic implications of the surgical margin in oral squamous cell carcinoma. Int J Oral Maxillofac Surg. 2003 Feb;32(1):30-4. doi: 10.1054/ijom.2002.0313. PMID: 12653229.

Tadbir AA, Ashraf MJ, Sardari Y. Prognostic significance of stromal eosinophilic infiltration in oral squamous cell carcinoma. J Craniofac Surg. 2009 Mar;20(2):287-9. doi: 10.1097/SCS.0b013e318199219b. PMID: 19218858.

Tai SK, Li WY, Yang MH, Chang SY, Chu PY, Tsai TL, Wang YF, Chang PM. Treatment for T1-2 oral squamous cell carcinoma with or without perineural invasion: neck dissection and postoperative adjuvant therapy. Ann Surg Oncol. 2012 Jun;19(6):1995-2002. doi: 10.1245/s10434-011-2182-5. Epub 2011 Dec 28. PMID: 22203180.

Tarsitano A, Del Corso G, Tardio ML, Marchetti C. Tumor Infiltration Depth as Predictor of Nodal Metastasis in Early Tongue Squamous Cell Carcinoma. J Oral Maxillofac Surg. 2016 Mar;74(3):523-7. doi: 10.1016/j.joms.2015.09.015. Epub 2015 Sep 25. PMID: 26454032.

Tarsitano A, Tardio ML, Marchetti C. Impact of perineural invasion as independent prognostic factor for local and regional failure in oral squamous cell carcinoma. Oral Surg Oral Med Oral Pathol Oral Radiol. 2015 Feb;119(2):221-8. doi: 10.1016/j.oooo.2014.10.004. Epub 2014 Oct 15. PMID: 25487983.

Tazeen S, Prasad K, Harish K, Sagar P, Kapali AS, Chandramouli S. Assessment of Pretreatment Neutrophil/Lymphocyte Ratio and Platelet/Lymphocyte Ratio in Prognosis of Oral Squamous Cell Carcinoma. J Oral Maxillofac Surg. 2020 Jun;78(6):949-960. doi: 10.1016/j.joms.2020.01.001. Epub 2020 Jan 9. PMID: 32027861.

Teixeira G, Antonangelo L, Kowalski L, Saldiva P, Ferraz A, Silva Filho G. Argyrophilic nucleolar organizer regions staining is useful in predicting recurrence-free interval in oral tongue and floor of mouth squamous cell carcinoma. Am J Surg. 1996 Dec;172(6):684-8. doi: 10.1016/s0002-9610(96)00306-6. PMID: 8988678.

Tervo S, Seppälä M, Rautiainen M, Huhtala H, Salo T, Al-Samadi A, Kuopio T, Ahtiainen M, Tommola S, Paavonen T, Toppila-Salmi S. The expression and prognostic relevance of programmed cell death protein 1 in tongue squamous cell carcinoma. APMIS. 2020 Dec;128(12):626-636. doi: 10.1111/apm.13084. Epub 2020 Oct 19. PMID: 32978821.

Theocharis S, Klijanienko J, Giaginis C, Rodriguez J, Jouffroy T, Girod A, Alexandrou P, Sastre-Garau X. Histone deacetylase-1 and -2 expression in mobile tongue squamous cell carcinoma: associations with clinicopathological parameters and patients survival. J Oral Pathol Med. 2011 Oct;40(9):706-14. doi: 10.1111/j.1600-0714.2011.01031.x. Epub 2011 Apr 2. PMID: 21457345.

Thiagarajan S, Thavarool SB, Kadapa N, Nandini H. Does adverse histopathological features like perineural invasion, depth of invasion and lymphovascular invasion warrant adjuvante treatment in early oral squamous cell carcinoma?. J Head Neck Physicians Surg 2017;5:71-4.

Tinhofer I, Konschak R, Stromberger C, Raguse JD, Dreyer JH, Jöhrens K, Keilholz U, Budach V. Detection of circulating tumor cells for prediction of recurrence after adjuvant chemoradiation in locally advanced squamous cell carcinoma of the head and neck. Ann Oncol. 2014 Oct;25(10):2042-2047. doi: 10.1093/annonc/mdu271. Epub 2014 Jul 23. PMID: 25057171.

Troeltzsch M, Haidari S, Boser S, Troeltzsch M, Probst FA, Ehrenfeld M, Otto S. What Factors Are Associated With Regional Recurrence After Operative Treatment of Oral Squamous Cell Carcinoma? J Oral Maxillofac Surg. 2018 Dec;76(12):2650-2659. doi: 10.1016/j.joms.2018.07.005. Epub 2018 Jul 19. PMID: 30098956.

Tsang RK, Chung JC, To VS, Chan JY, Ho WK, Wei WI. Efficacy of salvage neck dissection for isolated nodal recurrences in early carcinoma of oral tongue with watchful waiting management of initial N0 neck. Head Neck. 2011 Oct;33(10):1482-5. doi: 10.1002/hed.21643. Epub 2010 Dec 6. PMID: 21928421.

Unal OF, Ayhan A, Hoşal AS. Prognostic value of p53 expression and histopathological parameters in squamous cell carcinoma of oral tongue. J Laryngol Otol. 1999 May;113(5):446-50. doi: 10.1017/s0022215100144184. PMID: 10505159.

Urist MM, O'Brien CJ, Soong SJ, Visscher DW, Maddox WA. Squamous cell carcinoma of the buccal mucosa: analysis of prognostic factors. Am J Surg. 1987 Oct;154(4):411-4. doi: 10.1016/0002-9610(89)90014-7. PMID: 3661845.

Varsha BK, Radhika MB, Makarla S, Kuriakose MA, Kiran GS, Padmalatha GV. Perineural invasion in oral squamous cell carcinoma: Case series and review of literature. J Oral Maxillofac Pathol. 2015 Sep-Dec;19(3):335-41. doi: 10.4103/0973-029X.174630. PMID: 26980962; PMCID: PMC4774287.

Vashist YK, Blessmann M, Trump F, Kalinin V, Kutup A, Schneider C, Gawad K, Kaifi JT, Schmelzle R, Izbicki JR, Yekebas EF. Microsatellite GTn-repeat polymorphism in the promoter of heme oxygenase-1 gene is an independent predictor of tumor recurrence in male oral squamous cell carcinoma patients. J Oral Pathol Med. 2008 Sep;37(8):480-4. doi: 10.1111/j.1600-0714.2008.00639.x. Epub 2008 Feb 28. PMID: 18312299.

Vered M, Dobriyan A, Dayan D, Yahalom R, Talmi YP, Bedrin L, Barshack I, Taicher S. Tumor-host histopathologic variables, stromal myofibroblasts and risk score, are significantly associated with recurrent disease in tongue cancer. Cancer Sci. 2010 Jan;101(1):274-80. doi: 10.1111/j.1349-7006.2009.01357.x. Epub 2009 Sep 10. PMID: 19804423.

Wang C, Huang H, Huang Z, Wang A, Chen X, Huang L, Zhou X, Liu X. Tumor budding correlates with poor prognosis and epithelial-mesenchymal transition in tongue squamous cell carcinoma. J Oral Pathol Med. 2011 Aug;40(7):545-51. doi: 10.1111/j.1600-0714.2011.01041.x. Epub 2011 Apr 12. PMID: 21481005; PMCID: PMC3135705.

Wang Y, Zhang X, Zhang Y, Zhu Y, Yuan C, Qi B, Zhang W, Wang D, Ding X, Wu H, Cheng J. Overexpression of pyruvate kinase M2 associates with aggressive clinicopathological features and unfavorable prognosis in oral squamous cell carcinoma. Cancer Biol Ther. 2015;16(6):839-45. doi: 10.1080/15384047.2015.1030551. Epub 2015 May 13. PMID: 25970228; PMCID: PMC4622565.

Weckx A, Riekert M, Grandoch A, Schick V, Zöller JE, Kreppel M. Time to recurrence and patient survival in recurrent oral squamous cell carcinoma. Oral Oncol. 2019 Jul;94:8-13. doi: 10.1016/j.oraloncology.2019.05.002. Epub 2019 May 8. PMID: 31178216.

Weimar EAM, Huang SH, Lu L, O'Sullivan B, Perez-Ordonez B, Weinreb I, Hope A, Tong L, Goldstein D, Irish J, de Almeida JR, Bratman S, Xu W, Yu E. Radiologic-Pathologic Correlation of Tumor Thickness and Its Prognostic Importance in Squamous Cell Carcinoma of the Oral Cavity: Implications for the Eighth Edition Tumor, Node, Metastasis Classification. AJNR Am J Neuroradiol. 2018 Oct;39(10):1896-1902. doi: 10.3174/ajnr.A5782. Epub 2018 Aug 30. PMID: 30166432; PMCID: PMC7410722.

Wenzel S, Sagowski C, Kehrl W, Metternich FU. The prognostic impact of metastatic pattern of lymph nodes in patients with oral and oropharyngeal squamous cell carcinomas. Eur Arch Otorhinolaryngol. 2004 May;261(5):270-5. doi: 10.1007/s00405-003-0678-8. Epub 2003 Sep 18. PMID: 14504863.

Williams JK, Carlson GW, Cohen C, Derose PB, Hunter S, Jurkiewicz MJ. Tumor angiogenesis as a prognostic factor in oral cavity tumors. Am J Surg. 1994 Nov;168(5):373-80. doi: 10.1016/s0002-9610(05)80079-0. PMID: 7526718.

Woolgar JA. T2 carcinoma of the tongue: the histopathologist's perspective. Br J Oral Maxillofac Surg. 1999 Jun;37(3):187-93. doi: 10.1054/bjom.1999.0034. PMID: 10454025.

Woolgar JA, Rogers SN, Lowe D, Brown JS, Vaughan ED. Cervical lymph node metastasis in oral cancer: the importance of even microscopic extracapsular spread. Oral Oncol. 2003 Feb;39(2):130-7. doi: 10.1016/s1368-8375(02)00030-1. PMID: 12509965.

Woolgar JA, Rogers S, West CR, Errington RD, Brown JS, Vaughan ED. Survival and patterns of recurrence in 200 oral cancer patients treated by radical surgery and neck dissection. Oral Oncol. 1999 May;35(3):257-65. doi: 10.1016/s1368-8375(98)00113-4. PMID: 10621845.

Woolgar JA, Scott J. Prediction of cervical lymph node metastasis in squamous cell carcinoma of the tongue/floor of mouth. Head Neck. 1995 Nov-Dec;17(6):463-72. doi: 10.1002/hed.2880170603. PMID: 8847204.

Woolgar JA, Scott J, Vaughan ED, Brown JS, West CR, Rogers S. Survival, metastasis and recurrence of oral cancer in relation to pathological features. Ann R Coll Surg Engl. 1995 Sep;77(5):325-31. PMID: 7486755; PMCID: PMC2502417.

Wu K, Yang X, Li L, Ruan M, Liu W, Lu W, Zhang C, Li S. Neurovascular Invasion and Histological Grade Serve as the Risk Factors of Cervical Lymph Node Metastases in Early Tongue Squamous Cell Carcinoma. Mol Neurobiol. 2016 Jul;53(5):2920-2926. doi: 10.1007/s12035-015-9175-5. Epub 2015 Apr 25. PMID: 25911199.

Wunschel M, Neumeier M, Utpatel K, Reichert TE, Ettl T, Spanier G. Staging more important than grading? Evaluation of malignancy grading, depth of invasion, and resection margins in oral squamous cell carcinoma. Clin Oral Investig. 2021 Mar;25(3):1169-1182. doi: 10.1007/s00784-020-03421-2. Epub 2020 Jun 29. PMID: 32601998; PMCID: PMC7878266.

Xie N, Wang C, Liu X, Li R, Hou J, Chen X, Huang H. Tumor budding correlates with occult cervical lymph node metastasis and poor prognosis in clinical early-stage tongue squamous cell carcinoma. J Oral Pathol Med. 2015 Apr;44(4):266-72. doi: 10.1111/jop.12242. Epub 2014 Aug 28. PMID: 25169851.

Xie N, Wang C, Zhuang Z, Hou J, Liu X, Wu Y, Liu H, Huang H. Decreased miR-320a promotes invasion and metastasis of tumor budding cells in tongue squamous cell carcinoma. Oncotarget. 2016 Oct 4;7(40):65744-65757. doi: 10.18632/oncotarget.11612. PMID: 27582550; PMCID: PMC5323189.

Xu Q, Wang C, Yuan X, Feng Z, Han Z. Prognostic Value of Tumor-Infiltrating Lymphocytes for Patients With Head and Neck Squamous Cell Carcinoma. Transl Oncol. 2017 Feb;10(1):10-16. doi: 10.1016/j.tranon.2016.10.005. Epub 2016 Nov 23. PMID: 27888708; PMCID: PMC5123038.

Yamada SI, Otsuru M, Yanamoto S, Hasegawa T, Aizawa H, Kamata T, Yamakawa N, Kohgo T, Ito A, Noda Y, Hirai C, Kitamura T, Okura M, Kirita T, Ueda M, Yamashita T, Ota Y, Komori T, Umeda M, Kurita H. Progression level of extracapsular spread and tumor budding for cervical lymph node metastasis of OSCC. Clin Oral Investig. 2018 Apr;22(3):1311-1318. doi: 10.1007/s00784-017-2231-y. Epub 2017 Oct 6. PMID: 28986696.

Yamamoto N, Osaka R, Watabe Y, Takano N, Matsuzaka K, Shibahara T. Clinical study of mode of invasion in tongue squamous cell carcinoma. Journal of Oral and Maxillofacial Surgery, Medicine, and Pathology. 2014 Jul; 26(3):287-291. Doi: 10.1016/j.ajoms.2013.02.012.

Yanamoto S, Yamada S, Takahashi H, Yoshitomi I, Kawasaki G, Ikeda H, Minamizato T, Shiraishi T, Fujita S, Ikeda T, Asahina I, Umeda M. Clinicopathological risk factors for local recurrence in oral squamous cell carcinoma. Int J Oral Maxillofac Surg. 2012 Oct;41(10):1195-200. doi: 10.1016/j.ijom.2012.07.011. Epub 2012 Aug 14. PMID: 22901502.

Yang SW, Lee YS, Chang LC, Hsieh TY, Chen TA. Implications of morphologic patterns of intraepithelial microvasculature observed by narrow-band imaging system in cases of oral squamous cell carcinoma. Oral Oncol. 2013 Jan;49(1):86-92. doi: 10.1016/j.oraloncology.2012.07.001. Epub 2012 Jul 25. PMID: 22840786.

Yang TL, Lou PJ, Chang YL, Wu CT, Wang CP, Ko JY. Tumor satellite in predicting occult nodal metastasis of tongue cancer. Otolaryngol Head Neck Surg. 2011 Oct;145(4):599-605. doi: 10.1177/0194599811411635. Epub 2011 Jun 13. PMID: 21670477.

Yang TL, Wang CP, Ko JY, Lin CF, Lou PJ. Association of tumor satellite distance with prognosis and contralateral neck recurrence of tongue squamous cell carcinoma. Head Neck. 2008 May;30(5):631-8. doi: 10.1002/hed.20758. PMID: 18213729.

Yeh CF, Li WY, Yang MH, Chu PY, Lu YT, Wang YF, Chang PM, Tai SK. Neck observation is appropriate in T1-2, cN0 oral squamous cell carcinoma without perineural invasion or lymphovascular invasion. Oral Oncol. 2014 Sep;50(9):857-62. doi: 10.1016/j.oraloncology.2014.06.002. Epub 2014 Jul 4. PMID: 24998199.

Yellapurkar S, Natarajan S, Boaz K, Manaktala N, Baliga M, Shetty P, Prasad M, Ravi M. Expression of Laminin in Oral Squamous Cell Carcinomas. Asian Pac J Cancer Prev. 2018 Feb 26;19(2):407-413. doi: 10.22034/APJCP.2018.19.2.407. PMID: 29479990; PMCID: PMC5980927.

Yonezawa N, Minamikawa T, Kitajima K, Takahashi Y, Sasaki R, Nibu KI, Komori T. The maximum standardized uptake value increment calculated by dual-time-point ^18^F-fluorodeoxyglucose positron emission tomography predicts survival in patients with oral tongue squamous cell carcinoma. Nagoya J Med Sci. 2017 Feb;79(2):189-198. doi: 10.18999/nagjms.79.2.189. PMID: 28626254; PMCID: PMC5472544.

Yoshizawa K, Nozaki S, Kitahara H, Kato K, Noguchi N, Kawashiri S, Yamamoto E. Expression of urokinase-type plasminogen activator/urokinase-type plasminogen activator receptor and maspin in oral squamous cell carcinoma: Association with mode of invasion and clinicopathological factors. Oncol Rep. 2011 Dec;26(6):1555-60. doi: 10.3892/or.2011.1419. Epub 2011 Aug 10. PMID: 21833477.

Yu EH, Lui MT, Tu HF, Wu CH, Lo WL, Yang CC, Chang KW, Kao SY. Oral carcinoma with perineural invasion has higher nerve growth factor expression and worse prognosis. Oral Dis. 2014 Apr;20(3):268-74. doi: 10.1111/odi.12101. Epub 2013 Apr 5. PMID: 23556997.

Yu SY, Wang YP, Chang JY, Shen WR, Chen HM, Chiang CP. Increased expression of MCM5 is significantly associated with aggressive progression and poor prognosis of oral squamous cell carcinoma. J Oral Pathol Med. 2014 May;43(5):344-9. doi: 10.1111/jop.12134. Epub 2013 Nov 18. PMID: 24245508.

Yuasa-Nakagawa K, Shibuya H, Yoshimura R, Miura M, Watanabe H, Kishimoto S, Omura K. Cervical lymph node metastasis from early-stage squamous cell carcinoma of the oral tongue. Acta Otolaryngol. 2013 May;133(5):544-51. doi: 10.3109/00016489.2012.748988. Epub 2013 Jan 28. PMID: 23350600.

Yue LE, Sharif KF, Sims JR, Sandler ML, Baik FM, Sobotka S, Everest S, Brandwein-Weber M, Khorsandi AS, Likhterov I, Urken ML. Oral squamous carcinoma: Aggressive tumor pattern of invasion predicts direct mandible invasion. Head Neck. 2020 Nov;42(11):3171-3178. doi: 10.1002/hed.26360. Epub 2020 Jul 25. PMID: 32710523.

Yuen AP, Lam KY, Wei WI, Lam KY, Ho CM, Chow TL, Yuen WF. A comparison of the prognostic significance of tumor diameter, length, width, thickness, area, volume, and clinicopathological features of oral tongue carcinoma. Am J Surg. 2000 Aug;180(2):139-43. doi: 10.1016/s0002-9610(00)00433-5. PMID: 11044531.

Yuen APW, Lam KY, Lam LK, Ho CM, Wong A, Chow TL, Yuen WF, Wei WI. Prognostic factors of clinically stage I and II oral tongue carcinoma-A comparative study of stage, thickness, shape, growth pattern, invasive front malignancy grading, Martinez-Gimeno score, and pathologic features. Head Neck. 2002 Jun;24(6):513-20. doi: 10.1002/hed.10094. PMID: 12112547.

Zhang P, Zhang L, Liu H, Zhao L, Li Y, Shen JX, Liu Q, Liu MZ, Xi M. Clinicopathologic Characteristics and Prognosis of Tongue Squamous Cell Carcinoma in Patients with and without a History of Radiation for Nasopharyngeal Carcinoma: A Matched Case-Control Study. Cancer Res Treat. 2017 Jul;49(3):695-705. doi: 10.4143/crt.2016.317. Epub 2016 Oct 11. PMID: 27737535; PMCID: PMC5512375.

Zhao TC, Liang SY, Ju WT, Fu Y, Zhou ZH, Wang LZ, Li J, Zhang CP, Zhang ZY, Zhong LP. High-risk lymph node ratio predicts worse prognosis in patients with locally advanced oral cancer. J Oral Pathol Med. 2020 Sep;49(8):787-795. doi: 10.1111/jop.13043. Epub 2020 Jun 7. PMID: 32449223; PMCID: PMC7540480.
