# Supplementary material for: The Impact of Histopathological Features on the Prognosis of Oral Squamous Cell Carcinoma: A Comprehensive Review and Meta-Analysis
Source: Front Oncol. 2021 Nov 10;11:784924. doi: 10.3389/fonc.2021.784924 (PMC8631280; doi:10.3389/fonc.2021.784924)
Supplement: Supplementary file 1 [file DataSheet_1.zip › Supplementary Table 2.DOCX]

SupplementaryTable 2. Overview of the 172 studies included in the meta-analysis.

| Year | Study | Country | Number of samples | Site | Clinical Stage | Histological Parameters | Outcome | |
| --- | --- | --- | --- | --- | --- | --- | --- | --- |
| 2021 | Moreira et al. | France | 151 | Oral cavity | All stage | ENE, Margin (clear vs. involved) | OS, DFS | |
| 2021 | Sambasivan et al. | UK | 172 | Oral cavity | Advanced stage | PNI, LVI, Margin (cut-off 5 mm) | OS |  |
| 2021 | Nguyen et al. | New Zealand | 70 | Oral cavity | Early stage | PNI | OS, DFS |  |
| 2021 | Xu et al. | USA | 329 | Oal tongue | All stage | Tumor budding (0-4, 5-9, ≥10 buds), WPOI (1/2/3/4 vs. 5) | OS |  |
| 2021 | Kikuchi et al. | Japan | 103 | Oral cavity | All stage | Margin (clear vs. involved), ENE | OS |  |
| 2021 | Lau et al. | Singapure | 88 | Oral tongue | All stage | Margin (clear vs. close/margin), DOI (cut-off 6 mm) | OS, DSS, DFS |  |
| 2021 | Mneimneh et al. | USA | 147 | Oral cavity | All stage | PNI, DOI (<5, 5,1-10, >10 mm), Margin (clear vs. involved), Tumor thickness (<5, 5,1-10, >10 mm), Pattern of invasion (WPOI 2/3/4/5), LVI | OS, DSS |  |
| 2020 | Tsuchihashi et al. | Japan | 226 | Oral cavity | All stage | TSR (cut-off 50%), Pattern of invasion (Yamamoto’s system) | OS, DFS |  |
| 2020 | Chang, W-C et al. | Taiwan | 320 | Oral cavity, including lip | All stage | ENE | OS |  |
| 2020 | Mascitti et al. | Italy | 66 | Oral cavity | All stage | PNI, LVI | DSS |  |
| 2020 | Dourado et al. | Brazil | 254 | Oral cavity | All stage | TSR (cut-off 50%), Tumor budding (cut-off 5 buds) | DSS, DFS |  |
| 2020 | Hoffmann et al. | France | 124 | Oral cavity | All stage | PNI | OS, DSS |  |
| 2020 | Lin et al. | Taiwan | 2535 | Oral cavity | All stage | ENE | DFS |  |
| 2020 | Bajwa et al. | England | 631 | Oral cavity, including lip | All stage | Margin (cut-off >5, 1-4.9, 0.1-0.9, 0 mm) | OS, DSS, DFS |  |
| 2020 | Mascitti et al. | Italy | 139 | Oral tongue | All stage | PNI, TSR (cut-off 50%) | DSS, DFS |  |
| 2020 | Lien et al. | Taiwan | 385 | Oral cavity, including lip | All stage | ENE, PNI, LVI, Margin (cut-off 5 mm), DOI (cut-off 5 mm) | OS, DSS |  |
| 2020 | Rodrigues et al. | Brazil | 380 | Oral tongue and floor of mouth | All stage | PNI | OS, DSS, DFS |  |
| 2020 | Domingueti et al. | Brazil | 243 | Oral cavity | All stage | Tumor budding (cut-off 5 buds), DOI (cut-off 4 mm), Inflammatory response (KM grade) | DSS |  |
| 2020 | Hori et al. | Japan | 62 | Oral tongue | Early stage | Lymphatic invasion, Vascular invasion, Pattern of invasion (WPOI 1/2/3 vs. 4/5) | DFS, NRFS |  |
| 2020 | Cheng et al. | Taiwan | 191 | Oral cavity | Advanced stage | PNI, ENE, Margin (clear vs. involved) | OS, DFS |  |
| 2020 | Stoop et al. | Netherlands | 210 | Tumors with mandibular invasion | NA | LVI, PNI, Margin (cut-off 1 mm) | DSS, DFS |  |
| 2020 | Tay et al. | Taiwan | 358 | Oral cavity, with lip | All stage | PNI, ENE, LVI, DOI (cut-off 5 mm), Margin (cut-off 5 mm) | OS, DSS |  |
| 2020 | Marinelli et al. | USA | 52 | Buccal mucosa | All stage | Pattern of invasion (WPOI 1/2/3/4 vs. 5), PNI | OS |  |
| 2020 | Parekh et al. | India | 95 | Oral cavity, with lip | Early stage | Inflammatory response (B-G grade) | DFS |  |
| 2020 | Troiano et al. | Italy | 221 | Oral tongue | All stage | Inflammatory response (TILs cut-off 10%), PNI | DSS, DFS |  |
| 2020 | Kurihara-Shimomura et al. | Japan | 111 | Oral cavity | All stage | Pattern of invasion (WPOI 1/2/3 vs. 4/5) | DFS |  |
| 2020 | Spoerl et al. | Germany | 290 | Oral cavity | All stage | ENE | OS |  |
| 2019 | Safi et al. | Germany | 334 | Oral cavity | All stage | Margin (clean vs. involved) | OS |  |
| 2019 | Chang, W-C et al. | Taiwan | 341 | Oral cavity, including lip | All stage | DOI (cut-off 5 mm) | OS |  |
| 2019 | Rajappa et al. | India | 792 | Oral cavity | Early stage | DOI (cut-off 5 mm), LVI, PNI, Margin (cut-off 5 mm) | OS, LRFS, DRFS |  |
| 2019 | Yamakawa et al. | Japan | 337 | Oral tongue | Early stage | Tumor budding (cut-off 5 buds), DOI (cut-off 4 mm), LVI | NRFS |  |
| 2019 | Almeida et al. | Canada | 551 | Oral cavity | All stage | ENE, PNI | OS, DFS |  |
| 2019 | Wu et al. | China | 141 | Oral tongue | Early stage | DOI (cut-off 4 mm), Neurovascular invasion | OS, DFS |  |
| 2019 | Heikkinen et al. | Finland and Brazil | 308 | Oral tongue | Early stage | Inflammatory response (TILs cut-off 20%) | OS, DSS, DFS |  |
| 2019 | Subramaniam et al. | India | 296 | Oral cavity | Early stage | LVI, PNI, DOI (cut-off 5 mm) | LRRFS |  |
| 2019 | Al Feghali et al. | USA | 163 | Oral cavity | All stage | LVI, PNI | OS, DFS |  |
| 2019 | Ho et al. | Taiwan | 200 | Oral cavity, including lip | All stage | ENE, LVI, Tumor budding (absence vs. presence), DOI (cut-off 5.5 mm), PNI | DFS |  |
| 2019 | De Paz et al. | Taiwan | 246 | Oral cavity, including lip | All stage | ENE, DOI (cut-off 10 mm) | OS, DFS |  |
| 2019 | Xie et al. | China | 136 | Oral tongue | All stage | Tumor budding (cut-off 5 buds) | OS |  |
| 2019 | Kozak et al. | USA | 126 | Oral cavity, including lip | Early stage | DOI (cut-off 4 mm) | OS, DFS |  |
| 2019 | Wei et al. | Taiwan | 314 | Oral tongue and buccal mucosa | All stage | PNI, Margin (clean vs. involved), LVI, Tumor thickness (cut-off 4 mm) | OS, DSS |  |
| 2019 | Agarwal. et al. | India | 94 | Oral cavity, including lip | All stage | ENE (grades), PNI | OS, DFS |  |
| 2019 | Roh et al. | South Korea | 322 | Oral cavity, including lip | All stage | DOI (cut-off <5, 5.1-10, >10 mm), ENE | OS, DFS |  |
| 2019 | Yu et al. | China | 246 | Oral tongue | All stage | Tumor budding (cut-off 5 buds), Inflammatory response (KM grade) | OS, DFS |  |
| 2019 | De Paz et al. | Taiwan | 259 | Oral tongue | All stage | ENE, PNI, DOI (cut-off 9.5 mm) | OS, DFS |  |
| 2019 | Sridharan et al. | USA | 494 | Oral tongue | Early stage | PNI | LRFS |  |
| 2019 | Hasmat et al. | Australia | 861 | Oral cavity | All stage | Margin (clean vs. involved) | DSS |  |
| 2019 | Ebihara et al. | Japan | 64 | Oral tongue | Early stage | Vascular invasion, DOI (cut-off 5 mm), Tumor budding (cut-off <4, 5-9, ≥10 buds) | DSS |  |
| 2019 | Lu et al. | China | 73 | Oral cavity | All stage | DOI (cut-off 5 mm) | OS |  |
| 2019 | Zhang et al. | China | 80 | Oral cavity | All stage | Tumor budding (cut-off 5 buds) | OS |  |
| 2019 | Slieker et al. | Netherlands | 95 | Maxilla | All stage | Margin (clean/close vs. involved), Vasoinvasive invasion | OS |  |
| 2019 | Lee, L-Y et al. | Taiwan | 229 | Oral cavity | All stage | PNI, ENE, Lymphatic invasion | OS, DFS |  |
| 2019 | Ding et al. | USA | 149 | Oral cavity | All stage | ENE, Margin (cut-off 5 mm) | OS, DFS, LRRFS, DRFS |  |
| 2019 | Rubin et al. | USA | 934 | Oral tongue | Early stage | DOI (cut-off 5 mm) | OS |  |
| 2019 | Oliver et al. | USA | 22930 | Oral tongue | All stage | LVI, ENE, Margin (clean vs. involved) | OS |  |
| 2019 | Sharma et al. | India | 202 | Oral tongue | All stage | ENE, LVI, PNI | OS, LRRFS |  |
| 2018 | Nair et al. | India | 1524 | Oral tongue and buccal mucosa | All stage | PNI | OS, DFS |  |
| 2018 | Subramaniam et al. | India | 442 | Oral cavity | All stage | PNI, LVI, ENE | OS |  |
| 2018 | Safi et al. | Germany | 89 | Tumors with mandibular invasion | Advanced stage | PNI | LRRFS |  |
| 2018 | Cracchiolo et al. | USA | 381 | Oral tongue | All stage | PNI | DSS, LRFS, NRFS |  |
| 2018 | Mizrachi et al. | USA | 1302 | Oral cavity | All stage | LVI, PNI, Tumor thickness (cut-off 4 mm) | DFS |  |
| 2018 | Szewczyk et al. | Poland | 151 | Oral cavity | All stage | LVI, PNI | LRFS, NRFS |  |
| 2018 | Wolfer et al. | Germany | 151 | Oral cavity | All stage | Margin (cut-off 5 mm), LVI, ENE | DSS, DFS |  |
| 2018 | Lee et al. | South Korea | 231 | Oral cavity | All stage | DOI (cut-off <5, 5.1-10, >10 mm), LVI | OS, DSS, DFS |  |
| 2018 | Shukla et al. | India | 124 | Oral tongue | All stage | Margin (cut-off 5 mm) | DFS |  |
| 2018 | Xu et al. | China | 1750 | Oral cavity | All stage | PNI, ENE | DFS |  |
| 2018 | Almangush et al. | Finland and Brazil | 331 | Oral tongue | Early stage | TSR (cut-off 50%) | DSS, DFS |  |
| 2018 | Yang et al. | China | 221 | Oral tongue | Early stage | PNI | DSS, DFS |  |
| 2018 | Cheng et al. | Taiwan | 8986 | Oral cavity, including lip | All stage | Margin (clean vs. involved) | OS, DSS, LRRFS, DRFS |  |
| 2018 | Sakata et al. | Japan | 97 | Oral tongue | Early stage | Pattern of invasion (WPOI 1/2/3 vs. 4/5), PNI, DOI (cut-off 3.3 mm), Tumor budding (cut-off 4 buds) | DSS |  |
| 2018 | Yoshida et al. | Japan | 78 | Gingiva | All stage | LVI, PNI, ENE, Bone invasion | OS, DSS |  |
| 2018 | Jing et al. | China | 109 | Oral cavity | All stage | Pattern of invasion (WPOI 1/2/3 vs. 4/5) | OS, DFS |  |
| 2018 | Pu et al. | China | 311 | Oral cavity | All stage | PNI, Pattern of invasion (WPOI 1/2/3 vs. 4/5), DOI (cut-off 5 mm) | OS, DFS |  |
| 2018 | Buchakjian et al. | USA | 426 | Oral cavity | All stage | Margin (clean vs. involved), ENE | OS, LRFS |  |
| 2018 | Tamatani et al. | Japan | 70 | Oral cavity | Early stage | Pattern of invasion (Yamamoto’s system) | DFS |  |
| 2018 | Miyazaki et al. | Japan | 60 | Oral tongue | Early stage | Pattern of invasion (Yamamoto’s system) | DFS |  |
| 2017 | Safi et al. | Germany | 517 | Oral cavity | All stage | ENE | DFS |  |
| 2017 | Niu et al. | China | 207 | Mandibular gingiva | All stage | ENE, PNI | OS |  |
| 2017 | Ho et al. | USA | 14554 | Oral cavity | All stage | ENE, Margin (clean vs. involved) | OS |  |
| 2017 | Liu et al. | Taiwan | 1383 | Oral cavity | All stage | PNI, LVI, ENE, Margin (cut-off 5 mm) | DSS |  |
| 2017 | Lai et al. | Taiwan | 150 | Oral cavity | All stage | Margin (clean vs. involved/close), ENE, LVI, PNI | LRRFS |  |
| 2017 | Safi et al. | Germany | 130 | Oral tongue | All stage | PNI | LRRFS |  |
| 2017 | Yu et al. | Taiwan | 100 | Oral cavity | All stage | PNI, LVI | DFS |  |
| 2017 | Cassidy et al. | USA | 180 | Oral tongue | All stage | LVI, DOI (cut-off 3 mm), PNI | OS, LRRFS |  |
| 2017 | Takahashi et al. | Japan | 73 | Oral tongue | All stage | Lymphatic invasion, Vascular invasion | OS, DFS |  |
| 2017 | Petrovic et al. | USA | 326 | Oral cavity | All stage | Bone invasion, PNI | DSS, LRFS |  |
| 2017 | Hasegawa et al. | Japan | 291 | Oral cavity | All stage | ENE | OS |  |
| 2017 | Nagam et al. | India | 291 | Oral tongue and buccal mucosa | All stage | LVI, PNI | OS |  |
| 2017 | Lee et al. | Taiwan | 396 | Oral cavity | All stage | PNI | DSS |  |
| 2017 | Zanoni et al. | USA | 381 | Oral tongue | All stage | PNI, Margin (cut-off 2.2 mm) | DFS |  |
| 2017 | Padma et al. | India | 198 | Buccal mucosa | All stage | LVI, PNI, DOI (cut-off <2, 2-4, 4-6, >6 mm) | DFS |  |
| 2017 | Huang et al. | Taiwan | 194 | Oral cavity | All stage | DOI (cut-off 10 mm), ENE | OS, DFS |  |
| 2017 | Hori et al. | Japan | 48 | Oral tongue | Early stage | Tumor budding (cut-off 5 buds) | NRFS |  |
| 2017 | Hosni et al. | Canada | 914 | Oral cavity | All stage | ENE, PNI | OS, NRFS, DRFS |  |
| 2017 | Wang and Veivers | Australia | 112 | Oral tongue | All stage | Tumor thickness (cut-off <2, 2-3.9, ≥4 mm), Margin (clear vs. close/involved), ENE | OS, DSS |  |
| 2017 | Satgunaseelan et al. | Australia | 215 | Oral cavity | All stage | DOI (cut-off <5, 5-10, >10 mm), ENE | OS, DFS |  |
| 2017 | Fan, K-H et al. | Taiwan | 109 | Oral cavity | All stage | DOI (cut-off 11 mm) | OS, DFS |  |
| 2017 | Nakao et al. | Japan | 58 | Oral tongue | All stage | Pattern of invasion (Yamamoto’s system) | DSS |  |
| 2017 | Jang et al. | South Korea | 325 | Oral cavity | All stage | PNI, Margin (cut-off 5 mm) | LRFS |  |
| 2017 | Quinlan-Davidson et al. | USA | 289 | Oral cavity | All stage | ENE, LVI, Margin (cut-off 5 mm) | OS |  |
| 2016 | Fives et al. | Ireland | 54 | Floor of mouth | All stage | Pattern of invasion (cohesive vs. noncohesive), LVI | OS |  |
| 2016 | Heerema et al. | Netherlands | 211 | Oral cavity | All stage | Pattern of invasion (cohesive vs. noncohesive), PNI, Margin (clean vs. involved) | DSS, LRRFS |  |
| 2016 | Wang et al. | Taiwan | 345 | Oral cavity, including lip | All stage | ENE | OS, DSS, LRRDS, DRFS |  |
| 2016 | Kim et al. | South Korea | 316 | Oral cavity | All stage | Margin (clean vs. involved) | OS |  |
| 2016 | Liu et al. | Canada | 322 | Oral cavity | All stage | DOI (cut-off 4 mm) | NRFS |  |
| 2016 | Lapke et al. | Taiwan | 333 | Oral cavity, including lip | All stage | ENE | DSS |  |
| 2016 | Chen et al. | Taiwan | 567 | Oral cavity | All stage | ENE, lymphatic invasion, Margin (cut-off 1 mm) | OS, LRRFS, DRFS |  |
| 2016 | Xie et al. | China | 641 | Oral cavity | All stage | Margin (clear vs. involved) | OS |  |
| 2016 | Mücke et al. | Germany | 492 | Oral tongue | All stage | Tumor thickness (cut-off 8 mm) | OS |  |
| 2016 | Heiduschka et al. | Australia | 501 | Oral cavity | All stage | PNI, LVI, ENE | DSS, DFS |  |
| 2016 | Adel et al. | Taiwan | 277 | Oral cavity | All stage | DOI (cut-off 10 mm), ENE | OS, DFS |  |
| 2016 | Shinagawa et al. | Japan | 116 | Oral cavity | All stage | Pattern of invasion (Bryne’s classification), Vascular invasion, PNI | DSS, DFS |  |
| 2015 | Almangush et al. | Finland and Brazil | 311 | Oral tongue | Early stage | DOI (cut-off 4 mm), Tumor budding (cut-off 5 buds), Pattern of invasion (WPOI, low vs. high) | DSS, DFS |  |
| 2015 | Jardim et al. | Brazil | 142 | Oral tongue and floor of mouth | Advanced stage | PNI, ENE | OS, DFS |  |
| 2015 | Chen et al. | Taiwan | 218 | Oral cavity | Advanced stage | Margin (clear vs. involved), PNI, LVI | OS, DFS |  |
| 2015 | Dillon et al. | USA | 54 | Oral cavity | All stage | Margin (cut-off >5, 1-5, <1 mm), ENE, PNI, LVI | OS, DFS |  |
| 2015 | Luryi et al. | USA | 6830 | Oral cavity, with lip | Early stage | Margin (cut-off 5 mm) | OS |  |
| 2015 | Lin et al. | Taiwan | 554 | Oral cavity | All stage | PNI, LVI, ENE | OS, DSS, DFS |  |
| 2015 | Manjula et al. | India | 33 | Buccal mucosa and gingiva | All stage | Tumor thickness (cut-off 5 mm) | DFS |  |
| 2015 | D'Cruz et al. | India | 496 | Oral cavity | Early stage | Margin (cut-off 5 mm), DOI (continuous variable) | OS, DFS |  |
| 2015 | Xie et al. | China | 195 | Oral tongue | Early stage | DOI (cut-off 4 mm), Tumor budding (cut-off 5 buds) | OS |  |
| 2015 | Aivazian et al. | Australia | 318 | Oral cavity | All stage | PNI | DSS, LRFS |  |
| 2015 | Kong et al. | Malaysia | 87 | Oral cavity, including lip | All stage | Pattern of invasion (cohesive vs. noncohesive) | OS |  |
| 2014 | Thiagarajan et al. | India | 586 | Oral tongue | All stage | Tumor thickness (cut-off 11 mm), ENE, PNI | OS, DFS |  |
| 2014 | Pinto et al. | Brazil | 57 | Oral cavity | All stage | LVI, PNI, Tumor thickness (cut-off 10 mm) | DFS |  |
| 2014 | Chatzistefanou et al. | USA | 367 | Oral cavity | All stage | PNI | LRFS, NRFS |  |
| 2014 | Monteiro et al. | Portugal | 128 | Oral cavity | All stage | PNI | OS |  |
| 2014 | Fan, K-H et al. | Taiwan | 86 | Oral cavity | Advanced stage | Lymphatic invasion | OS |  |
| 2014 | Chen et al. | Taiwan | 618 | Oral cavity | All stage | PNI, LVI, Tumor emboli | OS |  |
| 2014 | Hedbäck et al. | Denmark | 86 | Oral tongue and floor of mouth | All stage | PNI | DFS |  |
| 2014 | Feng et al. | China | 844 | Oral cavity | All stage | ENE, Pattern of invasion (infiltrative vs. non-infiltrative) | DSS, NRFS |  |
| 2014 | Ebrahimi et al. | Italy, Brazil, Israel, Australia, Germany, USA, Taiwan, India | 3149 | Oral cavity | All stage | ENE, Margin (cut-off 5 mm), DOI (continuous variable) | DSS |  |
| 2014 | Su et al. | Taiwan | 135 | Oral cavity | All stage | Margin (clean vs. involved) | OS |  |
| 2014 | Yanase et al. | Japan | 61 | Oral cavity | All stage | Pattern of invasion (Yamamoto’s system) | OS |  |
| 2013 | Ganly et al. | USA/Canada | 164 | Oral tongue | All stage | Tumor thickness (cut-off 4 mm) | NRFS |  |
| 2013 | Li et al. | USA/Canada/Israel | 299 | Oral cavity | Early stage | Pattern of invasion (WPOI, 1/2/3 vs. 4/5 or WPOI, 1/2/3/4 vs. 5) | DSS, DFS |  |
| 2013 | Ling et al. | China | 210 | Oral tongue | All stage | DOI (cut-off <4, 4-9, >9 mm), PNI, Margin (cut-off 5 mm) | OS, DSS, LRRFS |  |
| 2013 | Perisanidis et al. | Austria | 97 | Oral cavity | Advanced stage | PNI | DSS |  |
| 2013 | Durr et al. | USA | 120 | Oral tongue | All stage | Pattern of invasion (cohesive vs. noncohesive), LVI, PNI, | OS, DFS |  |
| 2013 | Tai et al. | Taiwan | 212 | Oral tongue and buccal mucosa | Early stage | Tumor thickness (cut-off 6 mm), PNI | DSS |  |
| 2013 | Amit et al. | Italy, Austria, Brazil, Germany, India, USA, Taiwan | 2738 | Oral cavity | All stage | Margin (clear vs. involved), DOI (cut-off 4 mm) | OS, DSS |  |
| 2013 | Chinn et al. | USA | 88 | Oral cavity | All stage | PNI, Vascular invasion | OS, DSS, DFS, LRRFS |  |
| 2013 | Montero et al. | USA | 1617 | Oral cavity | All stage | Bone invasion | DSS, LRRFS |  |
| 2013 | Fried et al. | USA | 254 | Oral cavity | All stage | Margin (clear and close vs. involved), Bone invasion (no invasion vs. medullary) | OS, DSS, LRFS |  |
| 2013 | Yanamoto et al. | Japan | 58 | Oral tongue | Early stage | Pattern of invasion (Bryne’s classification), DOI (cut-off 4 mm), Margin (cut-off 4 mm) | DSS, DFS, LRFS, NRFS, LRRFS |  |
| 2013 | Chen, T-C et al. | Taiwan | 442 | Oral cavity | Early stage | PNI, LVI | OS, DFS |  |
| 2012 | Ganly et al. | USA | 216 | Oral tongue | All stage | DOI (cut-off 2 mm), Margin (cut-off 1 mm) | LRFS, NRFS |  |
| 2012 | Bachar et al. | Canada | 70 | Buccal mucosa | All stage | ENE | OS, DFS |  |
| 2012 | Grimm et al. | Germany | 484 | Oral cavity, with lip | All stage | Vascular invasion, Lymphatic invasion | OS |  |
| 2012 | Petera et al. | Czech Republic | 24 | Oral tongue | All stage | DOI (cut-off 10 mm) | DFS |  |
| 2012 | Tan et al. | Singapure | 67 | Oral tongue | All stage | DOI (cut-off 4 mm) | OS, LRFS |  |
| 2012 | Zhao et al. | China | 98 | Oral cavity | All stage | Pattern of invasion (Bryne’s classification) | OS, DFS |  |
| 2011 | Mücke et al. | Germany | 773 | Oral cavity | All stage | Bone invasion | OS |  |
| 2011 | Jan et al. | Taiwan | 394 | Buccal mucosa | All stage | Margin (cut-off 5 mm), ENE | OS |  |
| 2011 | Lin et al. | Taiwan | 79 | Oral cavity | All stage | ENE | OS, DSS, DFS |  |
| 2011 | Marsh et al. | England | 282 | Oral cavity, with lip | All stage | Margin (cut-off >5, 1-5, <1 mm), ENE, Pattern of invasion (cohesive vs. noncohesive), DOI (cut-off 5 mm) | OS |  |
| 2011 | Michikawa et al. | Japan | 127 | Oral cavity | All stage | ENE | OS, DFS |  |
| 2011 | Chen et al. | Taiwan | 59 | Oral cavity | All stage | ENE | OS, DFS |  |
| 2011 | Camisasca et al. | Brazil | 25 | Oral cavity | All stage | Pattern of invasion (WPOI, 1/2/3 vs. 4/5), Inflammatory response (B-G grade) | OS, DSS |  |
| 2011 | Ebrahimi et al. | Australia | 498 | Oral cavity | All stage | Margin (clean, close < 5 mm, involved), ENE, Bone invasion (no invasion, cortical, medullar) | OS, DSS |  |
| 2010 | Walvekar et al. | India | 207 | Gingivobuccal complex | Early stage | Bone invasion | DFS |  |
| 2010 | Shim et al. | South Korea | 86 | Oral tongue | All stage | DOI (cut-off 5mm) | OS, DFS |  |
| 2010 | Chang, K-P et al. | Taiwan | 92 | Oral cavity | All stage | PNI | OS |  |
| 2010 | Kurita et al. | Japan | 148 | Oral cavity | All stage | Pattern of invasion (Yamamoto’s system), Margin (cut-off 5 mm) | LRFS |  |
| 2010 | Fan, K-H et al. | Taiwan | 302 | Oral cavity, including lip | All stage | Lymphatic invasion | DFS |  |
| 2009 | Larsen et al. | Denmark | 144 | Oral cavity | All stage | ENE, Margin (cut-off 5 mm) | DSS |  |
| 2009 | Goodman et al. | USA | 339 | Oral tongue | All stage | Margin (clean vs. involved), LVI, PNI, DOI (cut-off 3 mm) | OS |  |
| 2007 | Liao et al. | Taiwan | 889 | Oral cavity, with lip | All stage | ENE | DRFS |  |
| 2005 | Kurokawa et al. | Japan | 124 | Oral tongue | All stage | DOI (cut-off 4 mm) | DFS |  |
| 2003 | Chen et al. | Taiwan | 59 | Oral cavity | All stage | ENE, DOI (cut-off 10 mm) | OS |  |
| 2000 | Ash et al. | Canada | 107 | Floor of mouth, lower alveolus, retromolar trigone | All stage | Margin (clean and close < 2 mm vs. involved), Bone invasion (no invasion and cortical vs. medullary) | DFS |  |
| 1999 | Högmo et al. | Sweden | 49 | Oral tongue | Early stage | Pattern of invasion (Jakobsson's system) | OS | |

DOI: depth of invasion, ENE: extranodal extension, PNI: perineural invasion, LVI: lymphovascular invasion, TSR: tumor-stroma ratio.

**Outcomes:** OS: overall survival, DSS: disease-specific survival, DFS: disease-free survival, LRFS: local recurrence-free survival, NRFS: neck recurrence-free survival, DRFS: distant recurrence-free survival, LRRFS: loco-regional recurrence free survival.

Adel M, Tsao CK, Wei FC, Chien HT, Lai CH, Liao CT, Wang HM, Fan KH, Kang CJ, Chang JT, Huang SF. Preoperative SCC Antigen, CRP Serum Levels, and Lymph Node Density in Oral Squamous Cell Carcinoma. Medicine (Baltimore). 2016 Apr;95(14):e3149. doi: 10.1097/MD.0000000000003149. PMID: 27057838; PMCID: PMC4998754

Agarwal JP, Kane S, Ghosh-Laskar S, Pilar A, Manik V, Oza N, Wagle P, Gupta T, Budrukkar A, Murthy V, Swain M. Extranodal extension in resected oral cavity squamous cell carcinoma: more to it than meets the eye. Laryngoscope. 2019 May;129(5):1130-1136. doi: 10.1002/lary.27508. Epub 2018 Nov 13. PMID: 30421434.

Aivazian K, Ebrahimi A, Low TH, Gao K, Clifford A, Shannon K, Clark JR, Gupta R. Perineural invasion in oral squamous cell carcinoma: quantitative subcategorisation of perineural invasion and prognostication. J Surg Oncol. 2015 Mar;111(3):352-8. doi: 10.1002/jso.23821. Epub 2014 Oct 31. PMID: 25363330.

Al Feghali KA, Ghanem AI, Burmeister C, Chang SS, Ghanem T, Keller C, Siddiqui F. Impact of smoking on pathological features in oral cavity squamous cell carcinoma. J Cancer Res Ther. 2019 Jul-Sep;15(3):582-588. doi: 10.4103/jcrt.JCRT_641_16. PMID: 31169224; PMCID: PMC7470189.

Almangush A, Coletta RD, Bello IO, Bitu C, Keski-Säntti H, Mäkinen LK, Kauppila JH, Pukkila M, Hagström J, Laranne J, Tommola S, Soini Y, Kosma VM, Koivunen P, Kowalski LP, Nieminen P, Grénman R, Leivo I, Salo T. A simple novel prognostic model for early stage oral tongue cancer. Int J Oral Maxillofac Surg. 2015 Feb;44(2):143-50. doi: 10.1016/j.ijom.2014.10.004. Epub 2014 Nov 11. PMID: 25457829.

Almangush A, Heikkinen I, Bakhti N, Mäkinen LK, Kauppila JH, Pukkila M, Hagström J, Laranne J, Soini Y, Kowalski LP, Grénman R, Haglund C, Mäkitie AA, Coletta RD, Leivo I, Salo T. Prognostic impact of tumour-stroma ratio in early-stage oral tongue cancers. Histopathology. 2018 Jun;72(7):1128-1135. doi: 10.1111/his.13481. Epub 2018 Mar 23. PMID: 29427291.

Almeida JR, Yao CMKL, Ziai H, Staibano P, Huang SH, Hosni A, Hope A, Bratman SV, Monteiro E, Gilbert RW, Brown DH, Gullane PJ, Irish JC, Su J, Xu W, Goldstein DP. Postoperative wound infections, neutrophil-to-lymphocyte ratio, and cancer recurrence in patients with oral cavity cancer undergoing surgical resection. Oral Oncol. 2019 Oct;97:23-30. doi: 10.1016/j.oraloncology.2019.07.023. Epub 2019 Aug 7. PMID: 31421467.

Amit M, Yen TC, Liao CT, Chaturvedi P, Agarwal JP, Kowalski LP, Ebrahimi A, Clark JR, Kreppel M, Zöller J, Fridman E, Bolzoni VA, Shah JP, Binenbaum Y, Patel SG, Gil Z; International Consortium for Outcome Research (ICOR) in Head and Neck Cancer. Improvement in survival of patients with oral cavity squamous cell carcinoma: An international collaborative study. Cancer. 2013 Dec 15;119(24):4242-8. doi: 10.1002/cncr.28357. Epub 2013 Sep 20. PMID: 24114787.

Ash CS, Nason RW, Abdoh AA, Cohen MA. Prognostic implications of mandibular invasion in oral cancer. Head Neck. 2000 Dec;22(8):794-8. doi: 10.1002/1097-0347(200012)22:8<794::aid-hed8>3.0.co;2-w. PMID: 11084640.

Bachar G, Goldstein DP, Barker E, Lea J, O'Sullivan B, Brown DH, Gullane PJ, Gilbert RW, Xu W, Su J, Irish JC. Squamous cell carcinoma of the buccal mucosa: outcomes of treatment in the modern era. Laryngoscope. 2012 Jul;122(7):1552-7. doi: 10.1002/lary.23296. Epub 2012 Jun 11. PMID: 22689284.

Bajwa MS, Houghton D, Java K, Triantafyllou A, Khattak O, Bekiroglu F, Schache AG, Brown JS, McCaul JA, Rogers SN, Lowe D, McMahon J, Shaw RJ. The relevance of surgical margins in clinically early oral squamous cell carcinoma. Oral Oncol. 2020 Nov;110:104913. doi: 10.1016/j.oraloncology.2020.104913. Epub 2020 Jul 22. PMID: 32711167.

Buchakjian MR, Ginader T, Tasche KK, Pagedar NA, Smith BJ, Sperry SM. Independent Predictors of Prognosis Based on Oral Cavity Squamous Cell Carcinoma Surgical Margins. Otolaryngol Head Neck Surg. 2018 Oct;159(4):675-682. doi: 10.1177/0194599818773070. Epub 2018 May 8. PMID: 29737907; PMCID: PMC6341475.

Camisasca DR, Silami MA, Honorato J, Dias FL, de Faria PA, Lourenço Sde Q. Oral squamous cell carcinoma: clinicopathological features in patients with and without recurrence. ORL J Otorhinolaryngol Relat Spec. 2011;73(3):170-6. doi: 10.1159/000328340. Epub 2011 May 27. PMID: 21625192.

Cassidy RJ, Switchenko JM, Jegadeesh N, Sayan M, Ferris MJ, Eaton BR, Higgins KA, Wadsworth JT, Magliocca KR, Saba NF, Beitler JJ. Association of Lymphovascular Space Invasion With Locoregional Failure and Survival in Patients With Node-Negative Oral Tongue Cancers. JAMA Otolaryngol Head Neck Surg. 2017 Apr 1;143(4):382-388. doi: 10.1001/jamaoto.2016.3795. PMID: 28097311; PMCID: PMC5398912.

Chang KP, Kao HK, Liang Y, Cheng MH, Chang YL, Liu SC, Lin YC, Ko TY, Lee YS, Tsai CL, Wang TH, Hao SP, Tsai CN. Overexpression of activin A in oral squamous cell carcinoma: association with poor prognosis and tumor progression. Ann Surg Oncol. 2010 Jul;17(7):1945-56. doi: 10.1245/s10434-010-0926-2. Epub 2010 Mar 23. PMID: 20309641.

Chang WC, Chang CF, Li YH, Yang CY, Su RY, Lin CK, Chen YW. A histopathological evaluation and potential prognostic implications of oral squamous cell carcinoma with adverse features. Oral Oncol. 2019 Aug;95:65-73. doi: 10.1016/j.oraloncology.2019.06.012. Epub 2019 Jun 10. PMID: 31345396.

Chang WC, Yang CY, Lin CS, Lin CK, Chen YW. Pretreatment body mass index as a prognostic predictor in patients with oral squamous cell carcinoma. Clin Oral Investig. 2020 Aug;24(8):2781-2788. doi: 10.1007/s00784-019-03141-2. Epub 2019 Nov 15. PMID: 31732879.

Chatzistefanou I, Lubek J, Markou K, Ord RA. The role of neck dissection and postoperative adjuvant radiotherapy in cN0 patients with PNI-positive squamous cell carcinoma of the oral cavity. Oral Oncol. 2014 Aug;50(8):753-8. doi: 10.1016/j.oraloncology.2014.05.005. Epub 2014 Jun 6. PMID: 24909939.

Chen HH, Chen IH, Liao CT, Wei FC, Lee LY, Huang SF. Preoperative circulating C-reactive protein levels predict pathological aggressiveness in oral squamous cell carcinoma: a retrospective clinical study. Clin Otolaryngol. 2011 Apr;36(2):147-53. doi: 10.1111/j.1749-4486.2011.02274.x. PMID: 21332670.

Chen IH, Chang JT, Liao CT, Wang HM, Hsieh LL, Cheng AJ. Prognostic significance of EGFR and Her-2 in oral cavity cancer in betel quid prevalent area cancer prognosis. Br J Cancer. 2003 Aug 18;89(4):681-6. doi: 10.1038/sj.bjc.6601171. PMID: 12915878; PMCID: PMC2376917.

Chen TC, Wang CP, Ko JY, Yang TL, Hsu CW, Yeh KA, Chang YL, Lou PJ. The impact of perineural invasion and/or lymphovascular invasion on the survival of early-stage oral squamous cell carcinoma patients. Ann Surg Oncol. 2013 Jul;20(7):2388-95. doi: 10.1245/s10434-013-2870-4. Epub 2013 Jan 30. PMID: 23361897.

Chen TC, Wu CT, Wang CP, Yang TL, Lou PJ, Ko JY, Chang YL. Significance of nuclear p-mTOR expression in advanced oral squamous cell carcinoma with extracapsular extension of lymph node metastases. Oral Oncol. 2015 May;51(5):493-9. doi: 10.1016/j.oraloncology.2015.02.093. Epub 2015 Mar 11. Erratum in: Oral Oncol. 2015 Oct;51(10):e79. PMID: 25769791.

Chen WC, Lai CH, Fang CC, Yang YH, Chen PC, Lee CP, Chen MF. Identification of High-Risk Subgroups of Patients With Oral Cavity Cancer in Need of Postoperative Adjuvant Radiotherapy or Chemo-Radiotherapy. Medicine (Baltimore). 2016 May;95(22):e3770. doi: 10.1097/MD.0000000000003770. PMID: 27258508; PMCID: PMC4900716.

Chen YW, Chen IL, Lin IC, Kao SY. Prognostic value of hypercalcaemia and leucocytosis in resected oral squamous cell carcinoma. Br J Oral Maxillofac Surg. 2014 May;52(5):425-31. doi: 10.1016/j.bjoms.2014.02.014. Epub 2014 Mar 18. PMID: 24656291.

Cheng HC, Yang CC, Kao SY, Wu TY, Wu CH. Evaluation of factors associated with the risk stratification of survivorship for stage IV squamous cell carcinoma of the oral cavity: A 10-year retrospective study. J Chin Med Assoc. 2020 May;83(5):491-499. doi: 10.1097/JCMA.0000000000000292. PMID: 32132387.

Cheng YJ, Tsai MH, Chiang CJ, Tsai ST, Liu TW, Lou PJ, Liao CT, Lin JC, Chang JT, Tsai MH, Chu PY, Leu YS, Tsai KY, Terng SD, Chien CY, Yang MH, Hao SP, Wang CC, Tsai MH, Chen HHW, Kuo C, Wu YH. Adjuvant radiotherapy after curative surgery for oral cavity squamous cell carcinoma and treatment effect of timing and duration on outcome-A Taiwan Cancer Registry national database analysis. Cancer Med. 2018 Jun 14;7(7):3073–83. doi: 10.1002/cam4.1611. Epub ahead of print. PMID: 29905028; PMCID: PMC6051157.

Chinn SB, Spector ME, Bellile EL, McHugh JB, Gernon TJ, Bradford CR, Wolf GT, Eisbruch A, Chepeha DB. Impact of perineural invasion in the pathologically N0 neck in oral cavity squamous cell carcinoma. Otolaryngol Head Neck Surg. 2013 Dec;149(6):893-9. doi: 10.1177/0194599813506867. Epub 2013 Oct 23. PMID: 24154744; PMCID: PMC4118458.

Cracchiolo JR, Xu B, Migliacci JC, Katabi N, Pfister DG, Lee NY, Patel SG, Ghossein RA, Wong RJ. Patterns of recurrence in oral tongue cancer with perineural invasion. Head Neck. 2018 Jun;40(6):1287-1295. doi: 10.1002/hed.25110. Epub 2018 Mar 9. PMID: 29522275; PMCID: PMC5980694.

D'Cruz AK, Vaish R, Kapre N, Dandekar M, Gupta S, Hawaldar R, Agarwal JP, Pantvaidya G, Chaukar D, Deshmukh A, Kane S, Arya S, Ghosh-Laskar S, Chaturvedi P, Pai P, Nair S, Nair D, Badwe R; Head and Neck Disease Management Group. Elective versus Therapeutic Neck Dissection in Node-Negative Oral Cancer. N Engl J Med. 2015 Aug 6;373(6):521-9. doi: 10.1056/NEJMoa1506007. Epub 2015 May 31. PMID: 26027881.

De Paz D, Chang KP, Kao HK, Lao WW, Huang YC, Chang YL, Huang Y. Clinical Implications of Tumor-Associated Tissue Eosinophilia in Tongue Squamous Cell Carcinoma. Laryngoscope. 2019 May;129(5):1123-1129. doi: 10.1002/lary.27413. Epub 2018 Aug 10. PMID: 30098046.

De Paz D, Young CK, Chien HT, Tsao CK, Fok CC, Fan KH, Liao CT, Wang HM, Kang CJ, Chang JT, Huang SF. Prognostic Roles of SCC Antigen, CRP and CYFRA 21-1 in Oral Cavity Squamous Cell Carcinoma. Anticancer Res. 2019 Apr;39(4):2025-2033. doi: 10.21873/anticanres.13313. PMID: 30952746.

Dillon JK, Brown CB, McDonald TM, Ludwig DC, Clark PJ, Leroux BG, Futran ND. How does the close surgical margin impact recurrence and survival when treating oral squamous cell carcinoma? J Oral Maxillofac Surg. 2015 Jun;73(6):1182-8. doi: 10.1016/j.joms.2014.12.014. Epub 2014 Dec 17. PMID: 25795179.

Ding D, Stokes W, Eguchi M, Hararah M, Sumner W, Amini A, Goddard J, Somerset H, Bradley C, McDermott J, Raben D, Karam SD. Association Between Lymph Node Ratio and Recurrence and Survival Outcomes in Patients With Oral Cavity Cancer. JAMA Otolaryngol Head Neck Surg. 2019 Jan 1;145(1):53-61. doi: 10.1001/jamaoto.2018.2974. PMID: 30452499; PMCID: PMC6439806.

Domingueti CB, Miwa KYM, Dourado MR, Sawazaki-Calone Í, Salo TA, Paranaíba LMR, Coletta RD. Prognostication for oral carcinomas based on two histological scoring systems (BD and iBD models). Oral Dis. 2021 May;27(4):894-899. doi: 10.1111/odi.13595. Epub 2020 Sep 7. PMID: 32772480.

Dourado MR, Miwa KYM, Hamada GB, Paranaíba LMR, Sawazaki-Calone Í, Domingueti CB, Ervolino de Oliveira C, Furlan ECB, Longo BC, Almangush A, Salo T, Coletta RD. Prognostication for oral squamous cell carcinoma patients based on the tumour-stroma ratio and tumour budding. Histopathology. 2020 May;76(6):906-918. doi: 10.1111/his.14070. Epub 2020 May 3. PMID: 31984527.

Durr ML, van Zante A, Li D, Kezirian EJ, Wang SJ. Oral tongue squamous cell carcinoma in never-smokers: analysis of clinicopathologic characteristics and survival. Otolaryngol Head Neck Surg. 2013 Jul;149(1):89-96. doi: 10.1177/0194599813482876. Epub 2013 Mar 26. PMID: 23533221.

Ebihara Y, Yoshida S, Nakahira M, Kogashiwa Y, Enoki Y, Kuba K, Inoue H, Minami K, Yasuda M, Sugasawa M. Importance of tumor budding grade as independent prognostic factor for early tongue squamous cell carcinoma. Head Neck. 2019 Jun;41(6):1809-1815. doi: 10.1002/hed.25614. Epub 2019 Jan 11. PMID: 30633412.

Ebrahimi A, Gil Z, Amit M, Yen TC, Liao CT, Chaturvedi P, Agarwal JP, Kowalski LP, Kreppel M, Cernea CR, Brandao J, Bachar G, Bolzoni Villaret A, Fliss D, Fridman E, Robbins KT, Shah JP, Patel SG, Clark JR. Primary tumor staging for oral cancer and a proposed modification incorporating depth of invasion: an international multicenter retrospective study. JAMA Otolaryngol Head Neck Surg. 2014 Dec;140(12):1138-48. doi: 10.1001/jamaoto.2014.1548. PMID: 25075712.

Ebrahimi A, Murali R, Gao K, Elliott MS, Clark JR. The prognostic and staging implications of bone invasion in oral squamous cell carcinoma. Cancer. 2011 Oct 1;117(19):4460-7. doi: 10.1002/cncr.26032. Epub 2011 Mar 22. PMID: 21437887.

Fan KH, Chen YC, Lin CY, Kang CJ, Lee LY, Huang SF, Liao CT, Ng SH, Wang HM, Chang JT. Postoperative radiotherapy with or without concurrent chemotherapy for oral squamous cell carcinoma in patients with three or more minor risk factors: a propensity score matching analysis. Radiat Oncol. 2017 Nov 22;12(1):184. doi: 10.1186/s13014-017-0910-0. PMID: 29166942; PMCID: PMC5700467.

Fan KH, Lin CY, Kang CJ, Lee LY, Huang SF, Liao CT, Chen IH, Ng SH, Wang HM, Chang JT. Postoperative concomitant chemoradiotherapy improved treatment outcomes of patients with oral cavity cancer with multiple-node metastases but no other major risk factors. PLoS One. 2014 Feb 24;9(2):e86922. doi: 10.1371/journal.pone.0086922. PMID: 24586259; PMCID: PMC3933339.

Fan KH, Wang HM, Kang CJ, Lee LY, Huang SF, Lin CY, Chen EY, Chen IH, Liao CT, Chang JT. Treatment results of postoperative radiotherapy on squamous cell carcinoma of the oral cavity: coexistence of multiple minor risk factors results in higher recurrence rates. Int J Radiat Oncol Biol Phys. 2010 Jul 15;77(4):1024-9. doi: 10.1016/j.ijrobp.2009.06.064. PMID: 20610038.

Feng Z, Niu LX, Yuan Y, Peng X, Guo CB. Risk factors and treatment of contralateral neck recurrence for unilateral oral squamous cell carcinoma: a retrospective study of 1482 cases. Oral Oncol. 2014 Nov;50(11):1081-8. doi: 10.1016/j.oraloncology.2014.08.003. Epub 2014 Aug 22. PMID: 25156479.

Fives C, Feeley L, O'Leary G, Sheahan P. Importance of lymphovascular invasion and invasive front on survival in floor of mouth cancer. Head Neck. 2016 Apr;38 Suppl 1:E1528-34. doi: 10.1002/hed.24273. Epub 2015 Nov 25. PMID: 26605755.

Fried D, Mullins B, Weissler M, Shores C, Zanation A, Hackman T, Shockley W, Hayes N, Chera BS. Prognostic significance of bone invasion for oral cavity squamous cell carcinoma considered T1/T2 by American joint committee on cancer size criteria. Head Neck. 2014 Jun;36(6):776-81. doi: 10.1002/hed.23367. Epub 2013 Jul 30. PMID: 23616341.

Ganly I, Goldstein D, Carlson DL, Patel SG, O'Sullivan B, Lee N, Gullane P, Shah JP. Long-term regional control and survival in patients with "low-risk," early stage oral tongue cancer managed by partial glossectomy and neck dissection without postoperative radiation: the importance of tumor thickness. Cancer. 2013 Mar 15;119(6):1168-76. doi: 10.1002/cncr.27872. Epub 2012 Nov 26. PMID: 23184439.

Ganly I, Patel S, Shah J. Early stage squamous cell cancer of the oral tongue--clinicopathologic features affecting outcome. Cancer. 2012 Jan 1;118(1):101-11. doi: 10.1002/cncr.26229. Epub 2011 Jun 29. PMID: 21717431.

Goodman M, Liu L, Ward K, Zhang J, Almon L, Su G, Berglund L, Chen A, Sinha UK, Young JL. Invasion characteristics of oral tongue cancer: frequency of reporting and effect on survival in a population-based study. Cancer. 2009 Sep 1;115(17):4010-20. doi: 10.1002/cncr.24459. PMID: 19551891.

Grimm M. Prognostic value of clinicopathological parameters and outcome in 484 patients with oral squamous cell carcinoma: microvascular invasion (V+) is an independent prognostic factor for OSCC. Clin Transl Oncol. 2012 Nov;14(11):870-80. doi: 10.1007/s12094-012-0867-2. Epub 2012 Jul 19. PMID: 22855158.

Hasegawa T, Shibuya Y, Takeda D, Iwata E, Saito I, Kakei Y, Sakakibara A, Akashi M, Minamikawa T, Komori T. Prognosis of oral squamous cell carcinoma patients with level IV/V metastasis: An observational study. J Craniomaxillofac Surg. 2017 Jan;45(1):145-149. doi: 10.1016/j.jcms.2016.10.011. Epub 2016 Oct 29. PMID: 27908710.

Hasmat S, Ebrahimi A, Gao K, Low TH, Palme C, Gupta R, Clark J. Multifocal perineural invasion is a better prognosticator than depth of invasion in oral squamous cell carcinoma. Head Neck. 2019 Nov;41(11):3992-3999. doi: 10.1002/hed.25940. Epub 2019 Sep 5. PMID: 31487105.

Hedbäck N, Jensen DH, Specht L, Fiehn AM, Therkildsen MH, Friis-Hansen L, Dabelsteen E, von Buchwald C. MiR-21 expression in the tumor stroma of oral squamous cell carcinoma: an independent biomarker of disease free survival. PLoS One. 2014 Apr 22;9(4):e95193. doi: 10.1371/journal.pone.0095193. PMID: 24755828; PMCID: PMC3995812.

Heerema MG, Melchers LJ, Roodenburg JL, Schuuring E, de Bock GH, van der Vegt B. Reproducibility and prognostic value of pattern of invasion scoring in low-stage oral squamous cell carcinoma. Histopathology. 2016 Feb;68(3):388-97. doi: 10.1111/his.12754. Epub 2015 Aug 3. PMID: 26062099.

Heiduschka G, Virk SA, Palme CE, Ch'ng S, Elliot M, Gupta R, Clark J. Margin to tumor thickness ratio - A predictor of local recurrence and survival in oral squamous cell carcinoma. Oral Oncol. 2016 Apr;55:49-54. doi: 10.1016/j.oraloncology.2016.01.010. Epub 2016 Feb 6. PMID: 26861256.

Heikkinen I, Bello IO, Wahab A, Hagström J, Haglund C, Coletta RD, Nieminen P, Mäkitie AA, Salo T, Leivo I, Almangush A. Assessment of Tumor-infiltrating Lymphocytes Predicts the Behavior of Early-stage Oral Tongue Cancer. Am J Surg Pathol. 2019 Oct;43(10):1392-1396. doi: 10.1097/PAS.0000000000001323. PMID: 31290758.

Ho AS, Kim S, Tighiouart M, Gudino C, Mita A, Scher KS, Laury A, Prasad R, Shiao SL, Van Eyk JE, Zumsteg ZS. Metastatic Lymph Node Burden and Survival in Oral Cavity Cancer. J Clin Oncol. 2017 Nov 1;35(31):3601-3609. doi: 10.1200/JCO.2016.71.1176. Epub 2017 Sep 7. PMID: 28880746; PMCID: PMC5791830.

Ho YY, Wu TY, Cheng HC, Yang CC, Wu CH. The significance of tumor budding in oral cancer survival and its relevance to the eighth edition of the American Joint Committee on Cancer staging system. Head Neck. 2019 Sep;41(9):2991-3001. doi: 10.1002/hed.25780. Epub 2019 Apr 23. PMID: 31012518.

Hoffmann C, Vacher S, Sirven P, Lecerf C, Massenet L, Moreira A, Surun A, Schnitzler A, Klijanienko J, Mariani O, Jeannot E, Badois N, Lesnik M, Choussy O, Le Tourneau C, Guillot-Delost M, Kamal M, Bieche I, Soumelis V. MMP2 as an independent prognostic stratifier in oral cavity cancers. Oncoimmunology. 2020 May 13;9(1):1754094. doi: 10.1080/2162402X.2020.1754094. PMID: 32934875; PMCID: PMC7466851.

Högmo A, Kuylenstierna R, Lindholm J, Munck-Wikland E. Predictive value of malignancy grading systems, DNA content, p53, and angiogenesis for stage I tongue carcinomas. J Clin Pathol. 1999 Jan;52(1):35-40. doi: 10.1136/jcp.52.1.35. PMID: 10343610; PMCID: PMC501005.

Hori Y, Kubota A, Yokose T, Furukawa M, Matsushita T, Oridate N. Association between pathological invasion patterns and late lymph node metastases in patients with surgically treated clinical No early oral tongue carcinoma. Head Neck. 2020 Feb;42(2):238-243. doi: 10.1002/hed.25994. Epub 2019 Oct 24. PMID: 31647162.

Hori Y, Kubota A, Yokose T, Furukawa M, Matsushita T, Takita M, Mitsunaga S, Mizoguchi N, Nonaka T, Nakayama Y, Oridate N. Predictive Significance of Tumor Depth and Budding for Late Lymph Node Metastases in Patients with Clinical N0 Early Oral Tongue Carcinoma. Head Neck Pathol. 2017 Dec;11(4):477-486. doi: 10.1007/s12105-017-0814-1. Epub 2017 Apr 3. PMID: 28374102; PMCID: PMC5677061.

Hosni A, McMullen C, Huang SH, Xu W, Su J, Bayley A, Bratman SV, Cho J, Giuliani M, Kim J, Ringash J, Waldron J, Spreafico A, Weinreb I, de Almeida JR, Brown DH, Irish JC, O'Sullivan B, Goldstein DP, Hope A. Lymph node ratio relationship to regional failure and distant metastases in oral cavity cancer. Radiother Oncol. 2017 Aug;124(2):225-231. doi: 10.1016/j.radonc.2017.06.018. PMID: 28838425.

Huang SF, Chien HT, Chuang WY, Lai CH, Cheng SD, Liao CT, Wang HM. Epidermal growth factor receptor intron-1 CA repeat polymorphism on protein expression and clinical outcome in Taiwanese oral squamous cell carcinoma. Sci Rep. 2017 Jul 10;7(1):4963. doi: 10.1038/s41598-017-04954-5. PMID: 28694429; PMCID: PMC5504053.

Jan JC, Hsu WH, Liu SA, Wong YK, Poon CK, Jiang RS, Jan JS, Chen IF. Prognostic factors in patients with buccal squamous cell carcinoma: 10-year experience. J Oral Maxillofac Surg. 2011 Feb;69(2):396-404. doi: 10.1016/j.joms.2010.05.017. PMID: 21238843.

Jang JY, Choi N, Ko YH, Chung MK, Son YI, Baek CH, Baek KH, Jeong HS. Differential Impact of Close Surgical Margin on Local Recurrence According to Primary Tumor Size in Oral Squamous Cell Carcinoma. Ann Surg Oncol. 2017 Jun;24(6):1698-1706. doi: 10.1245/s10434-016-5497-4. Epub 2016 Aug 12. PMID: 27519352.

Jardim JF, Francisco AL, Gondak R, Damascena A, Kowalski LP. Prognostic impact of perineural invasion and lymphovascular invasion in advanced stage oral squamous cell carcinoma. Int J Oral Maxillofac Surg. 2015 Jan;44(1):23-8. doi: 10.1016/j.ijom.2014.10.006. Epub 2014 Nov 7. PMID: 25457832.

Jing Y, Yang Y, Hao F, Song Y, Zhang X, Zhang Y, Huang X, Hu Q, Ni Y. Higher Ki67 expression in fibroblast like cells at invasive front indicates better clinical outcomes in oral squamous cell carcinoma patients. Biosci Rep. 2018 Nov 20;38(6):BSR20181271. doi: 10.1042/BSR20181271. PMID: 30341240; PMCID: PMC6246770.

Kikuchi M, Yamashita D, Hara S, Takebayashi S, Hamaguchi K, Mizuno K, Omori K, Shinohara S. Clinical significance of tumor-associated immune cells in patients with oral squamous cell carcinoma. Head Neck. 2021 Feb;43(2):534-543. doi: 10.1002/hed.26498. Epub 2020 Oct 7. PMID: 33029887.

Kim KY, Zhang X, Kim SM, Lee BD, Cha IH. A combined prognostic factor for improved risk stratification of patients with oral cancer. Oral Dis. 2017 Jan;23(1):91-96. doi: 10.1111/odi.12579. Epub 2016 Oct 6. PMID: 27588367.

Kong YH, Syed Zanaruddin SN, Lau SH, Ramanathan A, Kallarakkal TG, Vincent-Chong VK, Wan Mustafa WM, Abraham MT, Abdul Rahman ZA, Zain RB, Cheong SC. Co-Expression of TWIST1 and ZEB2 in Oral Squamous Cell Carcinoma Is Associated with Poor Survival. PLoS One. 2015 Jul 27;10(7):e0134045. doi: 10.1371/journal.pone.0134045. PMID: 26214683; PMCID: PMC4516250.

Kozak MM, Shah J, Chen M, Schaberg K, von Eyben R, Chen JJ, Bui T, Kong C, Kaplan M, Divi V, Hara W. Depth of invasion alone as a prognostic factor in low-risk early-stage oral cavity carcinoma. Laryngoscope. 2019 Sep;129(9):2082-2086. doi: 10.1002/lary.27753. Epub 2019 Jan 2. PMID: 30604435.

Kurihara-Shimomura M, Sasahira T, Shimomura H, Kirita T. Peroxidan Plays a Tumor-Promoting Role in Oral Squamous Cell Carcinoma. Int J Mol Sci. 2020 Jul 30;21(15):5416. doi: 10.3390/ijms21155416. PMID: 32751434; PMCID: PMC7432510.

Kurita H, Nakanishi Y, Nishizawa R, Xiao T, Kamata T, Koike T, Kobayashi H. Impact of different surgical margin conditions on local recurrence of oral squamous cell carcinoma. Oral Oncol. 2010 Nov;46(11):814-7. doi: 10.1016/j.oraloncology.2010.08.014. PMID: 20920879.

Kurokawa H, Zhang M, Matsumoto S, Yamashita Y, Tomoyose T, Tanaka T, Fukuyama H, Takahashi T. The high prognostic value of the histologic grade at the deep invasive front of tongue squamous cell carcinoma. J Oral Pathol Med. 2005 Jul;34(6):329-33. doi: 10.1111/j.1600-0714.2005.00244.x. PMID: 15946179.

Lai TY, Hu YW, Liu YM, Chen YW, Shiau CY, Chu PY, Tai SK, Lo WL, Wu CH, Wang LW. The pattern of failure and predictors of locoregional control in lateralized buccogingival cancer after postoperative radiation therapy. J Chin Med Assoc. 2017 Sep;80(9):569-574. doi: 10.1016/j.jcma.2017.06.002. Epub 2017 Jul 4. PMID: 28687157.

Lapke N, Lu YJ, Liao CT, Lee LY, Lin CY, Wang HM, Ng SH, Chen SJ, Yen TC. Missense mutations in the TP53 DNA-binding domain predict outcomes in patients with advanced oral cavity squamous cell carcinoma. Oncotarget. 2016 Jul 12;7(28):44194-44210. doi: 10.18632/oncotarget.9925. PMID: 27283772; PMCID: PMC5190089.

Larsen SR, Johansen J, Sørensen JA, Krogdahl A. The prognostic significance of histological features in oral squamous cell carcinoma. J Oral Pathol Med. 2009 Sep;38(8):657-62. doi: 10.1111/j.1600-0714.2009.00797.x. Epub 2009 Jun 25. PMID: 19563504.

Lau L, Eu D, Loh T, Ahmed Q, Lim CM. Histopathologic prognostic indices in tongue squamous cell carcinoma. Eur Arch Otorhinolaryngol. 2021 Jul;278(7):2461-2471. doi: 10.1007/s00405-020-06329-5. Epub 2020 Sep 8. PMID: 32897441.

Lee CC, Huang CY, Lin YS, Chang KP, Chi CC, Lin MY, Su HH, Chang TS, Chen HC, Yang CC. Prognostic Performance of a New Staging Category to Improve Discrimination of Disease-Specific Survival in Nonmetastatic Oral Cancer. JAMA Otolaryngol Head Neck Surg. 2017 Apr 1;143(4):395-402. doi: 10.1001/jamaoto.2016.3802. PMID: 28125757.

Lee LY, De Paz D, Lin CY, Fan KH, Wang HM, Hsieh CH, Lee LA, Yen TC, Liao CT, Yeh CH, Kang CJ. Prognostic impact of extratumoral perineural invasion in patients with oral cavity squamous cell carcinoma. Cancer Med. 2019 Oct;8(14):6185-6194. doi: 10.1002/cam4.2392. Epub 2019 Jul 10. PMID: 31290283; PMCID: PMC6797567.

Lee JR, Roh JL, Lee SM, Park Y, Cho KJ, Choi SH, Nam SY, Kim SY. Overexpression of cysteine-glutamate transporter and CD44 for prediction of recurrence and survival in patients with oral cavity squamous cell carcinoma. Head Neck. 2018 Nov;40(11):2340-2346. doi: 10.1002/hed.25331. Epub 2018 Oct 10. PMID: 30303590.

Li Y, Bai S, Carroll W, Dayan D, Dort JC, Heller K, Jour G, Lau H, Penner C, Prystowsky M, Rosenthal E, Schlecht NF, Smith RV, Urken M, Vered M, Wang B, Wenig B, Negassa A, Brandwein-Gensler M. Validation of the risk model: high-risk classification and tumor pattern of invasion predict outcome for patients with low-stage oral cavity squamous cell carcinoma. Head Neck Pathol. 2013 Sep;7(3):211-23. doi: 10.1007/s12105-012-0412-1. Epub 2012 Dec 19. PMID: 23250819; PMCID: PMC3738758.

Liao CT, Wang HM, Chang JT, Ng SH, Hsueh C, Lee LY, Lin CH, Chen IH, Huang SF, Yen TC. Analysis of risk factors for distant metastases in squamous cell carcinoma of the oral cavity. Cancer. 2007 Oct 1;110(7):1501-8. doi: 10.1002/cncr.22959. PMID: 17868119.

Lien KH, Padua PFC, Tay ZY, Kao HK, Hung SY, Huang Y, Tsang NM, Chang KP. Influence of Hyperglycemia on Treatment Outcomes of Oral Cavity Squamous Cell Carcinoma. J Oral Maxillofac Surg. 2020 Jun;78(6):935-942. doi: 10.1016/j.joms.2020.01.018. Epub 2020 Jan 23. PMID: 32081693.

Lin CY, Lin TY, Wang HM, Huang SF, Fan KH, Liao CT, Chen IH, Lee LY, Li YL, Chen YJ, Cheng AJ, Chang JT. GP96 is over-expressed in oral cavity cancer and is a poor prognostic indicator for patients receiving radiotherapy. Radiat Oncol. 2011 Oct 12;6:136. doi: 10.1186/1748-717X-6-136. PMID: 21992474; PMCID: PMC3214142.

Lin NC, Hsu JT, Tsai KY. Survival and clinicopathological characteristics of different histological grades of oral cavity squamous cell carcinoma: A single-center retrospective study. PLoS One. 2020 Aug 25;15(8):e0238103. doi: 10.1371/journal.pone.0238103. PMID: 32841288; PMCID: PMC7447052.

Lin YT, Chien CY, Lu CT, Lou SD, Lu H, Huang CC, Fang FM, Li SH, Huang TL, Chuang HC. Triple-positive pathologic findings in oral cavity cancer are related to a dismal prognosis. Laryngoscope. 2015 Sep;125(9):E300-5. doi: 10.1002/lary.25463. Epub 2015 Jul 7. PMID: 26152458.

Ling W, Mijiti A, Moming A. Survival pattern and prognostic factors of patients with squamous cell carcinoma of the tongue: a retrospective analysis of 210 cases. J Oral Maxillofac Surg. 2013 Apr;71(4):775-85. doi: 10.1016/j.joms.2012.09.026. Epub 2012 Dec 23. PMID: 23265849.

Liu KY, Durham JS, Wu J, Anderson DW, Prisman E, Poh CF. Nodal Disease Burden for Early-Stage Oral Cancer. JAMA Otolaryngol Head Neck Surg. 2016 Nov 1;142(11):1111-1119. doi: 10.1001/jamaoto.2016.2241. PMID: 27560665.

Liu SA, Wang CC, Jiang RS, Lee FY, Lin WJ, Lin JC. Pathological features and their prognostic impacts on oral cavity cancer patients among different subsites - A singe institute's experience in Taiwan. Sci Rep. 2017 Aug 7;7(1):7451. doi: 10.1038/s41598-017-08022-w. PMID: 28785002; PMCID: PMC5547072.

Lu Z, Ding L, Ding H, Hao F, Pu Y, Wang Y, Chen S, Yang Y, Zhao X, Huang X, Zhang L, Wang Z, Hu Q, Ni Y. Tumor cell-derived TGF-β at tumor center independently predicts recurrence and poor survival in oral squamous cell carcinoma. J Oral Pathol Med. 2019 Sep;48(8):696-704. doi: 10.1111/jop.12888. Epub 2019 Jun 17. PMID: 31141218.

Luryi AL, Chen MM, Mehra S, Roman SA, Sosa JA, Judson BL. Treatment Factors Associated With Survival in Early-Stage Oral Cavity Cancer: Analysis of 6830 Cases From the National Cancer Data Base. JAMA Otolaryngol Head Neck Surg. 2015 Jul;141(7):593-8. doi: 10.1001/jamaoto.2015.0719. PMID: 25974757.

Manjula BV, Augustine S, Selvam S, Mohan AM. Prognostic and predictive factors in gingivo buccal complex squamous cell carcinoma: role of tumor budding and pattern of invasion. Indian J Otolaryngol Head Neck Surg. 2015 Mar;67(Suppl 1):98-104. doi: 10.1007/s12070-014-0787-2. Epub 2014 Oct 31. PMID: 25621262; PMCID: PMC4298608.

Marinelli LM, Chatzopoulos K, Marinelli JP, Chen TY, Collins AR, Sotiriou S, Raslan SW, Vêncio EF, Price DL, Garcia JJ, Janus JR. Clinicopathologic predictors of survival in buccal squamous cell carcinoma. J Oral Pathol Med. 2020 Oct;49(9):857-864. doi: 10.1111/jop.13046. Epub 2020 Jun 18. PMID: 32449549.

Marsh D, Suchak K, Moutasim KA, Vallath S, Hopper C, Jerjes W, Upile T, Kalavrezos N, Violette SM, Weinreb PH, Chester KA, Chana JS, Marshall JF, Hart IR, Hackshaw AK, Piper K, Thomas GJ. Stromal features are predictive of disease mortality in oral cancer patients. J Pathol. 2011 Mar;223(4):470-81. doi: 10.1002/path.2830. Epub 2011 Jan 5. PMID: 21294121

Mascitti M, Tempesta A, Togni L, Capodiferro S, Troiano G, Rubini C, Maiorano E, Santarelli A, Favia G, Limongelli L. Histological features and survival in young patients with HPV-negative oral squamous cell carcinoma. Oral Dis. 2020 Nov;26(8):1640-1648. doi: 10.1111/odi.13479. Epub 2020 Jul 31. PMID: 32531817.

Mascitti M, Zhurakivska K, Togni L, Caponio VCA, Almangush A, Balercia P, Balercia A, Rubini C, Lo Muzio L, Santarelli A, Troiano G. Addition of the tumour-stroma ratio to the 8th edition American Joint Committee on Cancer staging system improves survival prediction for patients with oral tongue squamous cell carcinoma. Histopathology. 2020 Nov;77(5):810-822. doi: 10.1111/his.14202. Epub 2020 Sep 12. PMID: 32633006.

Michikawa C, Uzawa N, Sato H, Ohyama Y, Okada N, Amagasa T. Epidermal growth factor receptor gene copy number aberration at the primary tumour is significantly associated with extracapsular spread in oral cancer. Br J Cancer. 2011 Mar 1;104(5):850-5. doi: 10.1038/bjc.2011.22. Epub 2011 Feb 8. PMID: 21304522; PMCID: PMC3048213.

Miyazaki A, Nakai H, Sonoda T, Hirohashi Y, Kaneko MK, Kato Y, Sawa Y, Hiratsuka H. LpMab-23-recognizing cancer-type podoplanin is a novel predictor for a poor prognosis of early stage tongue cancer. Oncotarget. 2018 Apr 20;9(30):21156-21165. doi: 10.18632/oncotarget.24986. PMID: 29765527; PMCID: PMC5940393.

Mizrachi A, Migliacci JC, Montero PH, McBride S, Shah JP, Patel SG, Ganly I. Neck recurrence in clinically node-negative oral cancer: 27-year experience at a single institution. Oral Oncol. 2018 Mar;78:94-101. doi: 10.1016/j.oraloncology.2018.01.020. Epub 2018 Feb 20. PMID: 29496065; PMCID: PMC5836807.

Mneimneh WS, Xu B, Ghossein C, Alzumaili B, Sethi S, Ganly I, Khimraj A, Dogan S, Katabi N. Clinicopathologic Characteristics of Young Patients with Oral Squamous Cell Carcinoma. Head Neck Pathol. 2021 Apr 2. doi: 10.1007/s12105-021-01320-w. Epub ahead of print. PMID: 33797696.

Monteiro LS, Amaral JB, Vizcaíno JR, Lopes CA, Torres FO. A clinical-pathological and survival study of oral squamous cell carcinomas from a population of the North of Portugal. Med Oral Patol Oral Cir Bucal. 2014 Mar 1;19(2):e120-6. doi: 10.4317/medoral.19090. PMID: 24121907; PMCID: PMC4015041.

Montero PH, Yu C, Palmer FL, Patel PD, Ganly I, Shah JP, Shaha AR, Boyle JO, Kraus DH, Singh B, Wong RJ, Morris LG, Kattan MW, Patel SG. Nomograms for preoperative prediction of prognosis in patients with oral cavity squamous cell carcinoma. Cancer. 2014 Jan 15;120(2):214-21. doi: 10.1002/cncr.28407. Epub 2013 Oct 25. PMID: 24399417.

Moreira A, Poulet A, Masliah-Planchon J, Lecerf C, Vacher S, Larbi Chérif L, Dupain C, Marret G, Girard E, Syx L, Hoffmann C, Jeannot E, Klijanienko J, Guillou I, Mariani O, Dubray-Vautrin A, Badois N, Lesnik M, Choussy O, Calugaru V, Borcoman E, Baulande S, Legoix P, Albaud B, Servant N, Bieche I, Le Tourneau C, Kamal M. Prognostic value of tumor mutational burden in patients with oral cavity squamous cell carcinoma treated with upfront surgery. ESMO Open. 2021 Aug;6(4):100178. doi: 10.1016/j.esmoop.2021.100178. Epub 2021 Jun 9. PMID: 34118772; PMCID: PMC8207209.

Mücke T, Hölzle F, Wagenpfeil S, Wolff KD, Kesting M. The role of tumor invasion into the mandible of oral squamous cell carcinoma. J Cancer Res Clin Oncol. 2011 Jan;137(1):165-71. doi: 10.1007/s00432-010-0870-3. Epub 2010 Mar 31. PMID: 20354727.

Mücke T, Kanatas A, Ritschl LM, Koerdt S, Tannapfel A, Wolff KD, Loeffelbein D, Kesting M. Tumor thickness and risk of lymph node metastasis in patients with squamous cell carcinoma of the tongue. Oral Oncol. 2016 Feb;53:80-4. doi: 10.1016/j.oraloncology.2015.11.010. Epub 2015 Nov 25. PMID: 26625728.

Nagam SL, Katta S, Prasad VV. Gender specific association of TP53 polymorphisms (EX4 215G>C Arg72Pro, IVS3+40-41ins16, and IVS6+62G>A), with risk of oral cancer subtypes and overall survival of the patients. Mol Carcinog. 2017 Mar;56(3):895-912. doi: 10.1002/mc.22543. Epub 2016 Aug 31. PMID: 27532290.

Nair D, Mair M, Singhvi H, Mishra A, Nair S, Agrawal J, Chaturvedi P. Perineural invasion: Independent prognostic factor in oral cancer that warrants adjuvant treatment. Head Neck. 2018 Aug;40(8):1780-1787. doi: 10.1002/hed.25170. Epub 2018 Apr 29. PMID: 29707840.

Nakao Y, Yamada S, Yanamoto S, Tomioka T, Naruse T, Ikeda T, Kurita H, Umeda M. Natriuretic peptide receptor A is related to the expression of vascular endothelial growth factors A and C, and is associated with the invasion potential of tongue squamous cell carcinoma. Int J Oral Maxillofac Surg. 2017 Oct;46(10):1237-1242. doi: 10.1016/j.ijom.2017.04.022. Epub 2017 May 15. PMID: 28521969.

Nguyen E, McKenzie J, Clarke R, Lou S, Singh T. The Indications for Elective Neck Dissection in T1N0M0 Oral Cavity Squamous Cell Carcinoma. J Oral Maxillofac Surg. 2021 Aug;79(8):1779-1793. doi: 10.1016/j.joms.2021.01.042. Epub 2021 Feb 24. PMID: 33744243.

Niu LX, Feng ZE, Wang DC, Zhang JY, Sun ZP, Guo CB. Prognostic factors in mandibular gingival squamous cell carcinoma: A 10-year retrospective study. Int J Oral Maxillofac Surg. 2017 Feb;46(2):137-143. doi: 10.1016/j.ijom.2016.09.014. Epub 2016 Oct 28. PMID: 28029423.

Oliver JR, Wu SP, Chang CM, Roden DF, Wang B, Hu KS, Schreiber D, Givi B. Survival of oral tongue squamous cell carcinoma in young adults. Head Neck. 2019 Sep;41(9):2960-2968. doi: 10.1002/hed.25772. Epub 2019 Apr 15. PMID: 30985036.

Padma R, Kalaivani A, Sundaresan S, Sathish P. The relationship between histological differentiation and disease recurrence of primary oral squamous cell carcinoma. J Oral Maxillofac Pathol. 2017 Sep-Dec;21(3):461. doi: 10.4103/jomfp.JOMFP_241_16. PMID: 29391735; PMCID: PMC5763883.

Parekh D, Kukreja P, Mallick I, Roy P. Worst pattern of invasion - type 4 (WPOI-4) and Lymphocyte host response should be mandatory reporting criteria for oral cavity squamous cell carcinoma: A re-look at the American Joint Committee of Cancer (AJCC) minimum dataset. Indian J Pathol Microbiol. 2020 Oct-Dec;63(4):527-533. doi: 10.4103/IJPM.IJPM_662_19. PMID: 33154300.

Perisanidis C, Kornek G, Pöschl PW, Holzinger D, Pirklbauer K, Schopper C, Ewers R. High neutrophil-to-lymphocyte ratio is an independent marker of poor disease-specific survival in patients with oral cancer. Med Oncol. 2013 Mar;30(1):334. doi: 10.1007/s12032-012-0334-5. Epub 2013 Jan 6. PMID: 23292862.

Petera J, Sirák I, Tuček L, Hodek M, Paluska P, Kašaová L, Paulíková S, Vošmik M, Doležalová H, Cvanova M, Halamka M, Laco J. Predicting factors for locoregional failure of high-dose-rate brachytherapy for early-stage oral cancer. Per Med. 2012 Nov;9(8):879-887. doi: 10.2217/pme.12.95. PMID: 29776235.

Petrovic I, Montero PH, Migliacci JC, Palmer FL, Ganly I, Patel SG, Shah JP. Influence of bone invasion on outcomes after marginal mandibulectomy in squamous cell carcinoma of the oral cavity. J Craniomaxillofac Surg. 2017 Feb;45(2):252-257. doi: 10.1016/j.jcms.2016.11.017. Epub 2016 Nov 30. PMID: 28011180; PMCID: PMC5293664.

Pinto FR, de Matos LL, Palermo FC, Kulcsar MA, Cavalheiro BG, de Mello ES, Alves VA, Cernea CR, Brandão LG. Tumor thickness as an independent risk factor of early recurrence in oral cavity squamous cell carcinoma. Eur Arch Otorhinolaryngol. 2014 Jun;271(6):1747-54. doi: 10.1007/s00405-013-2704-9. Epub 2013 Sep 27. PMID: 24071858.

Pu YM, Yang Y, Wang YJ, Ding L, Huang XF, Wang ZY, Ni YH, Hu QG. Postoperative radiotherapy is dispensable for OSCC patients with micrometastases in lymph nodes. Virchows Arch. 2018 May;472(5):797-805. doi: 10.1007/s00428-018-2351-z. Epub 2018 Apr 9. PMID: 29629513.

Quinlan-Davidson SR, Mohamed ASR, Myers JN, Gunn GB, Johnson FM, Skinner H, Beadle BM, Gillenwater AM, Phan J, Frank SJ, William WN, Wong AJ, Lai SY, Fuller CD, Morrison WH, Rosenthal DI, Garden AS. Outcomes of oral cavity cancer patients treated with surgery followed by postoperative intensity modulated radiation therapy. Oral Oncol. 2017 Sep;72:90-97. doi: 10.1016/j.oraloncology.2017.07.002. Epub 2017 Jul 16. PMID: 28797467; PMCID: PMC5796661.

Rajappa SK, Maheshwari U, Ram D, Koyyala VPB, Mandal G, Goyal S, Kumar R, Dewan AK. Early oral cavity cancer: The prognostic factors and impact of adjuvant radiation on survival. Head Neck. 2019 Oct;41(10):3577-3583. doi: 10.1002/hed.25882. Epub 2019 Jul 24. PMID: 31339609.

Rodrigues RM, Bernardo VG, Da Silva SD, Camisasca DR, Faria PAS, Dias FL, Pinto LFR, Albano RM, Bergmann A, Lourenço SQC. How pathological criteria can impact prognosis of tongue and floor of the mouth squamous cell carcinoma. J Appl Oral Sci. 2019 Nov 28;28:e20190198. doi: 10.1590/1678-7757-2019-0198. PMID: 31800876; PMCID: PMC6886392.

Roh JL, Lee H, Choi SH, Nam SY, Kim SY. Tumor-related leukocytosis predictive of recurrence and survival in patients with oral cavity squamous cell carcinoma. Oral Dis. 2019 Sep;25(6):1511-1518. doi: 10.1111/odi.13138. Epub 2019 Jun 25. PMID: 31165559.

Rubin SJ, Gurary EB, Qureshi MM, Salama AR, Ezzat WH, Jalisi S, Truong MT. Stage II Oral Tongue Cancer: Survival Impact of Adjuvant Radiation Based on Depth of Invasion. Otolaryngol Head Neck Surg. 2019 Jan;160(1):77-84. doi: 10.1177/0194599818779907. Epub 2018 Jun 26. PMID: 29944460.

Safi AF, Grandoch A, Nickenig HJ, Zöller JE, Kreppel M. The importance of lymph node ratio for locoregional recurrence of squamous cell carcinoma of the tongue. J Craniomaxillofac Surg. 2017 Jul;45(7):1058-1061. doi: 10.1016/j.jcms.2017.04.008. Epub 2017 Apr 22. PMID: 28529103.

Safi AF, Grochau K, Drebber U, Schick V, Thiele O, Backhaus T, Nickenig HJ, Zöller JE, Kreppel M. A novel histopathological scoring system for patients with oral squamous cell carcinoma. Clin Oral Investig. 2019 Oct;23(10):3759-3765. doi: 10.1007/s00784-019-02804-4. Epub 2019 Jan 23. PMID: 30673863.

Safi AF, Kauke M, Grandoch A, Nickenig HJ, Zöller JE, Kreppel M. Analysis of clinicopathological risk factors for locoregional recurrence of oral squamous cell carcinoma - Retrospective analysis of 517 patients. J Craniomaxillofac Surg. 2017 Oct;45(10):1749-1753. doi: 10.1016/j.jcms.2017.07.012. Epub 2017 Jul 27. PMID: 28823597.

Safi AF, Kauke M, Grandoch A, Nickenig HJ, Zöller J, Kreppel M. The importance of lymph node ratio for patients with mandibular infiltration of oral squamous cell carcinoma. J Craniomaxillofac Surg. 2018 Jun;46(6):1007-1012. doi: 10.1016/j.jcms.2018.03.021. Epub 2018 Apr 5. PMID: 29709331.

Sakata J, Yamana K, Yoshida R, Matsuoka Y, Kawahara K, Arita H, Nakashima H, Nagata M, Hirosue A, Kawaguchi S, Gohara S, Nagao Y, Hiraki A, Shinohara M, Toya R, Murakami R, Nakayama H. Tumor budding as a novel predictor of occult metastasis in cT2N0 tongue squamous cell carcinoma. Hum Pathol. 2018 Jun;76:1-8. doi: 10.1016/j.humpath.2017.12.021. Epub 2018 Jan 4. PMID: 29307623.

Sambasivan K, Sassoon I, Thavaraj S, Kennedy R, Doss G, Michaelidou A, Odell E, Sandison A, Hall G, Morgan P, Collins LHC, Lyons A, Cascarini L, Fry A, Oakley R, Simo R, Jeannon JP, Petkar I, Reis Ferreira M, Kong A, Lei M, Guerrero Urbano T. TNM 8 staging is a better prognosticator than TNM 7 for patients with locally advanced oral cavity squamous cell carcinoma treated with surgery and post-operative radiotherapy. Radiother Oncol. 2021 Jul;160:54-60. doi: 10.1016/j.radonc.2021.04.003. Epub 2021 May 3. PMID: 33845044.

Satgunaseelan L, Virk SA, Lum T, Gao K, Clark JR, Gupta R. p16 expression independent of human papillomavirus is associated with lower stage and longer disease-free survival in oral cavity squamous cell carcinoma. Pathology. 2016 Aug;48(5):441-8. doi: 10.1016/j.pathol.2016.03.015. Epub 2016 Jun 28. PMID: 27370365.

Sharma K, Ahlawat P, Gairola M, Tandon S, Sachdeva N, Sharief MI. Prognostic factors, failure patterns and survival analysis in patients with resectable oral squamous cell carcinoma of the tongue. Radiat Oncol J. 2019 Jun;37(2):73-81. doi: 10.3857/roj.2018.00577. Epub 2019 Jun 30. PMID: 31266288; PMCID: PMC6610009.

Shim SJ, Cha J, Koom WS, Kim GE, Lee CG, Choi EC, Keum KC. Clinical outcomes for T1-2N0-1 oral tongue cancer patients underwent surgery with and without postoperative radiotherapy. Radiat Oncol. 2010 May 27;5:43. doi: 10.1186/1748-717X-5-43. PMID: 20504371; PMCID: PMC2887888.

Shinagawa K, Yanamoto S, Naruse T, Kawakita A, Morishita K, Sakamoto Y, Rokutanda S, Umeda M. Clinical Roles of Interleukin-6 and STAT3 in Oral Squamous Cell Carcinoma. Pathol Oncol Res. 2017 Apr;23(2):425-431. doi: 10.1007/s12253-016-0134-x. Epub 2016 Oct 15. PMID: 27744625.

Shukla NK, Deo SVS, Garg PK, Manjunath NML, Bhaskar S, Sreenivas V. Operable Oral Tongue Squamous Cell Cancer: 15 Years Experience at a Tertiary Care Center in North India. Indian J Surg Oncol. 2018 Mar;9(1):15-23. doi: 10.1007/s13193-017-0658-x. Epub 2017 May 11. PMID: 29563729; PMCID: PMC5856685.

Slieker FJB, de Bree R, Van Cann EM. Predicting individualized mortality probabilities for patients with squamous cell carcinoma of the maxilla: Novel models with clinical and histopathological predictors. Head Neck. 2019 Oct;41(10):3584-3593. doi: 10.1002/hed.25879. Epub 2019 Jul 26. PMID: 31347740.

Spoerl S, Gerken M, Mamilos A, Fischer R, Wolf S, Nieberle F, Klingelhöffer C, Meier JK, Spoerl S, Ettl T, Reichert TE, Spanier G. Lymph node ratio as a predictor for outcome in oral squamous cell carcinoma: a multicenter population-based cohort study. Clin Oral Investig. 2021 Apr;25(4):1705-1713. doi: 10.1007/s00784-020-03471-6. Epub 2020 Aug 4. PMID: 32754787; PMCID: PMC7966215.

Sridharan S, Thompson LDR, Purgina B, Sturgis CD, Shah AA, Burkey B, Tuluc M, Cognetti D, Xu B, Higgins K, Hernandez-Prera JC, Guerrero D, Bundele MM, Kim S, Duvvuri U, Ferris RL, Gooding WE, Chiosea SI. Early squamous cell carcinoma of the oral tongue with histologically benign lymph nodes: A model predicting local control and vetting of the eighth edition of the American Joint Committee on Cancer pathologic T stage. Cancer. 2019 Sep 15;125(18):3198-3207. doi: 10.1002/cncr.32199. Epub 2019 Jun 7. PMID: 31174238; PMCID: PMC7723468.

Stoop CC, de Bree R, Rosenberg AJWP, van Gemert JTM, Forouzanfar T, Van Cann EM. Locoregional recurrence rate and disease-specific survival following marginal vs segmental resection for oral squamous cell carcinoma with mandibular bone invasion. J Surg Oncol. 2020 Jun 9;122(4):646–52. doi: 10.1002/jso.26054. Epub ahead of print. PMID: 32516499; PMCID: PMC7496367.

Su MC, Chen CT, Huang FI, Chen YL, Jeng YM, Lin CY. Expression of LEF1 is an independent prognostic factor for patients with oral squamous cell carcinoma. J Formos Med Assoc. 2014 Dec;113(12):934-9. doi: 10.1016/j.jfma.2013.07.012. Epub 2013 Sep 7. PMID: 24021930.

Subramaniam N, Balasubramanian D, Murthy S, Kumar N, Vidhyadharan S, Vijayan SN, Nambiar A, Thankappan K, Iyer S. Predictors of locoregional control in stage I/II oral squamous cell carcinoma classified by AJCC 8th edition. Eur J Surg Oncol. 2019 Nov;45(11):2126-2130. doi: 10.1016/j.ejso.2019.05.018. Epub 2019 May 18. PMID: 31196702.

Subramaniam N, Murthy S, Balasubramanian D, Low TH, Vidhyadharan S, Clark JR, Thankappan K, Iyer S. Adverse pathologic features in T1/2 oral squamous cell carcinoma classified by the American Joint Committee on Cancer eighth edition and implications for treatment. Head Neck. 2018 Oct;40(10):2123-2128. doi: 10.1002/hed.25168. Epub 2018 Aug 13. PMID: 30102804.

Szewczyk M, Golusinski W, Pazdrowski J, Masternak M, Sharma N, Golusinski P. Positive fresh frozen section margins as an adverse independent prognostic factor for local recurrence in oral cancer patients. Laryngoscope. 2018 May;128(5):1093-1098. doi: 10.1002/lary.26890. Epub 2017 Oct 8. PMID: 28988423.

Tai SK, Li WY, Yang MH, Chu PY, Wang YF, Chang PM. Perineural invasion as a major determinant for the aggressiveness associated with increased tumor thickness in t1-2 oral tongue and buccal squamous cell carcinoma. Ann Surg Oncol. 2013 Oct;20(11):3568-74. doi: 10.1245/s10434-013-3068-5. Epub 2013 Jul 10. PMID: 23838906.

Takahashi H, Sakakura K, Kudo T, Toyoda M, Kaira K, Oyama T, Chikamatsu K. Cancer-associated fibroblasts promote an immunosuppressive microenvironment through the induction and accumulation of protumoral macrophages. Oncotarget. 2017 Jan 31;8(5):8633-8647. doi: 10.18632/oncotarget.14374. PMID: 28052009; PMCID: PMC5352428.

Tamatani T, Takamaru N, Ohe G, Akita K, Nakagawa T, Miyamoto Y. Expression of CD44, CD44v9, ABCG2, CD24, Bmi-1 and ALDH1 in stage I and II oral squamous cell carcinoma and their association with clinicopathological factors. Oncol Lett. 2018 Jul;16(1):1133-1140. doi: 10.3892/ol.2018.8703. Epub 2018 May 11. PMID: 29963189; PMCID: PMC6019923.

Tan WJ, Chia CS, Tan HK, Soo KC, Iyer NG. Prognostic significance of invasion depth in oral tongue squamous cell carcinoma. ORL J Otorhinolaryngol Relat Spec. 2012;74(5):264-70. doi: 10.1159/000343796. Epub 2012 Nov 2. PMID: 23128365.

Tay ZY, Kao HK, Lien KH, Hung SY, Huang Y, Tsang NM, Chang KP. The impact of preoperative glycated hemoglobin levels on outcomes in oral squamous cell carcinoma. Oral Dis. 2020 Oct;26(7):1449-1458. doi: 10.1111/odi.13433. Epub 2020 Jun 16. PMID: 32426892.

Thiagarajan S, Nair S, Nair D, Chaturvedi P, Kane SV, Agarwal JP, D'Cruz AK. Predictors of prognosis for squamous cell carcinoma of oral tongue. J Surg Oncol. 2014 Jun;109(7):639-44. doi: 10.1002/jso.23583. Epub 2014 Mar 12. PMID: 24619660.

Troiano G, Rubini C, Togni L, Caponio VCA, Zhurakivska K, Santarelli A, Cirillo N, Lo Muzio L, Mascitti M. The immune phenotype of tongue squamous cell carcinoma predicts early relapse and poor prognosis. Cancer Med. 2020 Nov;9(22):8333-8344. doi: 10.1002/cam4.3440. Epub 2020 Oct 13. PMID: 33047888; PMCID: PMC7666743.

Tsuchihashi K, Nakatsugawa M, Kobayashi JI, Sasaya T, Morita R, Kubo T, Kanaseki T, Tsukahara T, Asanuma H, Hasegawa T, Hirano H, Miyazaki A, Hirohashi Y, Torigoe T. Borderline Microenvironment Fibrosis Is a Novel Poor Prognostic Marker of Oral Squamous Cell Carcinoma. Anticancer Res. 2020 Aug;40(8):4319-4326. doi: 10.21873/anticanres.14434. PMID: 32727759.

Walvekar RR, Chaukar DA, Deshpande MS, Pai PS, Chaturvedi P, Kakade AC, D'Cruz AK. Prognostic factors for loco-regional failure in early stage (I and II) squamous cell carcinoma of the gingivobuccal complex. Eur Arch Otorhinolaryngol. 2010 Jul;267(7):1135-40. doi: 10.1007/s00405-009-1201-7. Epub 2010 Jan 12. PMID: 20066453.

Wang HM, Liao CT, Yen TC, Chen SJ, Lee LY, Hsieh CH, Lin CY, Ng SH. Clues toward precision medicine in oral squamous cell carcinoma: utility of next-generation sequencing for the prognostic stratification of high-risk patients harboring neck lymph node extracapsular extension. Oncotarget. 2016 Sep 27;7(39):63082-63092. doi: 10.18632/oncotarget.11762. PMID: 27590518; PMCID: PMC5325348.

Wang K, Veivers D. Tumour thickness as a determinant of nodal metastasis in oral tongue carcinoma. ANZ J Surg. 2017 Sep;87(9):720-724. doi: 10.1111/ans.13515. Epub 2016 Apr 8. PMID: 27061344.

Wei PY, Li WY, Tai SK. Discrete Perineural Invasion Focus Number in Quantification for T1-T2 Oral Squamous Cell Carcinoma. Otolaryngol Head Neck Surg. 2019 Apr;160(4):635-641. doi: 10.1177/0194599818808510. Epub 2018 Dec 18. PMID: 30558519.

Wolfer S, Elstner S, Schultze-Mosgau S. Degree of Keratinization Is an Independent Prognostic Factor in Oral Squamous Cell Carcinoma. J Oral Maxillofac Surg. 2018 Feb;76(2):444-454. doi: 10.1016/j.joms.2017.06.034. Epub 2017 Jun 30. PMID: 28738193.

Wu K, Wei J, Liu Z, Yu B, Yang X, Zhang C, Abdelrehem A, Zhang C, Li S. Can pattern and depth of invasion predict lymph node relapse and prognosis in tongue squamous cell carcinoma. BMC Cancer. 2019 Jul 19;19(1):714. doi: 10.1186/s12885-019-5859-y. PMID: 31324174; PMCID: PMC6642545.

Xie L, Zhou X, Huang W, Chen J, Yu J, Li Z. Facial lymph node involvement as a prognostic factor for patient survival in oral cavity squamous cell carcinoma. Tumour Biol. 2016 Mar;37(3):3489-96. doi: 10.1007/s13277-015-4188-2. Epub 2015 Oct 9. PMID: 26449832.

Xie N, Wang C, Liu X, Li R, Hou J, Chen X, Huang H. Tumor budding correlates with occult cervical lymph node metastasis and poor prognosis in clinical early-stage tongue squamous cell carcinoma. J Oral Pathol Med. 2015 Apr;44(4):266-72. doi: 10.1111/jop.12242. Epub 2014 Aug 28. PMID: 25169851.

Xie N, Yu P, Liu H, Liu X, Hou J, Chen X, Huang H, Wang C. Validation of the International Tumor Budding Consensus Conference (2016) recommendations in oral tongue squamous cell carcinoma. J Oral Pathol Med. 2019 Jul;48(6):451-458. doi: 10.1111/jop.12856. Epub 2019 Apr 15. PMID: 30927486.

Xu B, Salama AM, Valero C, Yuan A, Khimraj A, Saliba M, Zanoni DK, Ganly I, Patel SG, Katabi N, Ghossein R. The prognostic role of histologic grade, worst pattern of invasion, and tumor budding in early oral tongue squamous cell carcinoma: a comparative study. Virchows Arch. 2021 Mar 4:10.1007/s00428-021-03063-z. doi: 10.1007/s00428-021-03063-z. Epub ahead of print. PMID: 33661329; PMCID: PMC8417140.

Xu QS, Wang C, Li B, Li JZ, Mao MH, Qin LZ, Li H, Huang X, Han Z, Feng Z. Prognostic value of pathologic grade for patients with oral squamous cell carcinoma. Oral Dis. 2018 Apr;24(3):335-346. doi: 10.1111/odi.12727. Epub 2017 Sep 18. PMID: 28787551.

Yamakawa N, Kirita T, Umeda M, Yanamoto S, Ota Y, Otsuru M, Okura M, Kurita H, Yamada SI, Hasegawa T, Aikawa T, Komori T, Ueda M; Japan Oral Oncology Group (JOOG). Tumor budding and adjacent tissue at the invasive front correlate with delayed neck metastasis in clinical early-stage tongue squamous cell carcinoma. J Surg Oncol. 2019 Mar;119(3):370-378. doi: 10.1002/jso.25334. Epub 2018 Dec 12. PMID: 30548537; PMCID: PMC6590300.

Yanamoto S, Yamada S, Takahashi H, Kawasaki G, Ikeda H, Shiraishi T, Fujita S, Ikeda T, Asahina I, Umeda M. Predictors of locoregional recurrence in T1-2N0 tongue cancer patients. Pathol Oncol Res. 2013 Oct;19(4):795-803. doi: 10.1007/s12253-013-9646-9. Epub 2013 May 17. PMID: 23677778.

Yanase M, Kato K, Yoshizawa K, Noguchi N, Kitahara H, Nakamura H. Prognostic value of vascular endothelial growth factors A and C in oral squamous cell carcinoma. J Oral Pathol Med. 2014 Aug;43(7):514-20. doi: 10.1111/jop.12167. Epub 2014 Apr 25. PMID: 24762199.

Yang X, Tian X, Wu K, Liu W, Li S, Zhang Z, Zhang C. Prognostic impact of perineural invasion in early stage oral tongue squamous cell carcinoma: Results from a prospective randomized trial. Surg Oncol. 2018 Jun;27(2):123-128. doi: 10.1016/j.suronc.2018.02.005. Epub 2018 Feb 15. PMID: 29937161.

Yoshida S, Shimo T, Murase Y, Takabatake K, Kishimoto K, Ibaragi S, Yoshioka N, Okui T, Nagatsuka H, Sasaki A. The Prognostic Implications of Bone Invasion in Gingival Squamous Cell Carcinoma. Anticancer Res. 2018 Feb;38(2):955-962. doi: 10.21873/anticanres.12309. PMID: 29374727.

Yu EH, Tu HF, Wu CH, Yang CC, Chang KW. MicroRNA-21 promotes perineural invasion and impacts survival in patients with oral carcinoma. J Chin Med Assoc. 2017 Jun;80(6):383-388. doi: 10.1016/j.jcma.2017.01.003. Epub 2017 Feb 27. PMID: 28254348.

Yu P, Wang W, Zhuang Z, Xie N, Xu J, Wang C, Hou J, Han X, Liu X. A novel prognostic model for tongue squamous cell carcinoma based on the characteristics of tumour and its microenvironment: iBD score. Histopathology. 2019 Apr;74(5):766-779. doi: 10.1111/his.13790. Epub 2019 Feb 13. PMID: 30444275.

Zanoni DK, Migliacci JC, Xu B, Katabi N, Montero PH, Ganly I, Shah JP, Wong RJ, Ghossein RA, Patel SG. A Proposal to Redefine Close Surgical Margins in Squamous Cell Carcinoma of the Oral Tongue. JAMA Otolaryngol Head Neck Surg. 2017 Jun 1;143(6):555-560. doi: 10.1001/jamaoto.2016.4238. PMID: 28278337; PMCID: PMC5473778.

Zhang S, Wang X, Gupta A, Fang X, Wang L, Zhang C. Expression of IL-17 with tumor budding as a prognostic marker in oral squamous cell carcinoma. Am J Transl Res. 2019 Mar 15;11(3):1876-1883. PMID: 30972211; PMCID: PMC6456524.

Zhao D, Tang XF, Yang K, Liu JY, Ma XR. Over-expression of integrin-linked kinase correlates with aberrant expression of Snail, E-cadherin and N-cadherin in oral squamous cell carcinoma: implications in tumor progression and metastasis. Clin Exp Metastasis. 2012 Dec;29(8):957-69. doi: 10.1007/s10585-012-9485-1. Epub 2012 May 26. PMID: 22638656.
