# Supplementary material for: The Impact of Histopathological Features on the Prognosis of Oral Squamous Cell Carcinoma: A Comprehensive Review and Meta-Analysis
Source: Front Oncol. 2021 Nov 10;11:784924. doi: 10.3389/fonc.2021.784924 (PMC8631280; doi:10.3389/fonc.2021.784924)
Supplement: Supplementary file 1 [file DataSheet_1.zip › Supplementary Table 3.DOCX]

Supplementary Table 3. Risk of bias of studies included in the meta-analysis.

| Year | Study | Study Participation | Study Attrition | Prognostic Factor Measurement | Outcome Measurement | Study Confounding | Statistical Analysis and Reporting | Overall Score |
| --- | --- | --- | --- | --- | --- | --- | --- | --- |
| 2021 | Moreira et al. | LOW | MODERATE | HIGH | MODERATE | LOW | LOW | MODERATE |
| 2021 | Sambasivan et al. | LOW | MODERATE | MODERATE | LOW | LOW | LOW | LOW |
| 2021 | Nguyen et al. | LOW | HIGH | MODERATE | HIGH | LOW | HIGH | HIGH |
| 2021 | Xu et al. | LOW | MODERATE | LOW | MODERATE | HIGH | LOW | MODERATE |
| 2021 | Kikuchi et al. | LOW | MODERATE | HIGH | LOW | LOW | LOW | MODERATE |
| 2021 | Lau et al. | LOW | MODERATE | MODERATE | MODERATE | LOW | LOW | MODERATE |
| 2021 | Mneimneh et al. | MODERATE | MODERATE | HIGH | MODERATE | LOW | LOW | MODERATE |
| 2020 | Tsuchihashi et al. | LOW | LOW | LOW | HIGH | HIGH | HIGH | HIGH |
| 2020 | Chang et al. | LOW | MODERATE | MODERATE | LOW | HIGH | LOW | MODERATE |
| 2020 | Mascitti et al. | LOW | MODERATE | LOW | LOW | HIGH | LOW | MODERATE |
| 2020 | Dourado et al. | LOW | MODERATE | MODERATE | LOW | LOW | LOW | LOW |
| 2020 | Hoffmann et al. | LOW | LOW | MODERATE | MODERATE | HIGH | LOW | MODERATE |
| 2020 | Lin et al. | LOW | MODERATE | LOW | HIGH | HIGH | LOW | HIGH |
| 2020 | Bajwa et al. | LOW | LOW | MODERATE | HIGH | HIGH | LOW | HIGH |
| 2020 | Mascitti et al. | LOW | MODERATE | LOW | MODERATE | LOW | LOW | LOW |
| 2020 | Lien et al. | LOW | MODERATE | MODERATE | HIGH | HIGH | LOW | HIGH |
| 2020 | Rodrigues et al. | LOW | MODERATE | MODERATE | HIGH | HIGH | LOW | HIGH |
| 2020 | Domingueti et al. | LOW | MODERATE | MODERATE | MODERATE | LOW | LOW | MODERATE |
| 2020 | Hori et al. | LOW | MODERATE | LOW | MODERATE | HIGH | LOW | MODERATE |
| 2020 | Cheng et al. | LOW | MODERATE | HIGH | HIGH | LOW | LOW | HIGH |
| 2020 | Stoop et al. | LOW | MODERATE | MODERATE | MODERATE | LOW | LOW | MODERATE |
| 2020 | Tay et al. | LOW | MODERATE | MODERATE | HIGH | HIGH | LOW | HIGH |
| 2020 | Marinelli et al. | LOW | MODERATE | LOW | MODERATE | HIGH | LOW | MODERATE |
| 2020 | Parekh et al. | LOW | LOW | LOW | HIGH | HIGH | LOW | HIGH |
| 2020 | Troiano et al. | LOW | MODERATE | LOW | MODERATE | HIGH | LOW | MODERATE |
| 2020 | Kurihara-Shimomura et al. | MODERATE | MODERATE | MODERATE | HIGH | HIGH | LOW | HIGH |
| 2020 | Spoerl et al. | LOW | MODERATE | MODERATE | LOW | LOW | LOW | LOW |
| 2019 | Safi et al. | LOW | MODERATE | MODERATE | MODERATE | LOW | LOW | MODERATE |
| 2019 | Chang et al. | LOW | MODERATE | MODERATE | LOW | HIGH | LOW | MODERATE |
| 2019 | Rajappa et al. | LOW | MODERATE | MODERATE | HIGH | HIGH | LOW | HIGH |
| 2019 | Yamakawa et al. | LOW | MODERATE | LOW | HIGH | LOW | LOW | MODERATE |
| 2019 | al.meida et al. | LOW | LOW | MODERATE | MODERATE | LOW | LOW | LOW |
| 2019 | Wu et al. | MODERATE | MODERATE | MODERATE | MODERATE | LOW | LOW | MODERATE |
| 2019 | Heikkinen et al. | MODERATE | MODERATE | LOW | HIGH | HIGH | LOW | HIGH |
| 2019 | Subramaniam et al. | LOW | MODERATE | MODERATE | MODERATE | LOW | LOW | MODERATE |
| 2019 | Al Feghal.i et al. | LOW | LOW | MODERATE | MODERATE | HIGH | LOW | MODERATE |
| 2019 | Ho et al. | LOW | HIGH | LOW | MODERATE | HIGH | LOW | HIGH |
| 2019 | de Paz et al. | LOW | MODERATE | MODERATE | HIGH | HIGH | LOW | HIGH |
| 2019 | Xie et al. | LOW | LOW | LOW | LOW | HIGH | HIGH | HIGH |
| 2019 | Kozak et al. | LOW | MODERATE | LOW | MODERATE | LOW | LOW | LOW |
| 2019 | Wei et al. | LOW | MODERATE | HIGH | LOW | HIGH | LOW | HIGH |
| 2019 | Agarwal. et al. | LOW | MODERATE | MODERATE | MODERATE | HIGH | LOW | MODERATE |
| 2019 | Roh et al. | LOW | MODERATE | MODERATE | LOW | HIGH | LOW | MODERATE |
| 2019 | Yu et al. | LOW | MODERATE | LOW | MODERATE | LOW | HIGH | MODERATE |
| 2019 | de Paz et al. | LOW | MODERATE | MODERATE | HIGH | LOW | LOW | MODERATE |
| 2019 | Sridharan et al. | LOW | MODERATE | MODERATE | LOW | LOW | LOW | LOW |
| 2019 | Hasmat et al. | LOW | MODERATE | HIGH | LOW | LOW | LOW | MODERATE |
| 2019 | Ebihara et al. | LOW | MODERATE | MODERATE | MODERATE | LOW | LOW | MODERATE |
| 2019 | Lu et al. | LOW | MODERATE | LOW | MODERATE | HIGH | LOW | MODERATE |
| 2019 | Zhang et al. | LOW | MODERATE | LOW | MODERATE | HIGH | HIGH | HIGH |
| 2019 | Slieker et al. | LOW | MODERATE | HIGH | MODERATE | HIGH | LOW | HIGH |
| 2019 | Lee, L-Y et al. | LOW | MODERATE | LOW | HIGH | HIGH | LOW | HIGH |
| 2019 | Ding et al. | LOW | MODERATE | MODERATE | HIGH | LOW | LOW | MODERATE |
| 2019 | Rubin et al. | LOW | HIGH | MODERATE | MODERATE | LOW | LOW | MODERATE |
| 2019 | Oliver et al. | LOW | MODERATE | HIGH | MODERATE | LOW | LOW | MODERATE |
| 2019 | Sharma et al. | LOW | MODERATE | MODERATE | HIGH | LOW | LOW | MODERATE |
| 2018 | Nair et al. | LOW | MODERATE | MODERATE | LOW | HIGH | LOW | MODERATE |
| 2018 | Subramaniam et al. | LOW | MODERATE | MODERATE | LOW | LOW | LOW | LOW |
| 2018 | Safi et al. | LOW | MODERATE | LOW | LOW | LOW | LOW | LOW |
| 2018 | Cracchiolo et al. | LOW | MODERATE | LOW | LOW | LOW | LOW | LOW |
| 2018 | Mizrachi et al. | LOW | MODERATE | MODERATE | LOW | HIGH | LOW | MODERATE |
| 2018 | Szewczyk et al. | LOW | MODERATE | LOW | MODERATE | LOW | LOW | LOW |
| 2018 | Wolfer et al. | LOW | MODERATE | MODERATE | MODERATE | HIGH | LOW | MODERATE |
| 2018 | Lee et al. | LOW | MODERATE | MODERATE | MODERATE | HIGH | LOW | MODERATE |
| 2018 | Shukla et al. | LOW | MODERATE | MODERATE | MODERATE | HIGH | LOW | MODERATE |
| 2018 | Xu et al. | LOW | LOW | MODERATE | MODERATE | HIGH | LOW | MODERATE |
| 2018 | al.mangush et al. | MODERATE | MODERATE | LOW | HIGH | HIGH | LOW | HIGH |
| 2018 | Yang et al. | LOW | MODERATE | MODERATE | MODERATE | HIGH | LOW | MODERATE |
| 2018 | Cheng et al. | LOW | MODERATE | HIGH | HIGH | LOW | LOW | HIGH |
| 2018 | Sakata et al. | LOW | MODERATE | LOW | MODERATE | LOW | LOW | LOW |
| 2018 | Yoshida et al. | LOW | MODERATE | MODERATE | MODERATE | HIGH | LOW | MODERATE |
| 2018 | Jing et al. | LOW | MODERATE | LOW | MODERATE | HIGH | LOW | MODERATE |
| 2018 | Pu et al. | LOW | MODERATE | LOW | MODERATE | LOW | LOW | LOW |
| 2018 | Buchakjian et al. | LOW | MODERATE | MODERATE | MODERATE | HIGH | LOW | MODERATE |
| 2018 | Tamatani et al. | MODERATE | MODERATE | MODERATE | HIGH | HIGH | LOW | HIGH |
| 2018 | Miyazaki et al. | MODERATE | MODERATE | LOW | HIGH | HIGH | LOW | HIGH |
| 2017 | Safi et al. | LOW | MODERATE | LOW | LOW | LOW | LOW | LOW |
| 2017 | Niu et al. | LOW | LOW | MODERATE | HIGH | HIGH | HIGH | HIGH |
| 2017 | Ho et al. | LOW | MODERATE | HIGH | LOW | LOW | LOW | MODERATE |
| 2017 | Liu et al. | LOW | MODERATE | MODERATE | LOW | HIGH | LOW | MODERATE |
| 2017 | Lai et al. | LOW | MODERATE | MODERATE | MODERATE | LOW | LOW | MODERATE |
| 2017 | Safi et al. | LOW | MODERATE | MODERATE | MODERATE | HIGH | LOW | MODERATE |
| 2017 | Yu et al. | LOW | MODERATE | MODERATE | MODERATE | HIGH | HIGH | HIGH |
| 2017 | Cassidy et al. | LOW | MODERATE | MODERATE | MODERATE | LOW | LOW | MODERATE |
| 2017 | Takahashi et al. | LOW | MODERATE | MODERATE | HIGH | HIGH | LOW | HIGH |
| 2017 | Petrovic et al. | LOW | MODERATE | MODERATE | MODERATE | HIGH | LOW | MODERATE |
| 2017 | Hasegawa et al. | LOW | MODERATE | MODERATE | MODERATE | HIGH | LOW | MODERATE |
| 2017 | Nagam et al. | MODERATE | MODERATE | MODERATE | MODERATE | HIGH | LOW | MODERATE |
| 2017 | Lee et al. | LOW | MODERATE | MODERATE | LOW | LOW | LOW | LOW |
| 2017 | Zanoni et al. | LOW | MODERATE | LOW | LOW | HIGH | LOW | MODERATE |
| 2017 | Padma et al. | LOW | HIGH | MODERATE | MODERATE | HIGH | LOW | HIGH |
| 2017 | Huang et al. | LOW | MODERATE | MODERATE | HIGH | HIGH | LOW | HIGH |
| 2017 | Hori et al. | LOW | MODERATE | LOW | HIGH | HIGH | LOW | HIGH |
| 2017 | Hosni et al. | LOW | MODERATE | LOW | LOW | LOW | LOW | LOW |
| 2017 | Wang and Veivers | LOW | MODERATE | HIGH | MODERATE | HIGH | HIGH | HIGH |
| 2017 | Satgunaseelan et al. | LOW | MODERATE | MODERATE | MODERATE | LOW | LOW | MODERATE |
| 2017 | Fan, K-H et al. | LOW | MODERATE | MODERATE | LOW | LOW | LOW | LOW |
| 2017 | Nakao et al. | MODERATE | MODERATE | MODERATE | MODERATE | HIGH | LOW | MODERATE |
| 2017 | Jang et al. | LOW | MODERATE | MODERATE | MODERATE | LOW | LOW | MODERATE |
| 2017 | Quinlan-Davidson et al. | LOW | MODERATE | MODERATE | MODERATE | LOW | LOW | MODERATE |
| 2016 | Fives et al. | LOW | MODERATE | LOW | MODERATE | LOW | LOW | LOW |
| 2016 | Heerema et al. | LOW | MODERATE | MODERATE | HIGH | HIGH | LOW | HIGH |
| 2016 | Wang et al. | LOW | MODERATE | MODERATE | MODERATE | LOW | LOW | MODERATE |
| 2016 | Kim et al. | LOW | MODERATE | HIGH | MODERATE | HIGH | LOW | HIGH |
| 2016 | Liu et al. | LOW | MODERATE | MODERATE | LOW | HIGH | LOW | MODERATE |
| 2016 | Lapke et al. | LOW | MODERATE | MODERATE | LOW | HIGH | LOW | MODERATE |
| 2016 | Chen et al. | LOW | MODERATE | MODERATE | MODERATE | LOW | LOW | MODERATE |
| 2016 | Xie et al. | LOW | MODERATE | HIGH | MODERATE | LOW | HIGH | HIGH |
| 2016 | Mücke et al. | LOW | MODERATE | MODERATE | MODERATE | HIGH | HIGH | HIGH |
| 2016 | Heiduschka et al. | LOW | MODERATE | MODERATE | MODERATE | HIGH | LOW | MODERATE |
| 2016 | Adel et al. | LOW | MODERATE | MODERATE | HIGH | HIGH | LOW | HIGH |
| 2016 | Shinagawa et al. | LOW | MODERATE | MODERATE | MODERATE | HIGH | LOW | MODERATE |
| 2015 | Almangush et al. | LOW | MODERATE | LOW | HIGH | HIGH | LOW | HIGH |
| 2015 | Jardim et al. | LOW | MODERATE | MODERATE | MODERATE | LOW | HIGH | MODERATE |
| 2015 | Chen et al. | LOW | MODERATE | MODERATE | LOW | HIGH | HIGH | HIGH |
| 2015 | Chen et al. | LOW | MODERATE | MODERATE | HIGH | LOW | LOW | MODERATE |
| 2015 | Dillon et al. | LOW | MODERATE | MODERATE | MODERATE | LOW | LOW | MODERATE |
| 2015 | Luryi et al. | LOW | MODERATE | MODERATE | MODERATE | HIGH | LOW | MODERATE |
| 2015 | Lin et al. | LOW | MODERATE | MODERATE | HIGH | HIGH | HIGH | HIGH |
| 2015 | Manjula et al. | LOW | HIGH | MODERATE | MODERATE | HIGH | LOW | HIGH |
| 2015 | D'Cruz et al. | LOW | MODERATE | MODERATE | MODERATE | LOW | LOW | MODERATE |
| 2015 | Xie et al. | LOW | MODERATE | LOW | LOW | HIGH | LOW | MODERATE |
| 2015 | Aivazian et al. | LOW | MODERATE | LOW | LOW | HIGH | LOW | MODERATE |
| 2015 | Kong et al. | LOW | MODERATE | MODERATE | MODERATE | HIGH | LOW | MODERATE |
| 2014 | Thiagarajan et al. | LOW | LOW | MODERATE | MODERATE | HIGH | LOW | MODERATE |
| 2014 | Pinto et al. | LOW | HIGH | MODERATE | HIGH | HIGH | LOW | HIGH |
| 2014 | Chatzistefanou et al. | LOW | MODERATE | MODERATE | HIGH | HIGH | LOW | HIGH |
| 2014 | Monteiro et al. | LOW | MODERATE | MODERATE | LOW | LOW | HIGH | MODERATE |
| 2014 | Fan, K-H et al. | LOW | HIGH | MODERATE | LOW | LOW | LOW | MODERATE |
| 2014 | Hedbäck et al. | LOW | MODERATE | MODERATE | MODERATE | HIGH | LOW | MODERATE |
| 2014 | Feng et al. | LOW | LOW | HIGH | LOW | LOW | LOW | MODERATE |
| 2014 | Ebrahimi et al. | LOW | MODERATE | MODERATE | LOW | LOW | LOW | LOW |
| 2014 | Su et al. | LOW | MODERATE | MODERATE | MODERATE | HIGH | HIGH | HIGH |
| 2014 | Yanase et al. | LOW | MODERATE | MODERATE | MODERATE | HIGH | LOW | MODERATE |
| 2013 | Ganly et al. | LOW | MODERATE | MODERATE | MODERATE | LOW | LOW | MODERATE |
| 2013 | Li et al. | LOW | MODERATE | MODERATE | MODERATE | LOW | LOW | MODERATE |
| 2013 | Ling et al. | LOW | MODERATE | MODERATE | MODERATE | LOW | HIGH | MODERATE |
| 2013 | Perisanidis et al. | LOW | MODERATE | MODERATE | LOW | LOW | LOW | LOW |
| 2013 | Durr et al. | LOW | MODERATE | MODERATE | MODERATE | LOW | LOW | MODERATE |
| 2013 | Tai et al. | LOW | MODERATE | MODERATE | LOW | LOW | LOW | LOW |
| 2013 | Amit et al. | LOW | MODERATE | HIGH | MODERATE | LOW | LOW | MODERATE |
| 2013 | Chinn et al. | LOW | MODERATE | MODERATE | MODERATE | LOW | LOW | MODERATE |
| 2013 | Montero et al. | LOW | MODERATE | HIGH | MODERATE | HIGH | LOW | HIGH |
| 2013 | Fried et al. | LOW | MODERATE | HIGH | HIGH | HIGH | LOW | HIGH |
| 2013 | Yanamoto et al. | LOW | MODERATE | LOW | MODERATE | LOW | LOW | LOW |
| 2013 | Chen, T-C et al. | LOW | MODERATE | MODERATE | MODERATE | LOW | LOW | MODERATE |
| 2012 | Ganly et al. | LOW | MODERATE | MODERATE | HIGH | LOW | LOW | MODERATE |
| 2012 | Bachar et al. | LOW | MODERATE | MODERATE | MODERATE | HIGH | LOW | MODERATE |
| 2012 | Grimm et al. | LOW | LOW | MODERATE | LOW | HIGH | LOW | MODERATE |
| 2012 | Petera et al. | LOW | MODERATE | MODERATE | HIGH | LOW | LOW | MODERATE |
| 2012 | Tan et al. | LOW | MODERATE | MODERATE | MODERATE | HIGH | LOW | MODERATE |
| 2012 | Zhao et al. | LOW | LOW | LOW | MODERATE | HIGH | HIGH | HIGH |
| 2011 | Mücke et al. | LOW | LOW | MODERATE | MODERATE | LOW | HIGH | MODERATE |
| 2011 | Jan et al. | LOW | LOW | MODERATE | LOW | LOW | LOW | LOW |
| 2011 | Lin et al. | LOW | HIGH | MODERATE | LOW | LOW | LOW | MODERATE |
| 2011 | Marsh et al. | LOW | MODERATE | LOW | LOW | LOW | LOW | LOW |
| 2011 | Michikawa et al. | LOW | MODERATE | MODERATE | MODERATE | HIGH | LOW | MODERATE |
| 2011 | Chen et al. | LOW | HIGH | MODERATE | HIGH | HIGH | LOW | HIGH |
| 2011 | Camisasca et al. | LOW | MODERATE | MODERATE | MODERATE | LOW | LOW | MODERATE |
| 2011 | Ebrahimi et al. | LOW | MODERATE | LOW | LOW | LOW | LOW | LOW |
| 2010 | Walvekar et al. | LOW | MODERATE | HIGH | HIGH | LOW | LOW | HIGH |
| 2010 | Shim et al. | LOW | MODERATE | MODERATE | MODERATE | LOW | LOW | MODERATE |
| 2010 | Chang, K-P et al. | LOW | LOW | MODERATE | MODERATE | HIGH | HIGH | HIGH |
| 2010 | Kurita et al. | LOW | MODERATE | LOW | HIGH | LOW | LOW | MODERATE |
| 2010 | Fan, K-H et al. | LOW | HIGH | MODERATE | LOW | LOW | HIGH | HIGH |
| 2009 | Larsen et al. | LOW | LOW | MODERATE | HIGH | HIGH | LOW | HIGH |
| 2009 | Goodman et al. | LOW | MODERATE | MODERATE | MODERATE | HIGH | LOW | MODERATE |
| 2007 | Liao et al. | LOW | MODERATE | MODERATE | MODERATE | HIGH | HIGH | HIGH |
| 2005 | Kurokawa et al. | LOW | MODERATE | LOW | HIGH | HIGH | LOW | HIGH |
| 2003 | Chen et al. | LOW | HIGH | MODERATE | MODERATE | HIGH | LOW | HIGH |
| 2000 | Ash et al. | LOW | LOW | MODERATE | HIGH | HIGH | LOW | HIGH |
| 1999 | Högmo et al. | LOW | MODERATE | MODERATE | LOW | LOW | LOW | LOW |
