# Supplementary material for: The Impact of Histopathological Features on the Prognosis of Oral Squamous Cell Carcinoma: A Comprehensive Review and Meta-Analysis
Source: Front Oncol. 2021 Nov 10;11:784924. doi: 10.3389/fonc.2021.784924 (PMC8631280; doi:10.3389/fonc.2021.784924)
Supplement: Supplementary file 1 [file DataSheet_1.zip › Supplementary Table 4.DOCX]

Supplementary Table 4. The certainty of evidence of the histological parameters after assessing the GRADE (the Grading of Recommendations Assessment, Development, and Evaluation).

| Parameter | Outcome | No. of studies | No. of patients | Study design | Risk of bias | Incosistency | Indirectness | Imprecision | Certainty |  |  |
| --- | --- | --- | --- | --- | --- | --- | --- | --- | --- | --- | --- |
| Depth of invasion | |  |  |  |  |  |  |  |  |  |  |
|  | OS | 27 | 7,324 | Observational studies | serious | serious | not serious | not serious | low | ⨁⨁◯◯ |  |
|  | DSS | 11 | 7,781 | Observational studies | serious | serious | not serious | not serious | low | ⨁⨁◯◯ |  |
|  | DFS | 27 | 6,348 | Observational studies | serious | serious | not serious | not serious | low | ⨁⨁◯◯ |  |
| Depth of invasion – cut-off 4 mm | | |  |  |  |  |  |  |  |  |  |
|  | OS | 3 | 388 | Observational studies | not serious | not serious | not serious | serious | very low | ⨁◯◯◯ |  |
|  | DSS | 5 | 3350 | Observational studies | not serious | serious | not serious | not serious | moderate | ⨁⨁⨁◯ |  |
|  | DFS | 8 | 1,486 | Observational studies | not serious | not serious | not serious | not serious | high | ⨁⨁⨁⨁ |  |
| Depth of invasion – cut-off 5 mm | | |  |  |  |  |  |  |  |  |  |
|  | OS | 9 | 3,562 | Observational studies | serious | serious | not serious | not serious | low | ⨁⨁◯◯ |  |
|  | DSS | 3 | 807 | Observational studies | very serious | not serious | not serious | serious | very low | ⨁◯◯◯ |  |
|  | DFS | 4 | 1,485 | Observational studies | not serious | not serious | not serious | not serious | high | ⨁⨁⨁⨁ |  |
| Extranodal extension | |  |  |  |  |  |  |  |  |  |  |
|  | OS | 40 | 48,217 | Observational studies | serious | not serious | not serious | not serious | moderate | ⨁⨁⨁◯ |  |
|  | DSS | 12 | 7460 | Observational studies | serious | not serious | not serious | not serious | moderate | ⨁⨁⨁◯ |  |
|  | DFS | 31 | 12,835 | Observational studies | serious | not serious | not serious | not serious | moderate | ⨁⨁⨁◯ |  |
| Perineural invasion | |  |  |  |  |  |  |  |  |  |  |
|  | OS | 33 | 10,045 | Observational studies | serious | not serious | not serious | not serious | moderate | ⨁⨁⨁◯ |  |
|  | DSS | 26 | 7,523 | Observational studies | not serious | not serious | not serious | not serious | high | ⨁⨁⨁⨁ |  |
|  | DFS | 45 | 15,268 | Observational studies | not serious | not serious | not serious | not serious | high | ⨁⨁⨁⨁ |  |
| Lymphovascular invasion | | |  |  |  |  |  |  |  |  | |
|  | OS | 30 | 30,481 | Observational studies | serious | serious | not serious | not serious | low | ⨁⨁◯◯ |  |
|  | DSS | 13 | 4,411 | Observational studies | not serious | not serious | not serious | not serious | high | ⨁⨁⨁⨁ |  |
|  | DFS | 30 | 8,187 | Observational studies | serious | serious | not serious | not serious | low | ⨁⨁◯◯ |  |
| Surgical margins | |  |  |  |  |  |  |  |  |  |  |
|  | OS | 31 | 63,470 | Observational studies | very serious | very serious | not serious | not serious | very low | ⨁◯◯◯ |  |
|  | DSS | 19 | 20,680 | Observational studies | very serious | very serious | not serious | not serious | very low | ⨁◯◯◯ |  |
|  | DFS | 25 | 15,300 | Observational studies | serious | very serious | not serious | not serious | very low | ⨁◯◯◯ |  |
| Surgical margins - cut-off 5 mm | | |  |  |  |  |  |  |  |  |  |
|  | OS | 10 | 8,659 | Observational studies | not serious | serious | not serious | not serious | moderate | ⨁⨁⨁◯ |  |
|  | DSS | 8 | 6,156 | Observational studies | not serious | serious | not serious | not serious | moderate | ⨁⨁⨁◯ |  |
|  | DFS | 9 | 2,483 | Observational studies | not serious | serious | not serious | not serious | moderate | ⨁⨁⨁◯ |  |
| Tumor thickness | |  |  |  |  |  |  |  |  |  |  |
|  | OS | 5 | 1,651 | Observational studies | very serious | serious | not serious | not serious | very low | ⨁◯◯◯ |  |
|  | DSS | 3 | 638 | Observational studies | serious | serious | not serious | serious | very low | ⨁◯◯◯ |  |
|  | DFS | 4 | 1,556 | Observational studies | very serious | serious | not serious | not serious | very low | ⨁◯◯◯ |  |
| Bone invasion | |  |  |  |  |  |  |  |  |  |  |
|  | OS | 4 | 1,603 | Observational studies | serious | very serious | not serious | not serious | very low | ⨁◯◯◯ |  |
|  | DSS | 5 | 2,773 | Observational studies | serious | serious | not serious | not serious | low | ⨁⨁◯◯ |  |
|  | DFS | 5 | 2,511 | Observational studies | very serious | very serious | not serious | not serious | very low | ⨁◯◯◯ |  |
| Pattern of invasion - Cohesive system | | |  |  |  |  |  |  |  |  |  |
|  | OS | 4 | 543 | Observational studies | not serious | not serious | not serious | serious | moderate | ⨁⨁⨁◯ |  |
|  | DSS | 4 | 1,229 | Observational studies | serious | serious | not serious | not serious | low | ⨁⨁◯◯ |  |
|  | DFS | 4 | 505 | Observational studies | serious | serious | not serious | serious | very low | ⨁◯◯◯ |  |
| Pattern of invasion – Worst-pattern of invasion | | | | |  |  |  |  |  |  |  |
|  | OS | 2 | 420 | Observational studies | not serious | not serious | not serious | very serious | low | ⨁⨁◯◯ |  |
|  | DSS | 2 | 122 | Observational studies | not serious | not serious | not serious | very serious | low | ⨁⨁◯◯ |  |
|  | DFS | 5 | 892 | Observational studies | serious | not serious | not serious | serious | low | ⨁⨁◯◯ |  |
| Tumor budding | |  |  |  |  |  |  |  |  |  |  |
|  | OS | 5 | 986 | Observational studies | serious | serious | not serious | serious | very low | ⨁◯◯◯ |  |
|  | DSS | 5 | 969 | Observational studies | serious | serious | not serious | serious | very low | ⨁◯◯◯ |  |
|  | DFS | 5 | 1,142 | Observational studies | very serious | serious | not serious | not serious | very low | ⨁◯◯◯ |  |
| Tumor-stroma ratio | |  |  |  |  |  |  |  |  |  |  |
|  | OS | 1 | 226 | Observational studies | very serious | not serious | not serious | very serious | very low | ⨁◯◯◯ |  |
|  | DSS | 3 | 724 | Observational studies | serious | not serious | not serious | serious | low | ⨁⨁◯◯ |  |
|  | DFS | 4 | 950 | Observational studies | very serious | not serious | not serious | serious | very low | ⨁◯◯◯ |  |
